# Using *para*hydrogen to hyperpolarize amines, amides, carboxylic acids, alcohols, phosphates, and carbonates

**DOI:** 10.1126/sciadv.aao6250

**Published:** 2018-01-05

**Authors:** Wissam Iali, Peter J. Rayner, Simon B. Duckett

**Affiliations:** Department of Chemistry, University of York, Heslington, York YO10 5DD, UK.

## Abstract

Hyperpolarization turns weak nuclear magnetic resonance (NMR) and magnetic resonance imaging (MRI) responses into strong signals, so normally impractical measurements are possible. We use *para*hydrogen to rapidly hyperpolarize appropriate ^1^H, ^13^C, ^15^N, and ^31^P responses of analytes (such as NH_3_) and important amines (such as phenylethylamine), amides (such as acetamide, urea, and methacrylamide), alcohols spanning methanol through octanol and glucose, the sodium salts of carboxylic acids (such as acetic acid and pyruvic acid), sodium phosphate, disodium adenosine 5′-triphosphate, and sodium hydrogen carbonate. The associated signal gains are used to demonstrate that it is possible to collect informative single-shot NMR spectra of these analytes in seconds at the micromole level in a 9.4-T observation field. To achieve these wide-ranging signal gains, we first use the signal amplification by reversible exchange (SABRE) process to hyperpolarize an amine or ammonia and then use their exchangeable NH protons to relay polarization into the analyte without changing its identity. We found that the ^1^H signal gains reach as high as 650-fold per proton, whereas for ^13^C, the corresponding signal gains achieved in a ^1^H-^13^C refocused insensitive nuclei enhanced by polarization transfer (INEPT) experiment exceed 570-fold and those in a direct-detected ^13^C measurement exceed 400-fold. Thirty-one examples are described to demonstrate the applicability of this technique.

## Teaser

*Para*hydrogen is used to give efficient NMR detection of array of amines, amides, alcohols, carboxylates, carbonates, and phosphates.

## INTRODUCTION

**Scheme 1:**
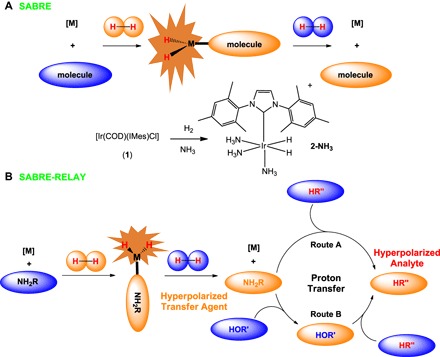
(A) Hyperpolarization via **SABRE** and (B) hyperpolarization via SABRE-RELAY. SABRE is used to hyperpolarize the transfer agent NH_2_R, where R is H or CH_2_Ph or CH_2_CH_2_Ph (etc.), which relays polarization to the analyte (HR″, route A), and R″ is amide, carboxyl, phosphate, or alkoxide (etc.). This process involves both proton exchange and spin-spin interactions and may be mediated by an intermediary HOR′, where R′ is H or suitable scaffold (route B). Center: Reaction scheme shows the formation of SABRE active **2-NH_3_**, which leads to NH_3_.

Nuclear magnetic resonance (NMR) is one of the most powerful methods for the study of materials, and magnetic resonance imaging (MRI) plays a vital role in clinical diagnosis. However, the low sensitivity of these techniques limits their applicability. The hyperpolarization method dynamic nuclear polarization (DNP) improves the detectability of analytes such as pyruvate to the level that the MRI-based diagnosis of disease is now possible (*1*). *Para*hydrogen (*p*-H_2_), which is cheap to prepare and exists as a pure nuclear spin state, was shown to enhance the strength of an NMR signal in 1987 (*2*), although these methods have not yet been used clinically. This may reflect the fact that *p*-H_2_ was originally used to sensitize chemically modified hydrogenation products (*3*, *4*), and only recently has a method been developed where the original identity of the sensitized analyte is retained (*5*). This approach, signal amplification by reversible exchange (SABRE), harnesses *p*-H_2_ in the form of metal-bound hydride ligands and transfers hyperpolarization into a weakly bound substrate (*6*–*8*) via the small *J*-couplings that connect them (*9*). Ligand exchange then builds up a pool of hyperpolarized substrate according to Scheme 1A (*10*). SABRE is successful for analytes with multiple bonds to nitrogen such as nicotinamide (*11*), isoniazid (*12*), pyrazole (*13*), and acetonitrile (*14*), with ^1^H polarizations of 50% (*11*) and ^15^N values of 20% (*15*) being achieved. Furthermore, although it works for other nuclei (*11*, *16*–*20*), it fails to sensitize many classes of analytes.

Here, we describe a method where *p*-H_2_ hyperpolarizes a range of amines, amides, carboxylic acids, alcohols, phosphates, and carbonates without changing their chemical identity. Our method starts with the hyperpolarization of ammonia (the hyperpolarization transfer agent). Subsequently, polarization is relayed into the specified analyte through proton exchange, as outlined in Scheme 1B. Spontaneous low-field transfer then creates the hyperpolarized analyte, which we detect. We called this approach SABRE-RELAY and predict that, when it is fully optimized, it will have a major impact on NMR and MRI in accordance with the fact that we exemplify it for 31 analytes.

## RESULTS

**Fig. 1:**
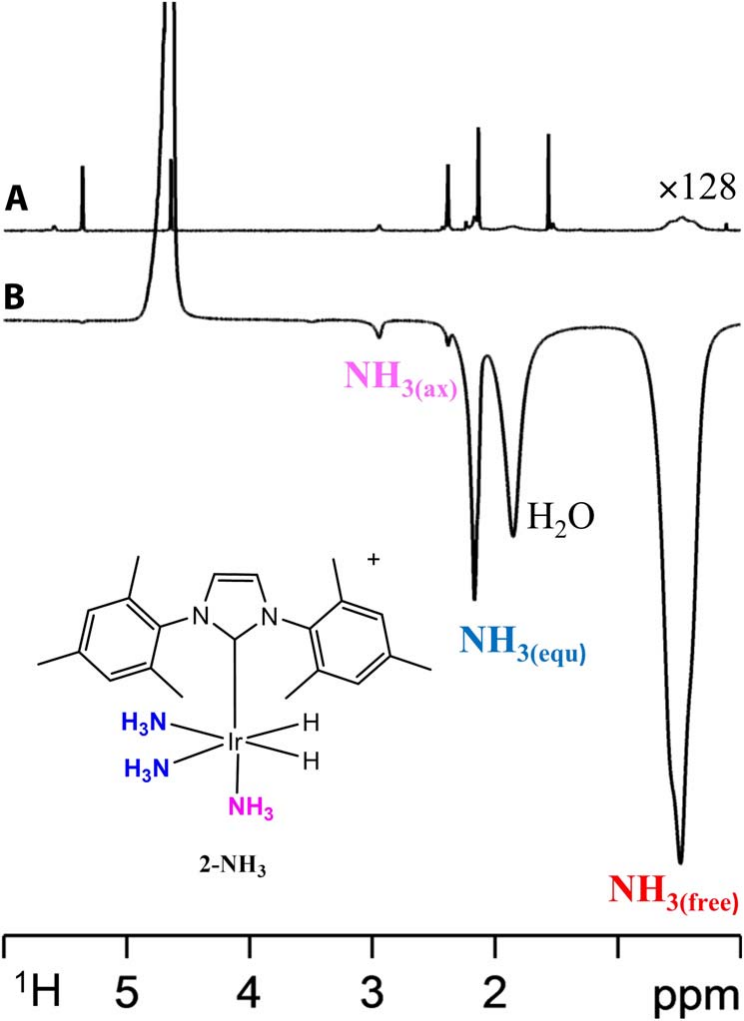
Hyperpolarization of NH_3_ under SABRE. (**A**) Thermally polarized control ^1^H NMR spectrum showing peaks for **2-NH_3_**, NH_3_, and H_2_ at 298 K in dichloromethane-*d*_2_, ×128 vertical expansion relative to (B). (**B**) Corresponding single-scan ^1^H NMR spectrum in the presence of *p*-H_2_, with the hyperpolarized responses for H_2_O, NH_3(free)_, Ir-NH_3(equatorial)_, and Ir-NH_3(axial)_ of **2-NH_3_** indicated.

We achieve SABRE-RELAY by reacting ammonia with the most versatile of the current SABRE catalysts, [IrCl(COD)(IMes)] (*21*, *22*) (*1*) [where IMes is 1,3-bis(2,4,6-trimethylphenyl)imidazol-2-ylidene and COD is cycloocta-1,5-diene] and H_2_, to form [Ir(H)_2_(IMes)(NH_3_)_3_]Cl (**2-NH_3_**) according to Scheme 1. When this reaction is competed in dichloromethane-*d*_2_, **2-NH_3_** exhibits equatorial and axial NH_3_ ligand signals at δ 2.19 and 2.88 in the corresponding ^1^H NMR spectrum, alongside a broad NH_3_ response at δ 0.47, as detailed in Fig. 1A. When this sample is examined after exposure to a 2-bar pressure of *p*-H_2_ gas at 60 G, the resulting ^1^H NMR signal for free NH_3_ now shows an ~10-fold signal enhancement per proton, with the bound NH_3_ ligand signal at δ 2.19 showing a 3-fold enhanced response. These observations confirm that **2-NH_3_** undergoes SABRE to produce hyperpolarized ammonia. When the same process is repeated in methanol-*d*_4_, **2-NH_3_** exhibits a hydride resonance at δ −23.2 that rapidly separates into several components as H-D exchange proceeds to form an array of isotopologues. However, when *p*-H_2_ is used, a hyperpolarized NMR signal is readily seen at δ 5.06 for the exchangeable proton of CD_3_OH, which exhibits a 32-fold intensity gain over its thermally equilibrated signal. Therefore, we added a 5% loading of H_2_O, relative to iridium, to the CD_2_Cl_2_ sample and reexamined it. Under these conditions, the free NH_3_ signal gain resulting from SABRE proved to increase to 40-fold per proton, whereas the corresponding equatorial ligand signal now showed an 85-fold per proton gain (Fig. 1B). In addition, the free H_2_O signal was enhanced by 75-fold per proton, a result that compares well with other solvent signal enhancements (*23*–*25*).

Exchange spectroscopy measurements were then used to confirm that free NH_3_ and the equatorially bound NH_3_ ligand of **2-NH_3_** are in chemical exchange, with the observation of further exchange peaks between free NH_3_ and H_2_O demonstrating the rapid transfer of protons between them. On the basis of this selectivity, we conclude that, when the ammonia is bound, proton exchange between NH_3_ and H_2_O is suppressed because the nitrogen lone pair is involved in bonding to the metal center. Consequently, it now becomes hyperpolarized by SABRE. Proton exchange proceeds, though, after NH_3_ dissociation, and this leads to the observation of hyperpolarization in the chemical exchange–averaged response of H_2_O (or HOCD_3_) according to Scheme 1B. Now, we show how it is possible to harness this proton exchange process to hyperpolarize the NMR signals of a series of added analytes.

**Fig. 2:**
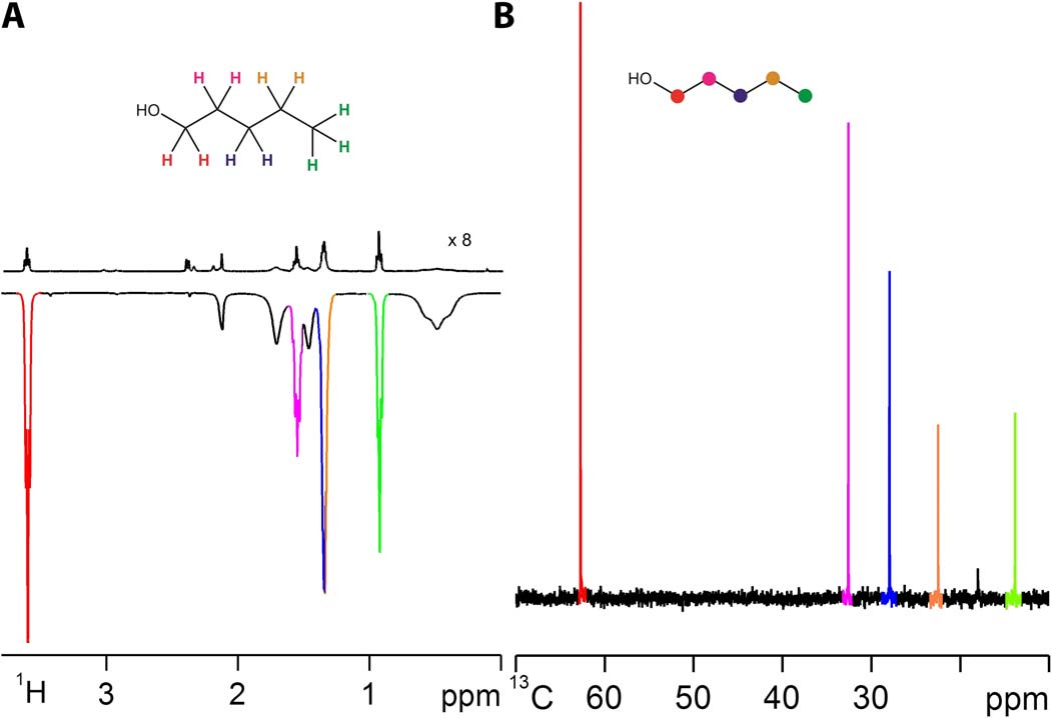
Single-scan NMR spectra of 15.3 mM pentanol (CH_3_CH_2_CH_2_CH_2_CH_2_OH, color-coded structure shown) in dichloromethane-*d*_2_ solution resulting from the action of NH_3_, 2-NH_3_, and *p*-H_2_. (**A**) Upper ^1^H NMR spectrum in the thermally polarized control, ×8 vertical expansion, relative to lower SABRE-RELAY spectrum. (**B**) Single-scan SABRE-RELAY ^1^H-^13^C refocused INEPT NMR spectrum (see fig. S8B for the corresponding thermal control trace).

First, we consider whether the SABRE hyperpolarization of NH_3_ can be relayed into the ^1^H and ^13^C responses of a series of alcohols CH_3_(CH_2_)_*n*_OH (where *n* = 0 to 7). To do this, we prepared a range of dichloromethane-*d*_2_ solutions that contained [Ir(H)_2_(IMes)(NH_3_)_3_]Cl (**2-NH_3_**), NH_3_, and 1 μl of each alcohol (typical concentration, 20 mM). After hyperpolarization transfer from *p*-H_2_, strong signals resulted in the associated single-scan ^1^H NMR spectra, which reached up to 650-fold intensity gains per alcohol CH proton for 1-propanol, averaging at 265 across the series (see the Supplementary Materials). When the same *p*-H_2_ transfer process was undertaken and a fully coupled ^13^C NMR measurement was made instead of a ^1^H NMR measurement, molecule-diagnostic ^13^C and ^1^H-^13^C refocused insensitive nuclei enhanced by polarization transfer (INEPT)–based responses could also be recorded in one scan at 9.4 T for all the alcohols, as illustrated in Fig. 2B for 1-pentanol, with the associated signal gains reaching 570-fold for the C_α_ signal of 1-hexanol. The SABRE-RELAY effect results in the detection of hyperpolarized NMR signals for all the spin-1/2 nuclei in these molecules. In addition, as with SABRE, the hyperpolarized NMR terms reflect a mixture of longitudinal single-spin and higher-order states, whose relative amplitudes depend on the magnetic field that the sample experiences during the polarization transfer step (*16*, *26*). Furthermore, by reducing the concentrations of these analytes below the concentration of NH_3_, it is possible to improve on SABRE-RELAY efficiency. This is beneficial when studying low-concentration analytes because when propanol was studied, the ^1^H NMR signal gains seen for its OH resonance increased by 100% on moving from a 15 to 1.5 mM concentration (fig. S4), whereas its CH resonances showed a ca. 50% improvement in enhancement level; a ^1^H-^13^C refocused INEPT response was still clearly visible in one scan, where the three signals from the OH end were 639, 538, and 603 times larger, respectively, than those in the corresponding ^13^C response. This polarization transfer method is also applicable to complex branched alcohols, and when a sample of ^13^C-labeled glucose was analyzed, a single-scan ^13^C response could be seen for all the expected α and β form signals, which serves to illustrate the wider significance of this effect (fig. S15E). Furthermore, our studies show that, when SABRE-RELAY is carried out under anhydrous conditions with straight-chain alcohols, superior results are obtained.

**Fig. 3:**
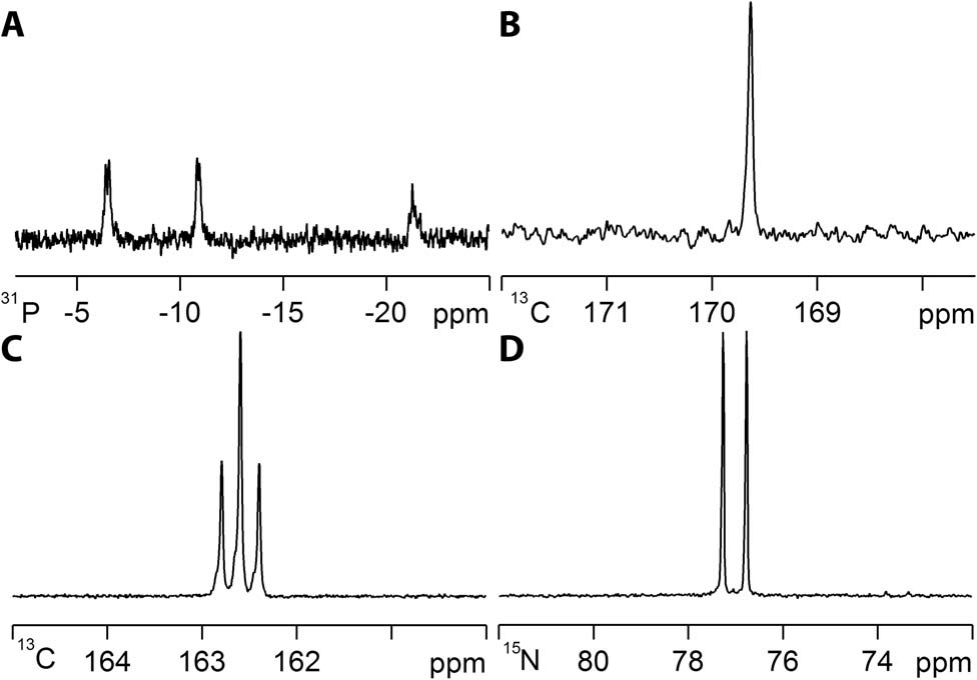
Single-scan SABRE-RELAY NMR spectra recorded in dichloromethane-*d*_2_ with NH_3_ and 2-NH_3_ in the presence of *p*-H_2_. (**A**) Sodium adenosine 5′-triphosphate, ^1^H-^31^P refocused INEPT spectrum (OH transfer) and (**B**) sodium ^13^C–labeled pyruvate, ^13^C NMR spectrum. Single-scan SABRE-RELAY NMR spectra recorded with PEA and **2-PEA** in the presence of *p*-H_2_ for (**C**) ^15^N-^13^C–labeled urea, ^13^C NMR spectrum, 25 mM concentration, and (**D**) ^15^N-^13^C–labeled urea, ^15^N NMR spectrum, 25 mM concentration. The corresponding thermally polarized spectra are detailed in figs. S29A, S18A, S23A, and S23C and yield no signal.

Our next goal was to expand on the range of materials that can be sensitized by this method. We started with pyruvic acid but found that its addition to a solution of **2-NH_3_** and NH_3_ resulted in ammonium salt precipitation, which acted to limit hyperpolarization efficacy. This can be overcome by the addition of a pH modifier such as Cs_2_CO_3_, but working with the corresponding sodium salt proved optimal. When ^13^C-labeled sodium pyruvate, acetate, or propanoic acid samples were studied in the presence of *p*-H_2_, strong ^1^H and ^13^C signals were seen; the ^13^C signal gain for propionic acid was 109-fold. Furthermore, sodium dihydrogen phosphate, adenosine 5′-triphosphate disodium, and ^13^C-labeled sodium hydrogen carbonate provided strong ^31^P and ^13^C responses (Fig. 3, A and B), whereas the amides acetamide, urea, and methacrylamide showed substantial ^1^H, ^13^C, and ^15^N signal gains; for urea, a ^13^C signal gain of 408-fold was observed. These studies could be completed with NH_3_/H_2_O or NH_3_/CH_3_OH, as detailed in the Supplementary Materials, to promote the necessary proton exchange, and the observations establish that analytes containing the four common functional groups—OH, NH_2_CO, POH, and COOH—can be used. In some cases, we see evidence for Schiff-base condensation at long reaction times but could suppress this process by adding water.

**Fig. 4:**
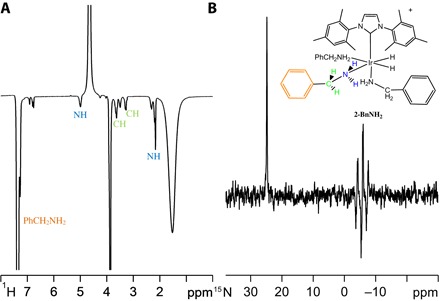
Hyperpolarization of benzylamine (BnNH_2_) under SABRE. (**A**) ^1^H NMR spectrum of hyperpolarized BnNH_2_ achieved via **2-BnNH_2_** (top right) under SABRE in dichloromethane-*d*_2_ solution after transfer at 60 G (see fig. S32A for the corresponding thermally equilibrated NMR spectrum). The enhanced signals are color-coded; NH_2_ (blue), CH_2_ (light green), and Ph (orange) for the bound equatorial BnNH_2_ ligand of **2-BnNH_2_**, NH_2_ and CH_2_ of the free material, and H_2_O. (**B**) Corresponding ^15^N NMR spectrum recorded using ^15^N-labeled BnNH_2_ after transfer in a μ-metal shield showing the free (left) and equatorial ligand (right) responses.

To examine the role of the hyperpolarization transfer agent, we replaced NH_3_ with benzylamine (BnNH_2_) or phenethylamine (PEA). Both react with **1** and H_2_, forming [Ir(H)_2_(IMes)(NH_2_Bn)_3_]Cl (**2-BnNH_2_**) and [Ir(H)_2_(IMes)(PEA)_3_]Cl (**2-PEA**), respectively. For the corresponding **2-BnNH_2_** sample, signal gains for free BnNH_2_ of 72-fold (NH), 53-fold (CH), and 170-fold (aromatic), respectively, per proton are observed (Fig. 4), and these measurements can be repeated if the same sample is probed with *p*-H_2_ several days after the first observation was made. PEA proved to perform better than BnNH_2_, with the corresponding NH_2_ signal gain being 108-fold per proton for a 10-fold loading of **1** with signal gains of 50-fold (NCH_2_), 45-fold (CH_2_), 92-fold (*ortho*), 50-fold (*meta*), and 20-fold (*para*) resulting for the other groups. These observations show how polarization transfer through the aliphatic carbon chain into the aromatic protons is possible. BnNH_2_ and PEA also proved suitable for SABRE-RELAY. In the case of PEA, the efficiency of urea hyperpolarization was found to improve (Fig. 3, C and D) over that achieved with NH_3_, although the measured response of ^13^C-labeled glucose was found to reduce. Furthermore, replacing BnNH_2_ with its *d*_7_-form, C_6_D_5_CD_2_NH_2_, led to further improvements in observed analyte response level because the initially created SABRE hyperpolarization was now optimally focused into just the NH_2_ protons.

Given the wide range of amine p*K*_b_ values (*27*), it may be possible to remove the need for an auxiliary base when dealing with acidic analytes through a process of amine variation. Therefore, we conclude that studies on the role of the amine will be important for the optimization of SABRE-RELAY and may even allow the introduction of selectivity into the hyperpolarization process. Furthermore, because improvements in analyte detectability with SABRE can be easily achieved by varying the polarization transfer field, reducing relaxation within the analyte, and optimizing the catalyst lifetime while minimizing its relaxivity, we expect the signal gains reported here to be similarly improved upon in the future (*5*).

## DISCUSSION

In summary, we have shown that SABRE-RELAY can be used to hyperpolarize a wide range of biologically relevant materials. In the initial step, SABRE is used to enhance the NH proton response of the selected hyperpolarized transfer agent (the free amine) by between 10- and 120-fold per proton. When this is achieved in the presence of propanol, proton exchange results in its OH signal being amplified by between 250- and 500-fold. The nonequilibrium magnetic state of the OH proton is then successfully relayed into its aliphatic ^1^H resonances such that the corresponding signals are amplified by between 650- and 790-fold per proton. We used this ^1^H signal gain to record a single-scan ^1^H-^13^C refocused INEPT NMR spectrum using just 1 × 10^−7^ moles of material, although direct transfer to ^13^C means that a weaker fully coupled ^13^C response can also be seen. On the basis of these signal gains, we hope that this route can be developed to allow the phenotyping of urine via lower-field ^13^C detection in the future as an alternative to the current high-field ^1^H detection methods (*28*). However, because exchangeable protons feature heavily in biochemical NMR, we expect harnessing this effect to be of significant interest to biochemists, especially if it is augmented with high-field transfer via the “low-irradiation generation of high tesla–SABRE” approach (*29*). In addition, because hyperpolarized urea, glucose, and pyruvate reflect successful MRI probes of disease (*30*, *31*), when SABRE-RELAY is coupled with catalyst removal and biocompatibility, we expect this route to become clinically important because it can theoretically deliver a continuously hyperpolarized bolus (*32*). Moreover, because studies of catalysis with *p*-H_2_ have made significant contributions to process optimization (*33*–*37*), we expect this approach to provide insight into important reactions such as transfer hydrogenation (*38*), hydroamination (*39*), and N_2_ fixation in the future (*40*).

## MATERIALS AND METHODS

### Experimental design

The measurements undertaken in this work were completed on a 400-MHz Avance III spectrometer and involved ^1^H, ^13^C, ^15^N, and ^31^P detection, as detailed in the Supplementary Materials. Enhancement values were determined according to the methods defined here, and sample details allowed the repetition of these measurements, which involved the following procedures.

#### SABRE-RELAY polarization transfer method with NH_3_

The polarization transfer experiments that were reported in this study were conducted in 5-mm NMR tubes that were equipped with a J. Young’s tap. Samples for these polarization transfer experiments were based on a 5 mM solution of [IrCl(COD)(IMes)] and the indicated substrate and NH_3_ loadings in methanol-*d*_4_ or dichloromethane-*d*_2_ (0.6 ml). The samples were degassed before the introduction of NH_3_. Subsequently, *p*-H_2_ at a pressure of ca. 3 bar was added. Then, samples were shaken for 10 s in the specified fringe field of an NMR spectrometer before they were rapidly transported into the magnet for subsequent interrogation by NMR spectroscopy. This whole process takes ca. 15 s to achieve.

#### SABRE-RELAY polarization transfer method with BnNH_2_ or PEA

The polarization transfer experiments that were reported were conducted in 5-mm NMR tubes that were equipped with a J. Young’s tap. Samples for these polarization transfer experiments were based on a 5 mM solution of [IrCl(COD)(IMes)], the indicated BnNH_2_ or PEA loading, and the indicated additional substrate at the specified loading in dichloromethane-*d*_2_ (0.6 ml). The samples were degassed before the introduction of *p*-H_2_ at a pressure of ca. 3 bar. Samples were then shaken for 10 s in the specified fringe field of an NMR spectrometer before they were rapidly transported into the magnet for subsequent interrogation by NMR spectroscopy.

## Supplementary Material

http://advances.sciencemag.org/cgi/content/full/4/1/eaao6250/DC1

## SUPPLEMENTARY MATERIALS

Supplementary material for this article is available at http://advances.sciencemag.org/cgi/content/full/4/1/eaao6250/DC1 section S1. SABRE-RELAY polarization transfer method with NH_3_ section S2. SABRE-RELAY polarization transfer method with BnNH_2_ or PEA section S3. Polarization enhancement quantification procedures section S4. NMR spectrometer details section S5. Pulse sequence details section S6. SABRE-RELAY spectra fig. S1. INEPT pulse sequence. fig. S2. DEPT pulse sequence. fig. S3. SABRE-RELAY NMR spectra methanol. fig. S4. SABRE-RELAY NMR spectra ethanol. fig. S5. SABRE-RELAY NMR spectra propanol. fig. S6. SABRE-RELAY NMR spectra propanol, low concentration. fig. S7. SABRE-RELAY NMR spectra butanol. fig. S8. SABRE-RELAY NMR spectra pentanol. fig. S9. SABRE-RELAY NMR spectra hexanol. fig. S10. SABRE-RELAY NMR spectra heptanol. fig. S11. SABRE-RELAY NMR spectra octanol. fig. S12. SABRE-RELAY NMR spectra isopropanol. fig. S13. SABRE-RELAY NMR spectra *tert*-butanol. fig. S14. SABRE-RELAY NMR spectra d-glucose. fig. S15. SABRE-RELAY NMR spectra d-glucose-^13^C. fig. S16. SABRE-RELAY NMR spectra glycerol. fig. S17. SABRE-RELAY NMR spectra sodium acetate-^13^C. fig. S18. SABRE-RELAY NMR spectra sodium pyruvate-^13^C. fig. S19. SABRE-RELAY NMR spectra sodium acetate-1,2 ^13^C_2_. fig. S20. SABRE-RELAY NMR spectra propionic acid-^13^C. fig. S21. SABRE-RELAY NMR spectra sodium hydrogen carbonate-^13^C. fig. S22. SABRE-RELAY NMR spectra urea-^13^C. fig. S23. SABRE-RELAY NMR spectra urea-^13^C-^15^N_2_. fig. S24. SABRE-RELAY NMR spectra urea-^13^C-^15^N_2_. fig. S25. SABRE-RELAY NMR spectra acetamide. fig. S26. SABRE-RELAY NMR spectra methacrylamide. fig. S27. SABRE-RELAY NMR spectra cyclohexyl methacrylamide. fig. S28. SABRE-RELAY NMR spectra mono sodium dihydrogen orthophosphate. fig. S29. SABRE-RELAY NMR spectra adenosine 5′-triphosphate disodium salt. fig. S30. SABRE-RELAY NMR spectra ammonia in methanol. fig. S31. SABRE-RELAY NMR spectra ammonia in dichloromethane. fig. S32. SABRE-RELAY NMR spectra benzylamine. fig. S33. SABRE-RELAY NMR spectra benzylamine-^15^N. fig. S34. SABRE-RELAY NMR spectra, mixture of urea, propanol, and PEA. table S1. Alcohol ^1^H SABRE-RELAY signal enhancement values. table S2. Alcohol ^13^C SABRE-RELAY signal enhancement values. table S3. NMR data for **2-NH_3_**. table S4. NMR data for **2-BnNH_2_**.
