# Supplementary material for: Using parahydrogen to hyperpolarize amines, amides, carboxylic acids, alcohols, phosphates, and carbonates
Source: Sci Adv. 2018 Jan 5;4(1):eaao6250. doi: 10.1126/sciadv.aao6250 (PMC5756661; doi:10.1126/sciadv.aao6250)
Supplement: http://advances.sciencemag.org/cgi/content/full/4/1/eaao6250/DC1 [file supp_4_1_eaao6250__index.html]

Science Advances | Science Advances

## Supplementary Materials

**This PDF file includes:**

- section S1. SABRE-RELAY polarization transfer method with NH3
- section S2. SABRE-RELAY polarization transfer method with BnNH2 or PEA
- section S3. Polarization enhancement quantification procedures
- section S4. NMR spectrometer details
- section S5. Pulse sequence details
- section S6. SABRE-RELAY spectra
- fig. S1. INEPT pulse sequence.
- fig. S2. DEPT pulse sequence.
- fig. S3. SABRE-RELAY NMR spectra methanol.
- fig. S4. SABRE-RELAY NMR spectra ethanol.
- fig. S5. SABRE-RELAY NMR spectra propanol.
- fig. S6. SABRE-RELAY NMR spectra propanol, low concentration.
- fig. S7. SABRE-RELAY NMR spectra butanol.
- fig. S8. SABRE-RELAY NMR spectra pentanol.
- fig. S9. SABRE-RELAY NMR spectra hexanol.
- fig. S10. SABRE-RELAY NMR spectra heptanol.
- fig. S11. SABRE-RELAY NMR spectra octanol.
- fig. S12. SABRE-RELAY NMR spectra isopropanol.
- fig. S13. SABRE-RELAY NMR spectra *tert*-butanol.
- fig. S14. SABRE-RELAY NMR spectra D-glucose.
- fig. S15. SABRE-RELAY NMR spectra D-glucose-13C.
- fig. S16. SABRE-RELAY NMR spectra glycerol.
- fig. S17. SABRE-RELAY NMR spectra sodium acetate-13C.
- fig. S18. SABRE-RELAY NMR spectra sodium pyruvate-13C.
- fig. S19. SABRE-RELAY NMR spectra sodium acetate-1,2 13C2.
- fig. S20. SABRE-RELAY NMR spectra propionic acid-13C.
- fig. S21. SABRE-RELAY NMR spectra sodium hydrogen carbonate-13C.
- fig. S22. SABRE-RELAY NMR spectra urea-13C.
- fig. S23. SABRE-RELAY NMR spectra urea-13C-15N2.
- fig. S24. SABRE-RELAY NMR spectra urea-13C-15N2.
- fig. S25. SABRE-RELAY NMR spectra acetamide.
- fig. S26. SABRE-RELAY NMR spectra methacrylamide.
- fig. S27. SABRE-RELAY NMR spectra cyclohexyl methacrylamide.
- fig. S28. SABRE-RELAY NMR spectra mono sodium dihydrogen orthophosphate.
- fig. S29. SABRE-RELAY NMR spectra adenosine 5′-triphosphate disodium salt.
- fig. S30. SABRE-RELAY NMR spectra ammonia in methanol.
- fig. S31. SABRE-RELAY NMR spectra ammonia in dichloromethane.
- fig. S32. SABRE-RELAY NMR spectra benzylamine.
- fig. S33. SABRE-RELAY NMR spectra benzylamine-15N.
- fig. S34. SABRE-RELAY NMR spectra, mixture of urea, propanol, and PEA.
- table S1. Alcohol 1H SABRE-RELAY signal enhancement values.
- table S2. Alcohol 13C SABRE-RELAY signal enhancement values.
- table S3. NMR data for **2-NH3**.
- table S4. NMR data for **2-BnNH2**.

Download PDF

**Files in this Data Supplement:**

- Adobe PDF - aao6250\_SM.pdf
