# Supplementary material for: Using parahydrogen to hyperpolarize amines, amides, carboxylic acids, alcohols, phosphates, and carbonates
Source: Sci Adv. 2018 Jan 5;4(1):eaao6250. doi: 10.1126/sciadv.aao6250 (PMC5756661; doi:10.1126/sciadv.aao6250)
Supplement: http://advances.sciencemag.org/cgi/content/full/4/1/eaao6250/DC1 [file aao6250_SM.pdf]

Science Advances

AAAS

advances.sciencemag.org/cgi/content/full/4/1/eaao6250/DC1

Supplementary Materials for

Using *para*hydrogen to hyperpolarize amines, amides, carboxylic acids, alcohols, phosphates, and carbonates

Wissam Iali, Peter J. Rayner, Simon B. Duckett

Published 5 January 2018, *Sci. Adv.* **4**, eaao6250 (2018)
DOI: 10.1126/sciadv.aao6250

This PDF file includes:

- section S1. SABRE-RELAY polarization transfer method with NH_3
- section S2. SABRE-RELAY polarization transfer method with $BnNH_2$ or PEA
- section S3. Polarization enhancement quantification procedures
- section S4. NMR spectrometer details
- section S5. Pulse sequence details
- section S6. SABRE-RELAY spectra
- fig. S1. INEPT pulse sequence.
- fig. S2. DEPT pulse sequence.
- fig. S3. SABRE-RELAY NMR spectra methanol.
- fig. S4. SABRE-RELAY NMR spectra ethanol.
- fig. S5. SABRE-RELAY NMR spectra propanol.
- fig. S6. SABRE-RELAY NMR spectra propanol, low concentration.
- fig. S7. SABRE-RELAY NMR spectra butanol.
- fig. S8. SABRE-RELAY NMR spectra pentanol.
- fig. S9. SABRE-RELAY NMR spectra hexanol.
- fig. S10. SABRE-RELAY NMR spectra heptanol.
- fig. S11. SABRE-RELAY NMR spectra octanol.
- fig. S12. SABRE-RELAY NMR spectra isopropanol.
- fig. S13. SABRE-RELAY NMR spectra *tert*-butanol.
- fig. S14. SABRE-RELAY NMR spectra D-glucose.
- fig. S15. SABRE-RELAY NMR spectra D-glucose-^{13}C.
- fig. S16. SABRE-RELAY NMR spectra glycerol.
- fig. S17. SABRE-RELAY NMR spectra sodium acetate-^{13}C.
- fig. S18. SABRE-RELAY NMR spectra sodium pyruvate-^{13}C.

- fig. S19. SABRE-RELAY NMR spectra sodium acetate-1,2 $^{13}C2$.
- fig. S20. SABRE-RELAY NMR spectra propionic acid-^{13}C.
- fig. S21. SABRE-RELAY NMR spectra sodium hydrogen carbonate-^{13}C.
- fig. S22. SABRE-RELAY NMR spectra urea-^{13}C.
- fig. S23. SABRE-RELAY NMR spectra urea-^{13}C-$^{15}N2$.
- fig. S24. SABRE-RELAY NMR spectra urea-^{13}C-$^{15}N2$.
- fig. S25. SABRE-RELAY NMR spectra acetamide.
- fig. S26. SABRE-RELAY NMR spectra methacrylamide.
- fig. S27. SABRE-RELAY NMR spectra cyclohexyl methacrylamide.
- fig. S28. SABRE-RELAY NMR spectra mono sodium dihydrogen orthophosphate.
- fig. S29. SABRE-RELAY NMR spectra adenosine 5′-triphosphate disodium salt.
- fig. S30. SABRE-RELAY NMR spectra ammonia in methanol.
- fig. S31. SABRE-RELAY NMR spectra ammonia in dichloromethane.
- fig. S32. SABRE-RELAY NMR spectra benzylamine.
- fig. S33. SABRE-RELAY NMR spectra benzylamine-^{15}N.
- fig. S34. SABRE-RELAY NMR spectra, mixture of urea, propanol, and PEA.
- table S1. Alcohol ^{1}H SABRE-RELAY signal enhancement values.
- table S2. Alcohol ^{13}C SABRE-RELAY signal enhancement values.
- table S3. NMR data for **2-NH_3**.
- table S4. NMR data for **2-$BnNH_2$**.

section S1. SABRE-RELAY polarization transfer method with NH_3

The polarization transfer experiments that are reported in this study were conducted in 5 mm NMR tubes that were equipped with a J. Young's Tap. Samples for these polarization transfer experiments were based on a 5 mM solution of [IrCl(COD)(IMes)] and the indicated substrate and NH_3 loadings in methanol-d_4 or dichloromethane-d_2 (0.6 mL). The samples were degassed prior to the introduction of NH_3.Subsequently, *para*hydrogen at a pressure of ca. 3 bar was added. Samples were then shaken for 10 s in the specified fringe field of an NMR spectrometer before being rapidly transported into the magnet for subsequent interrogation by NMR spectroscopy. This whole process takes ca. 15 seconds to achieve.

section S2. SABRE-RELAY polarization transfer method with $BnNH_2$ or PEA

The polarization transfer experiments that are reported were conducted in 5 mm NMR tubes that were equipped with a J. Young's Tap. Samples for these polarization transfer experiments were based on a 5 mM solution of [IrCl(COD)(IMes)],the indicated $BnNH_2$ or PEA loading and the indicated additional substrate at the specified loading in dichloromethane-d_2 (0.6 mL). The samples were degassed prior to the introduction of *para*hydrogen at a pressure of ca. 3 bar. Samples were then shaken for 10 s in the specified fringe field of an NMR spectrometer before being rapidly transported into the magnet for subsequent interrogation by NMR spectroscopy.

section S3. Polarization enhancement quantification procedures

For calculation of the ^{1}H and ^{31}P signal enhancements the following formula was used

$$E = \frac{SI(pol)}{SI(unpol)}$$

Where, E = enhancement level, SI(pol) = signal of polarized sample, SI(unpol) = signal of unpolarized (reference) sample. Experimentally, both spectra were recorded on the same sample using identical acquisition parameters, including the receiver gain. The raw integrals of the relevant resonances in the polarized and unpolarized spectra were then used to determine the enhancement levels. The quoted values reflect the signal strength gain (fold) per proton/phosphorus nucleus in the specified group.

^{13}C enhancements were calculated by taking the raw integral of the $^{13}CD_2Cl_2$ peak observed from the solvent in the sample after equilibration inside the magnet for 1 minute. CD_2Cl_2 was present in each sample at a concentration of 15.67 M and the resulting SABRE-Relay hyperpolarized signal was then scaled according to the concentration of substrate in solution

(given in section S2) to give the final enhancement value for ^{13}C. These values were corroborated by further measurements on samples containing $CHCl_3$ as an internal standard.

section S4. NMR spectrometer details

Spectra were typically acquired on a 400 MHz Bruker, Avance III console using a 5 mm BBI probe which was tuned to ^{1}H, ^{13}C, ^{31}P or ^{15}N as specified. Resonances are referenced relative to the residual proton signal of the indicated deuterated solvent.

section S5. Pulse sequence details

Refocused INEPT

INEPT based measurements for ^{13}C, ^{31}P and ^{15}N were recorded using a standard refocused refocused INEPT experiment with decoupling during acquisition as outlined in fig. S1. The evolution delay (d4) is optimized to 1/2 x J_{XH} and obtains in-phase X-magnetization through the addition of a refocusing delay (d3) which was optimized for 1/(6 x J_{XH}). Values used for J_{XH} are given in section S2.

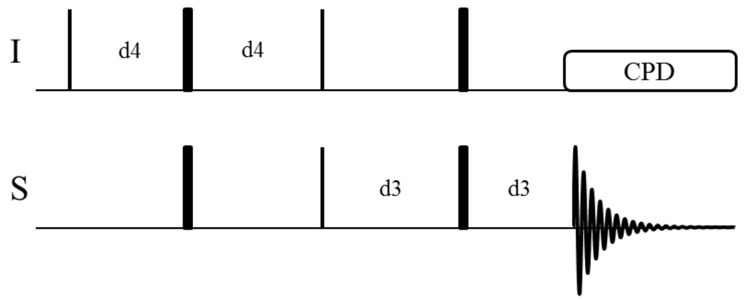

fig. S1. INEPT pulse sequence. Ineptrd pulse sequence utilized for transferring SABRE-Relay derived ^{1}H-polarization to heteronuclei.

DEPT

DEPT based measurements for ^{13}C were made using the standard Bruker pulse sequence shown in fig. S2. Φ = 135° in all cases and J_{XH} values are given in section S2. Signals from CH_2 groups are observed as negative whereas CH and CH_3 groups are positive.

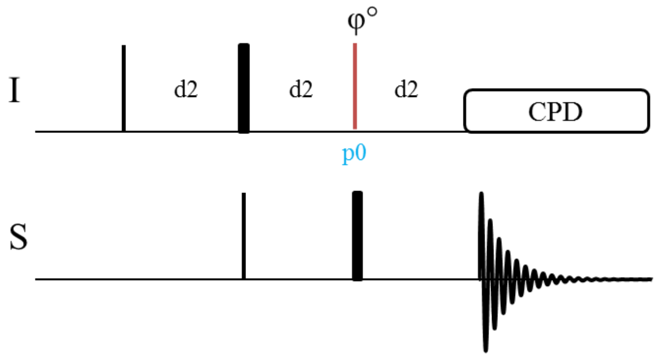

fig. S2. DEPT pulse sequence. Dept-135 pulse sequence utilized for transferring SABRE-Relay derived ^{1}H-polarization to ^{13}C nuclei.

section S6. SABRE-RELAY spectra

Alcohols

The SABRE-Relay polarization of a series of straight chain alcohols was achieved. The ^{1}H signal enhancements detailed in table S1, show that the OCH proton environment is most strongly hyperpolarized in each case. As the length of the carbon chain increases, the enhancement factor per proton reduces due to increased spin dilution however total ^{1}H enhancements are maintained at over 1600-fold.SABRE-Relay is also observed in branched alcohols such as isopropanol and tert-butanol. The values quoted reflect the average of at least three measurements and the effects are fully reproducible.

table S1. Alcohol ^{1}H SABRE-RELAY signal enhancement values. ^{1}H enhancement factors for a series of alcohols. Enhancement values are per proton and total enhancements are normalized to reflect the number of protons in each molecule.

Alcohol	OH	OCH	-(CH_2)$_n$-	CH_3	Total
Methanol	150	495	-	-	1635
Ethanol	242	497	-	574	2958
1-Propanol	223	653	637	539	4420
1-Butanol	474	494	396	444	3490
1-Pentanol	134	296	143	209	2211
1-Hexanol	179	327	149	212	2237
1-Heptanol	191	253	107	142	2193
1-Octanol	125	274	98	171	2020
Isopropanol	983	416	-	332	3391
Tert-Butanol	480	n/a	-	154	1866

Strong polarization transfer was also overserved in the ^{13}C and ^{1}H-^{13}C refocused INEPT spectra that were collected alongside these data. The ^{13}C SABRE-Relay NMR spectra give rise to antiphase peaks as shown that are separated according to their J_{CH} couplings. We do not quantify the enhancement gains for these traces as a consequence, although we note visible signals can be seen. In contrast, in-phase magnetization is readily achieved by using a ^{1}H-^{13}C refocused INEPT, and now the enhancement factors in table S2 are quoted relative to the expected ^{13}C signal intensity in the corresponding $^{13}C\{^{1}H\}$ experiment that is recorded without nOe build-up. In all cases, the O$\underline{C}$H resonance gave the strongest signal gain.

table S2. Alcohol ^{13}C SABRE-RELAY signal enhancement values. ^{13}C enhancement factors for ^{1}H-^{13}C-refocused INEPT.

Alcohol	C1	C2	C3	C4	C5	C6	C7	C8
Methanol	185	-	-	-	-	-	-	-
Ethanol	335	236	-	-	-	-	-	-
1-Propanol	235	371	124	-	-	-	-	-
1-Butanol	285	199	181	117	-	-	-	-
1-Pentanol	329	242	160	77	95	-	-	-
1-Hexanol	574	315	9	189	74	115		-
1-Heptanol	454	260	4	137	73	54	45	-
1-Octanol	429	196	20	5	31	120	30	69
Isopropanol	229	128	-	-	-	-	-	-

Methanol

fig. S3. SABRE-RELAY NMR spectra methanol. (**a** to **e**) reflect a series of single scan NMR spectra: (a) ^{1}H NMR spectrum, thermally polarized (recorded under Boltzmann equilibrium conditions for the associated NMR spin state populations), (b) ^{1}H NMR spectrum, via SABRE-RELAY, (c) ^{13}C NMR spectrum thermally polarized, (d) ^{13}C NMR spectrum via SABRE-RELAY, (e) ^{1}H-^{13}C refocused INEPT NMR spectrum after SABRE-RELAY and (**f**) ^{13}C DEPT NMR spectrum after SABRE-RELAY. All NMR spectra are designed to show the signals of the added methanol. In the ^{1}H NMR spectrum, the OH peak at around 5 ppm, its CH_3 resonance at 3.4 ppm and the NH_3 signals at ca 0.5 ppm are strongly enhanced. The ^{13}C spectra establish that the $\underline{C}H_3$ signal is also visible.

Conditions: [IrCl(COD)(IMes)] (5 mM), methanol (1 uL.),CD_2Cl_2 (0.6 mL), NH_3; Substrate Concentration: 41 mM. Nominal ^{1}H signal enhancement levels (fold/per proton): 150 (OH), 495 (-OCH_3) after SABRE-RELAY at 60 G.

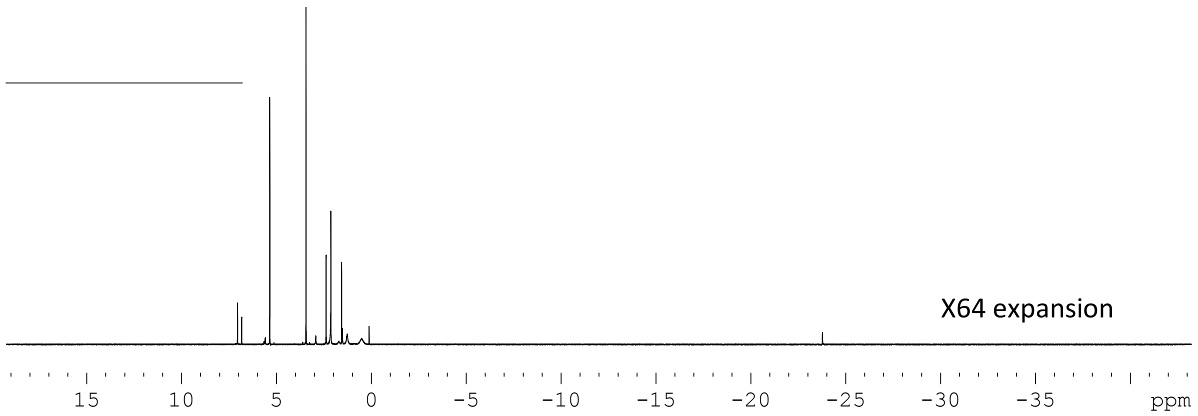

fig. S3a. ^{1}H NMR spectrum, thermally polarized, methanol doped sample.

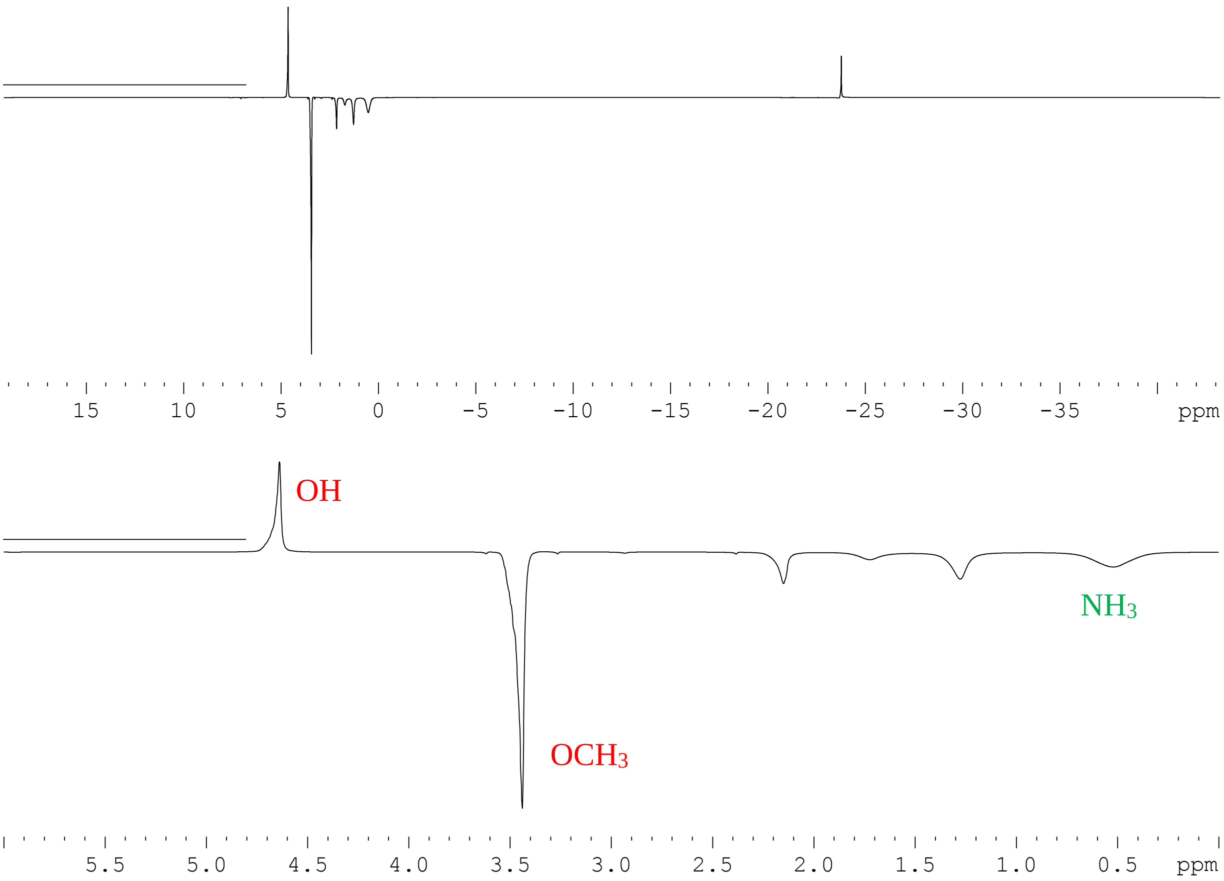

fig. S3b. ^{1}H SABRE-RELAY NMR spectrum, methanol (top) with expansion (bottom).

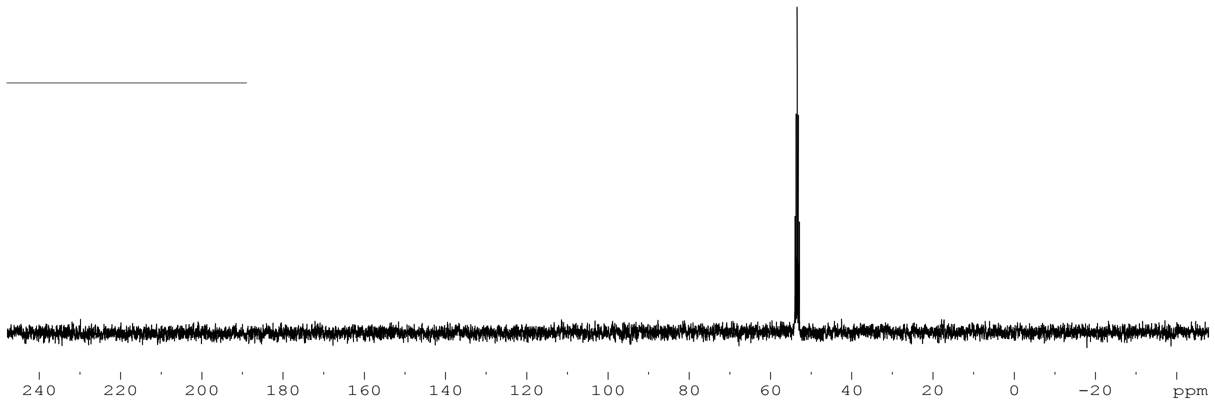

fig. S3c. ^{13}C NMR spectrum, thermally polarized, methanol doped, showing CD_2Cl_2 signal at 54 ppm.

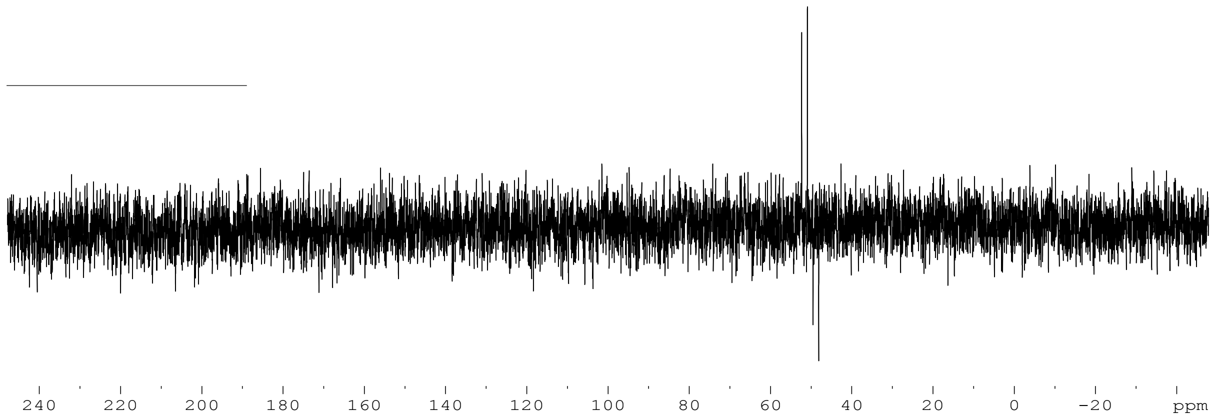

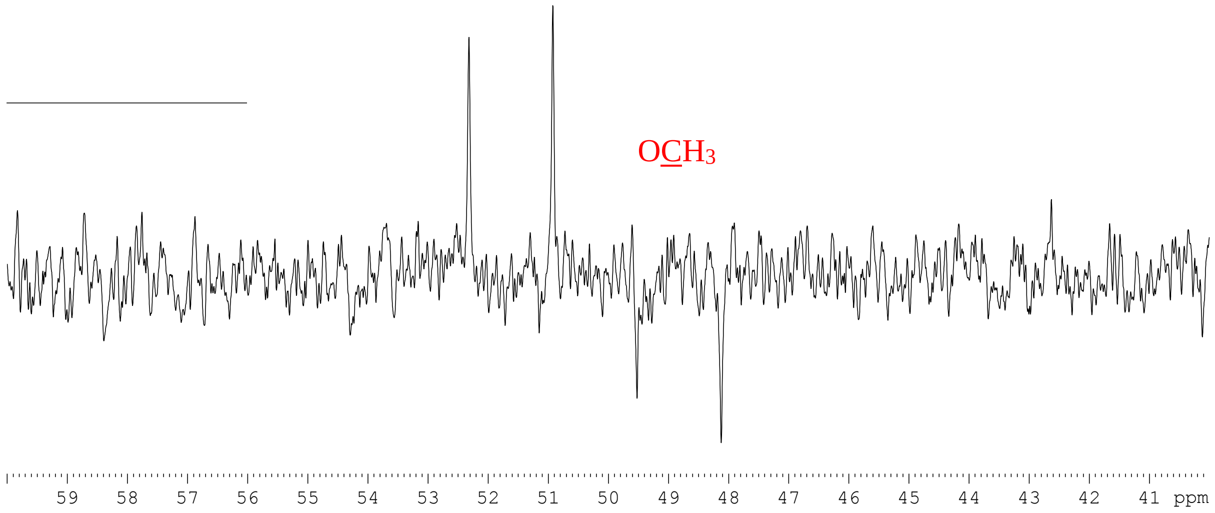

fig. S3d. ^{13}C SABRE-RELAY NMR spectrum for methanol CH_3 (top) and expansion (bottom).

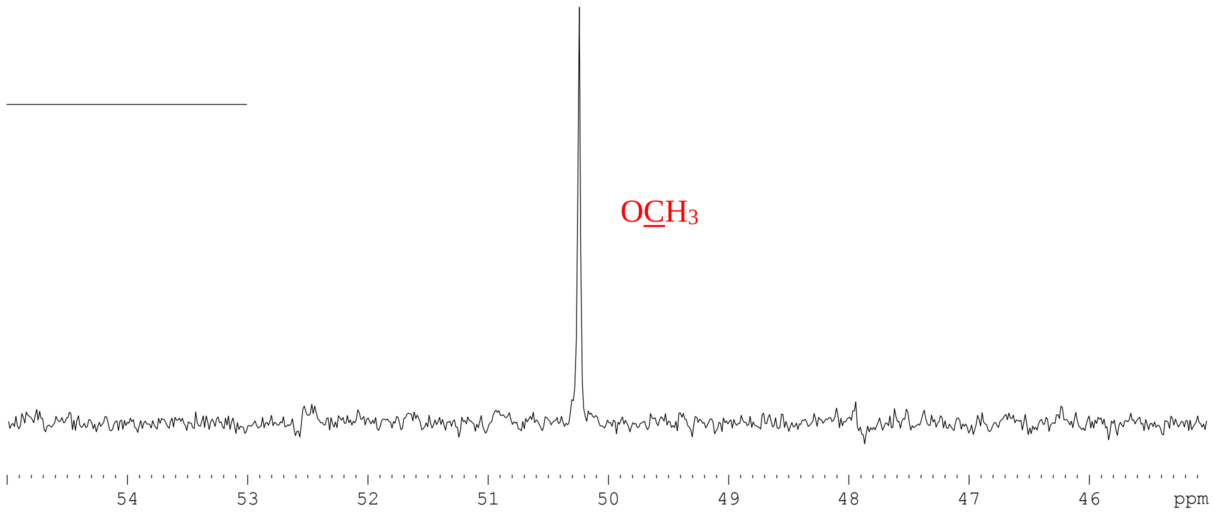

fig. S3e. ^{1}H-^{13}C SABRE-RELAY refocused INEPT NMR spectrum for methanol CH_3 signal. J_{XH} value used was 122.

Ethanol

fig. S4. SABRE-RELAY NMR spectra ethanol. (**a** to **e**) reflect a series of single scan NMR spectra: (a) ^{1}H NMR spectrum thermally polarized, (b) ^{1}H SABRE-RELAY NMR spectrum, (c) ^{13}C NMR spectrum thermally polarized , (d) ^{13}C SABRE-RELAY NMR spectrum, (e) ^{1}H-^{13}C refocused INEPT NMR spectrum after SABRE-RELAY and (**f**) ^{13}C DEPT NMR spectrum after SABRE RELAY. All NMR spectra are designed to show the hyperpolarized signals of added ethanol.

Conditions: [IrCl(COD)(IMes)] (5 mM), ethanol (1 uL.),CD_2Cl_2 (0.6 mL), NH_3; Substrate Concentration: 28.6 mM. ^{1}H signal enhancements (fold/proton): 242 (OH), 497 (-OCH_2-), 574 (-CH_3).

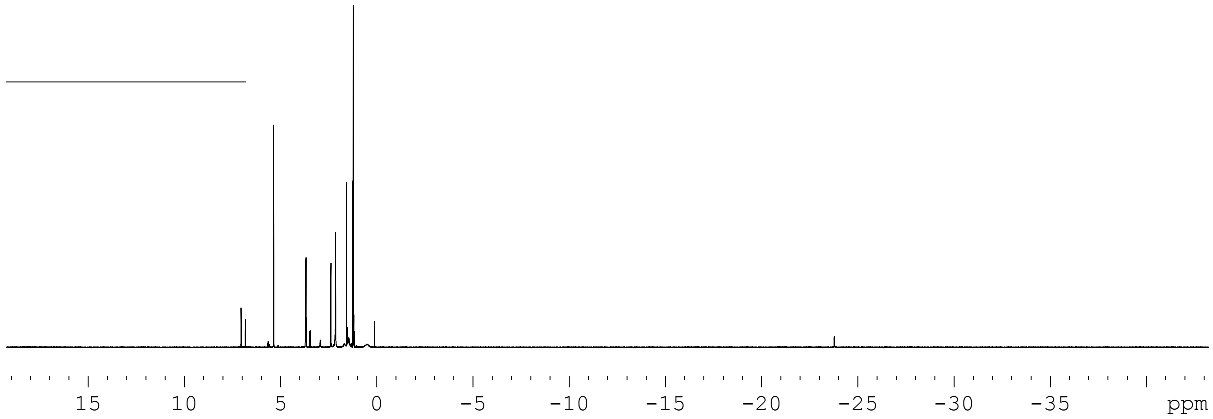

fig. S4a. Thermally polarized ^{1}H control NMR spectrum for ethanol sample.

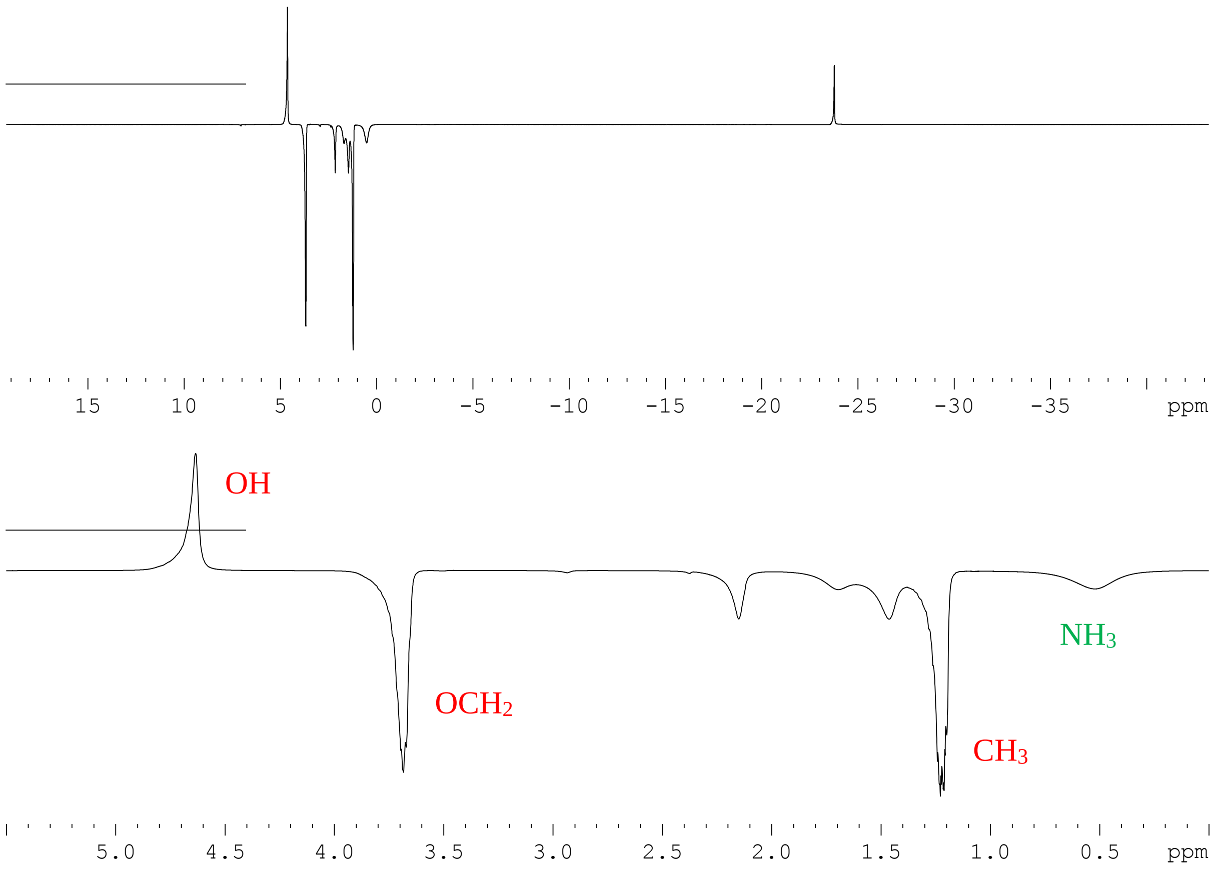

fig. S4b. ^{1}H SABRE-RELAY NMR spectrum for ethanol (top) and expansion (bottom).

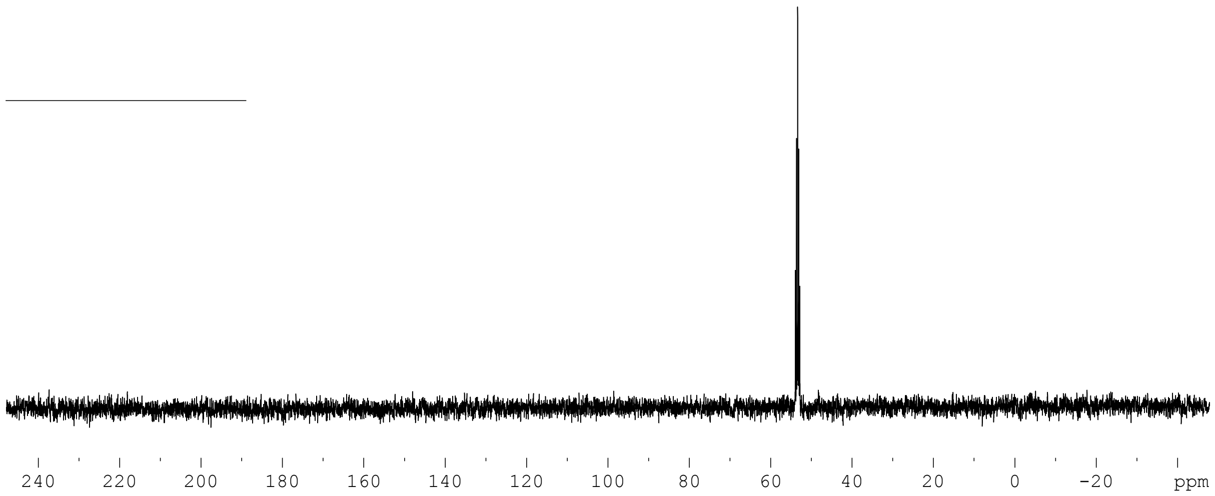

fig. S4c.Thermally polarized ^{13}C reference NMR spectrum for the ethanol sample showing the CD_2Cl_2 signal at 54 ppm.

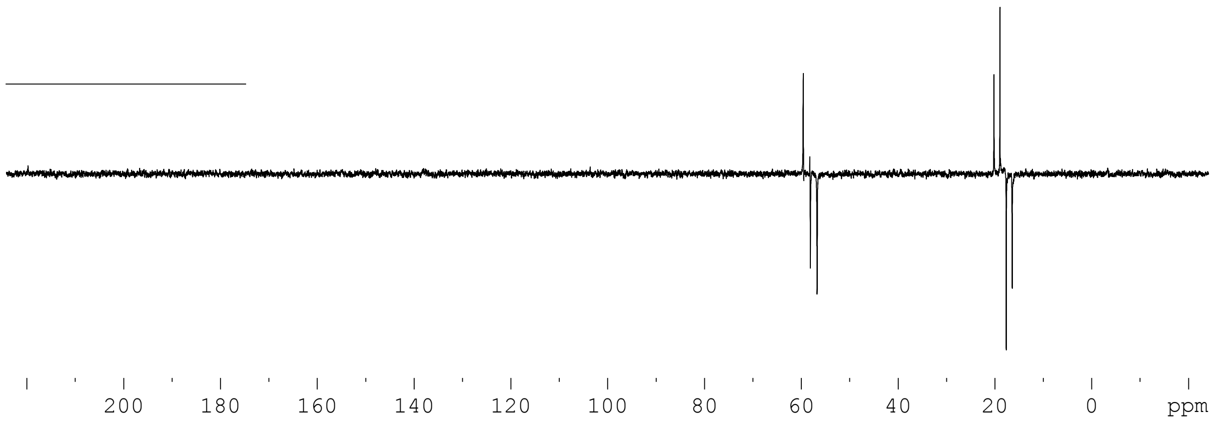

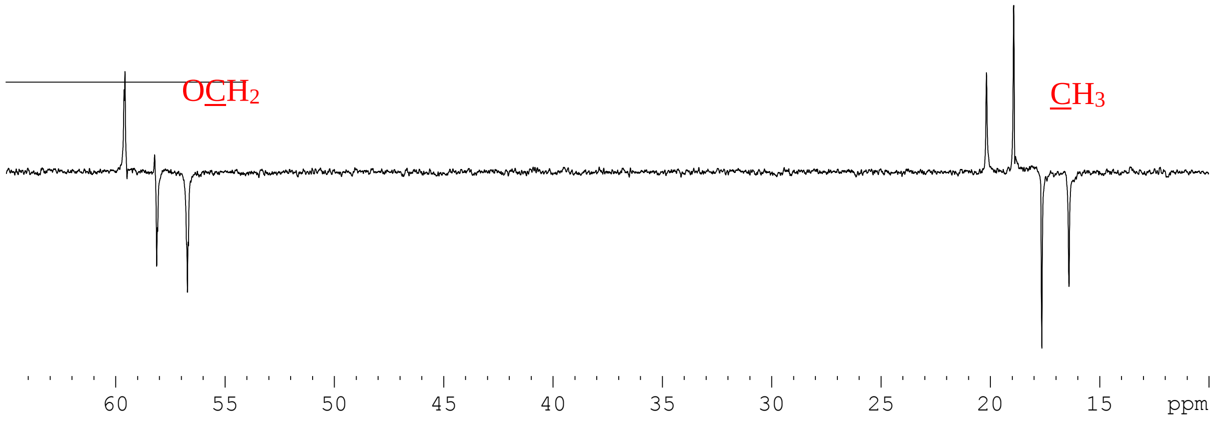

fig. S4d. ^{13}C SABRE-RELAY NMR spectrum for ethanol (top) and expansion (bottom).

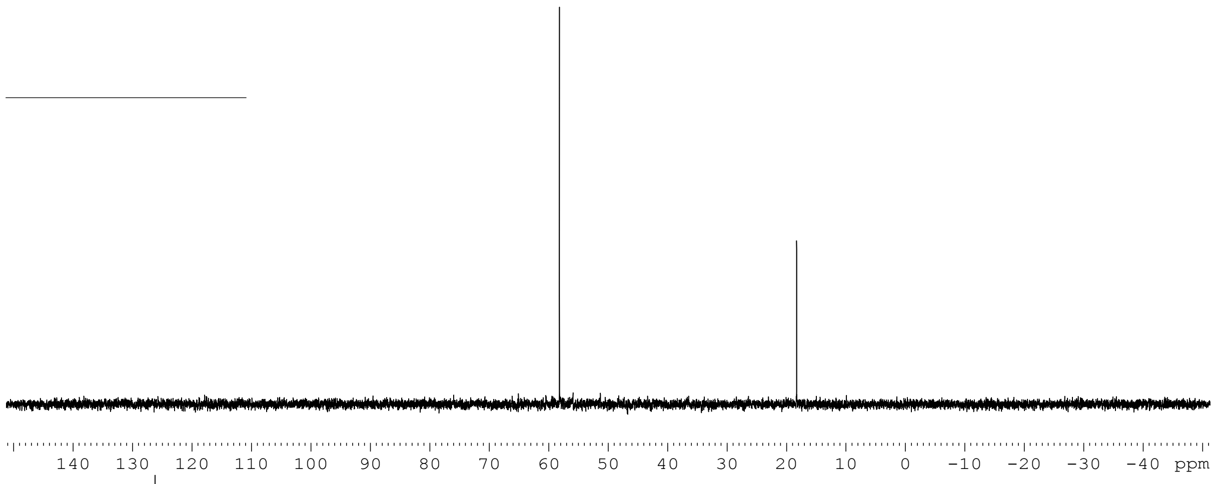

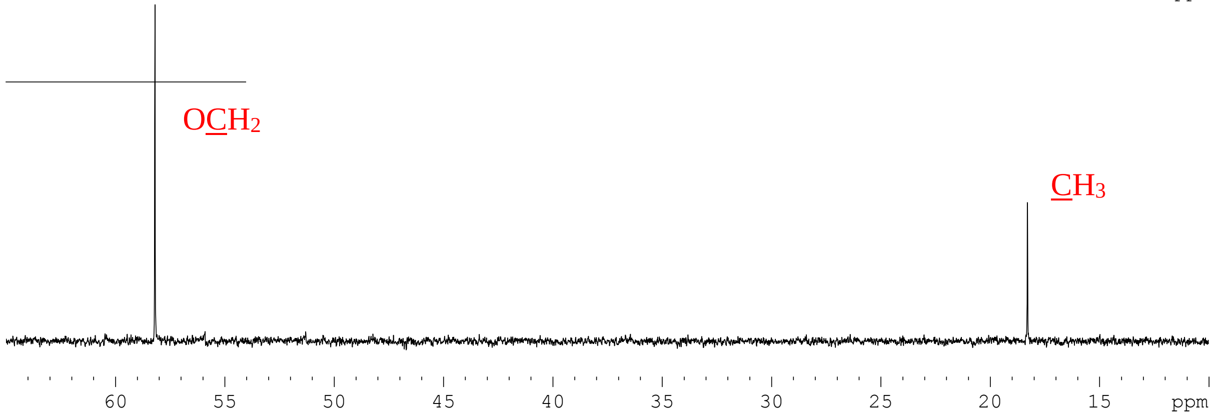

fig. S4e. ^{1}H-[13]CSABRE-RELAY refocused INEPT NMR spectrum for ethanol (top) and expansion (bottom). J_{XH} value used was 122.

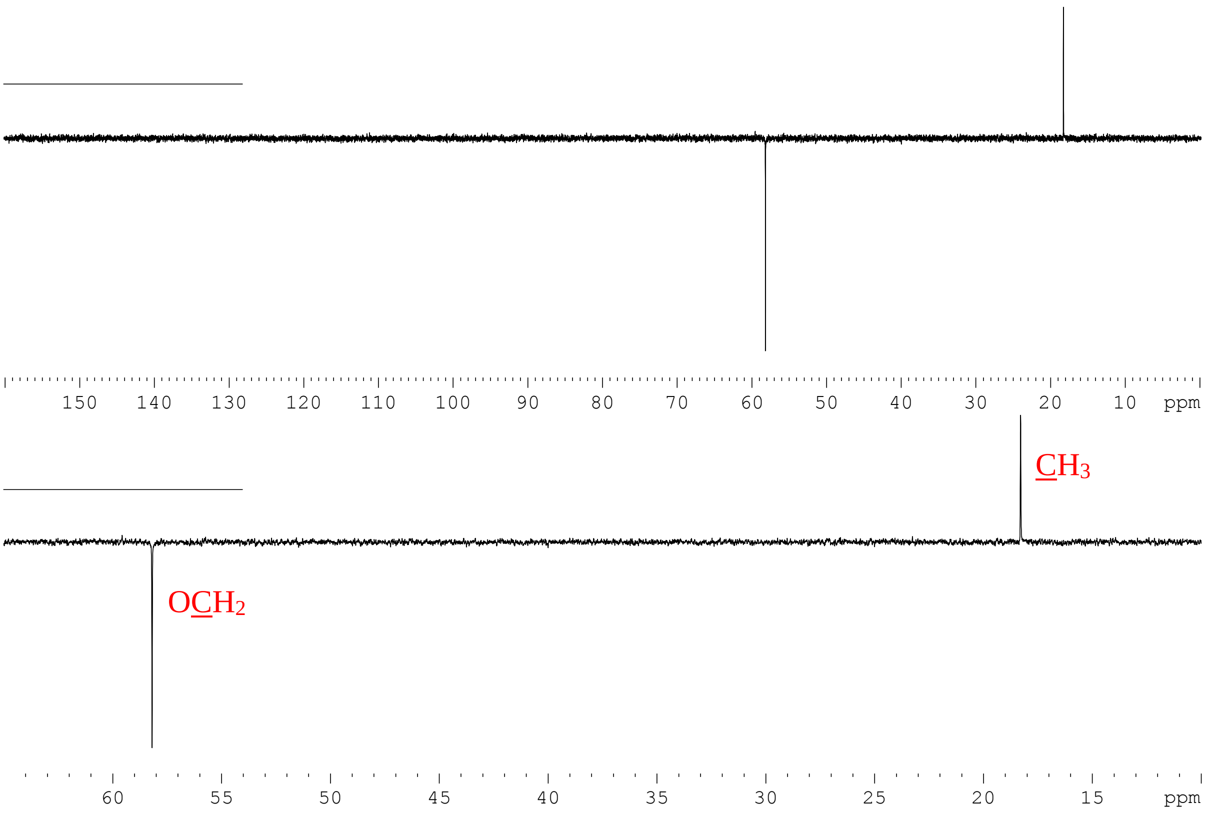

fig. S4f. ^{1}H-[13]CSABRE-RELAY DEPT NMR spectrum for ethanol (top) and expansion (bottom). J_{XH} value used was 122.

1-Propanol

fig. S5. SABRE-RELAY NMR spectra propanol. (**a** to **e**) reflect a series of single scan NMR spectra: (a) ^{1}H NMR spectrum thermally polarized, (b) ^{1}H SABRE-RELAY NMR spectrum, (c) ^{13}C NMR spectrum thermally polarized , (d) ^{13}C SABRE-RELAY NMR spectrum and (e) ^{1}H-^{13}C refocused INEPT NMR spectrum after SABRE-RELAY for 1-propanol using conditions: [IrCl(COD)(IMes)] (5 mM), 1-propanol (1 uL.),CD_2Cl_2 (0.6 mL), NH_3; Substrate Concentration: 22 mM. ^{1}H enhancement (fold/per proton): 223 (OH), 653 (-OCH_2-), 637 (-CH_2-), 539 (-CH_3).

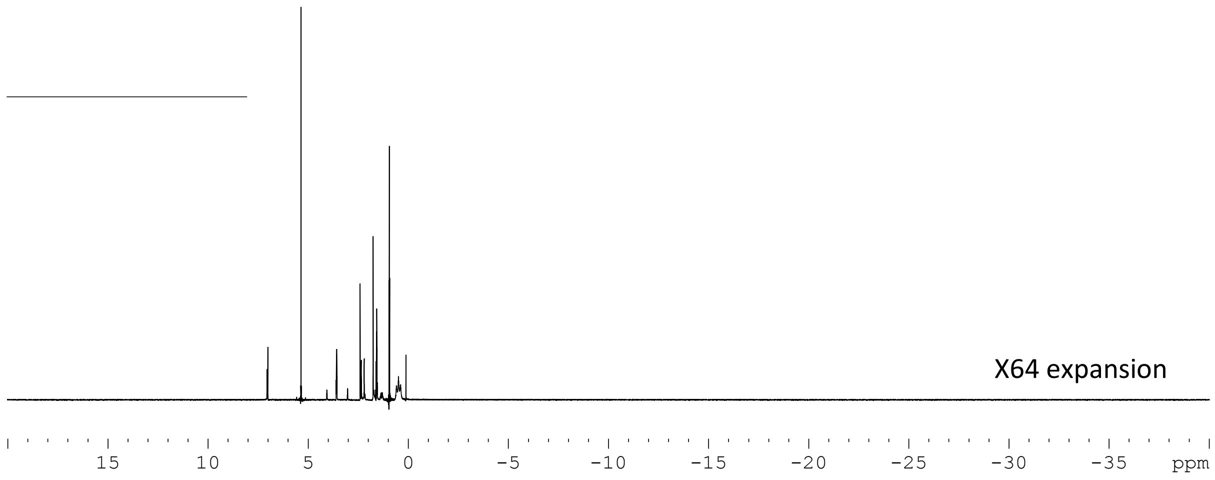

fig. S5a. Thermally polarized ^{1}H NMR spectrum for the 1-propanol doped sample to provide a reference point.

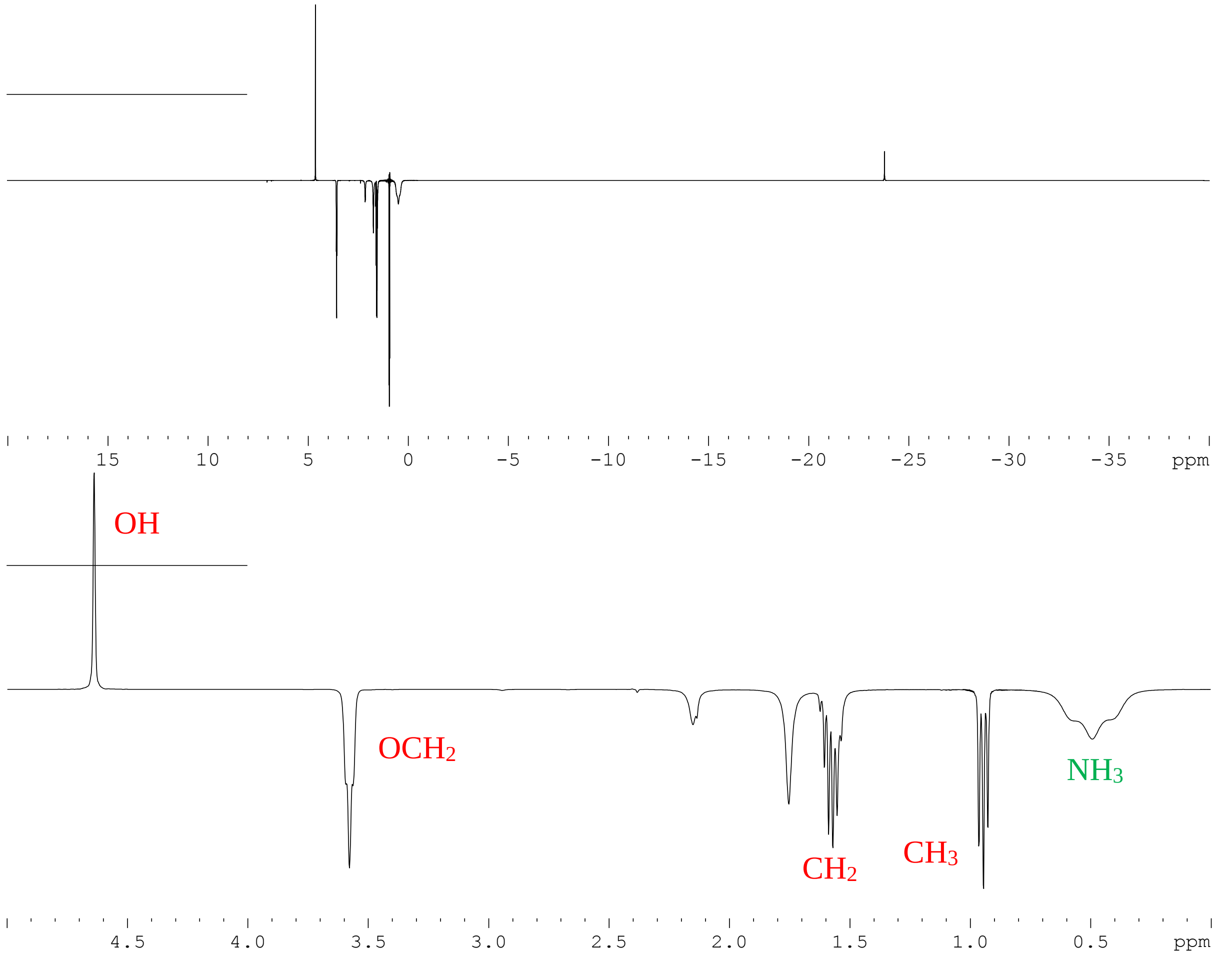

fig. S5b. ^{1}H SABRE-RELAY NMR spectrum for 1-propanol (top) and expansion (bottom).

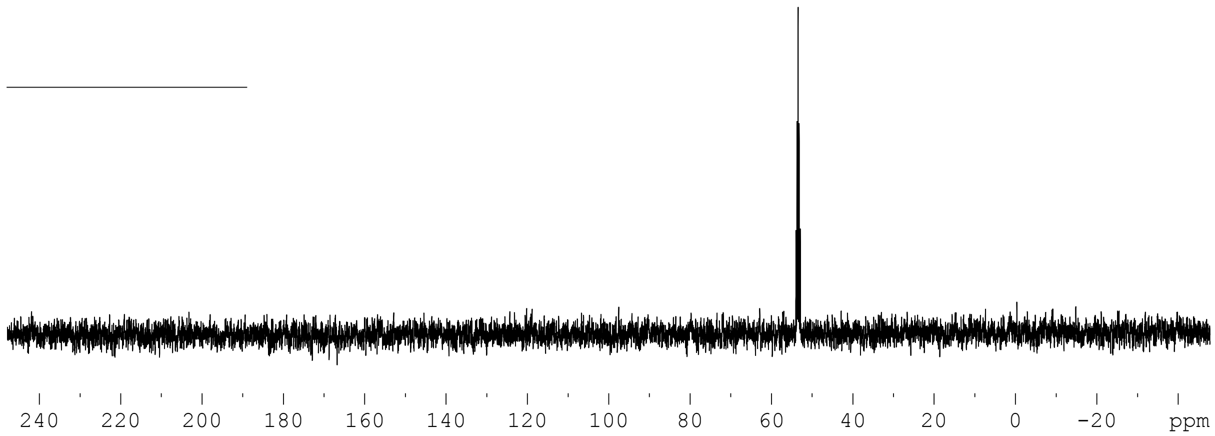

fig. S5c. Thermally polarized ^{13}C NMR spectrum for 1-propanol showing the CD_2Cl_2 signal at 54 ppm.

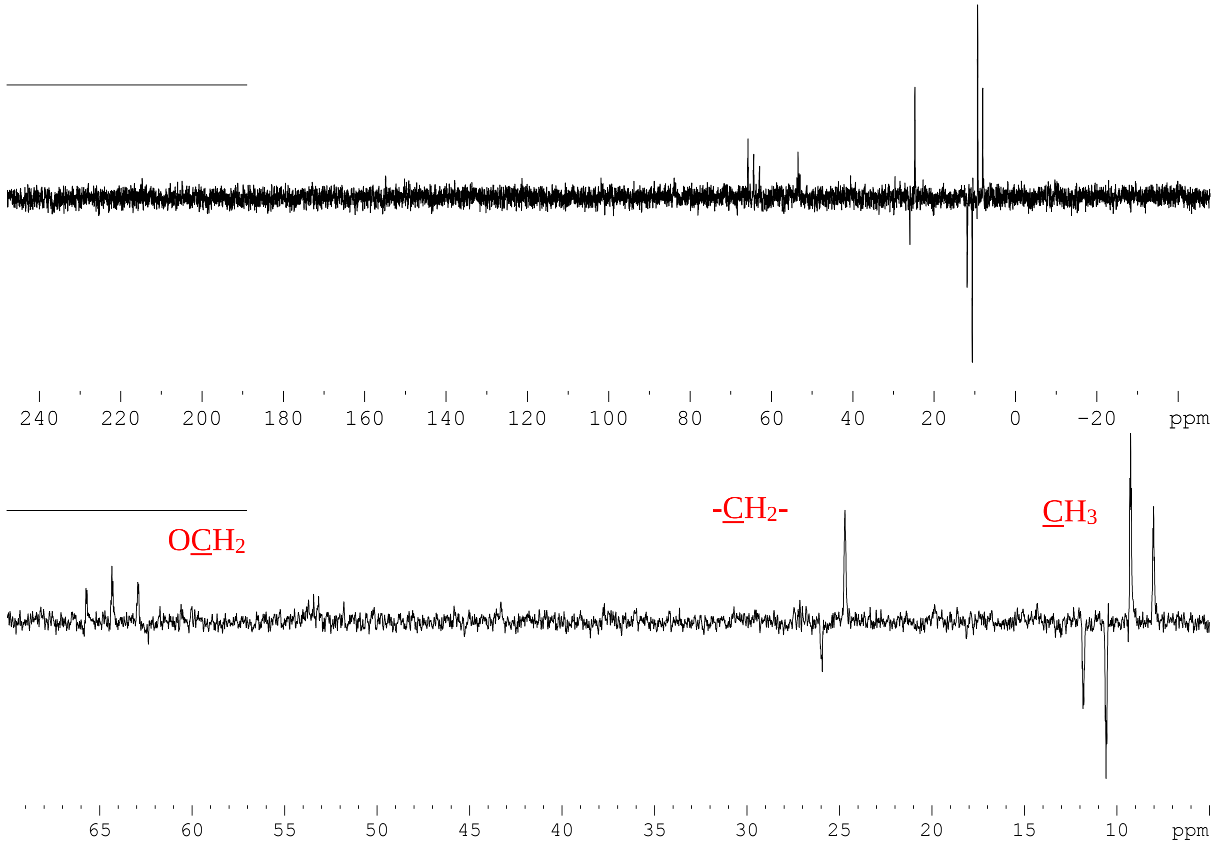

fig. S5d. ^{13}C SABRE-RELAY NMR spectrum for 1-propanol (top) and expansion (bottom).

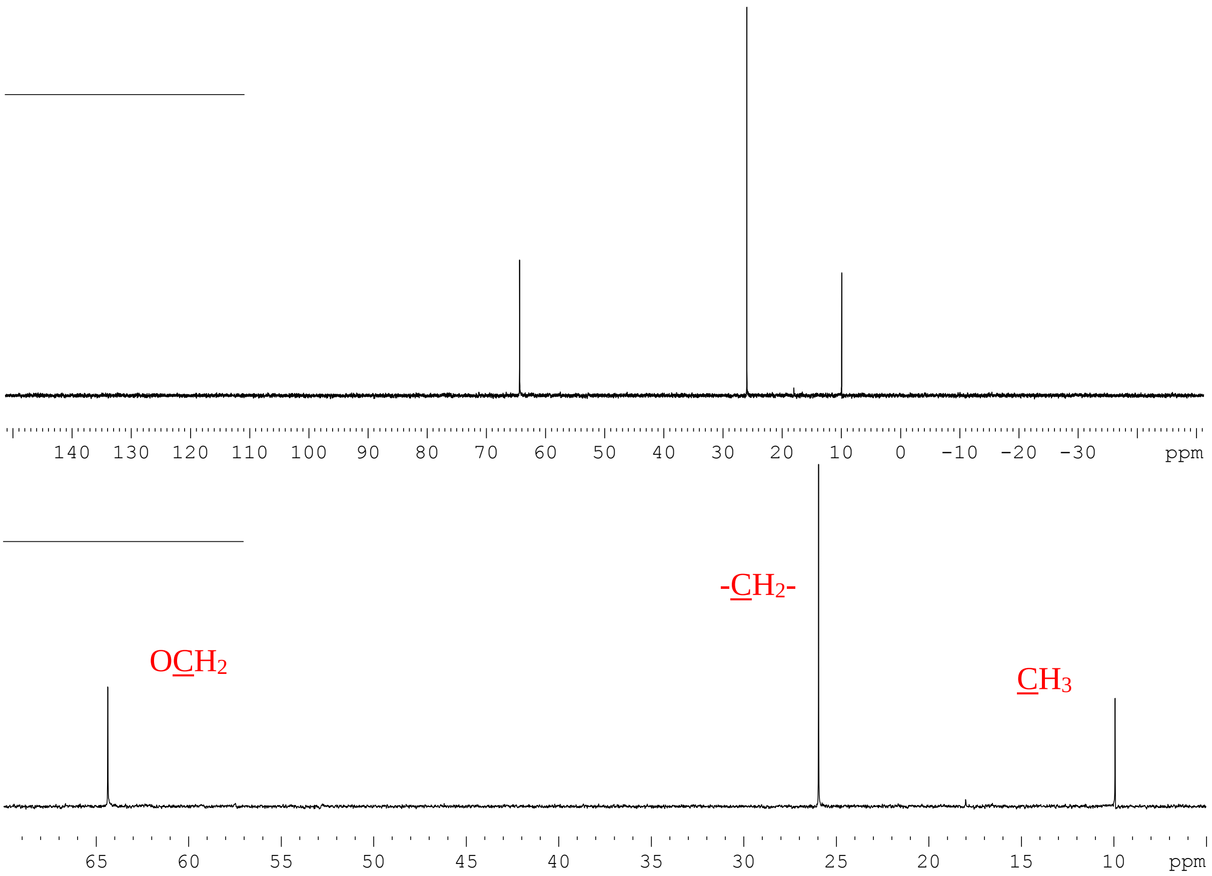

fig. S5e. ^{1}H-^{13}CSABRE-RELAY-refocused INEPT NMR spectrum for 1-propanol (top) and expansion (bottom). J_{XH} value used was 122.

fig. S6. SABRE-RELAY NMR spectra propanol, low concentration. (**a** to **d**) reflect a series of single scan NMR spectra at reduced 1-propanol concentration: (a) ^{1}H NMR spectrum thermally polarized, (b) ^{1}H SABRE-RELAY NMR spectrum, (c) ^{13}C NMR spectrum thermally polarized , (d) ^{1}H-^{13}C refocused INEPT NMR spectrum after SABRE-RELAY for 1-propanol using conditions: [IrCl(COD)(IMes)] (5 mM), 1-propanol (0.1 uL.),CD_2Cl_2 (0.6 mL), NH_3; Substrate Concentration: 2.2 mM. ^{1}H enhancement (fold/per proton): 528 (OH), 790 (-OCH_2-), peak overlap (-CH_2-), 773 (-CH_3).

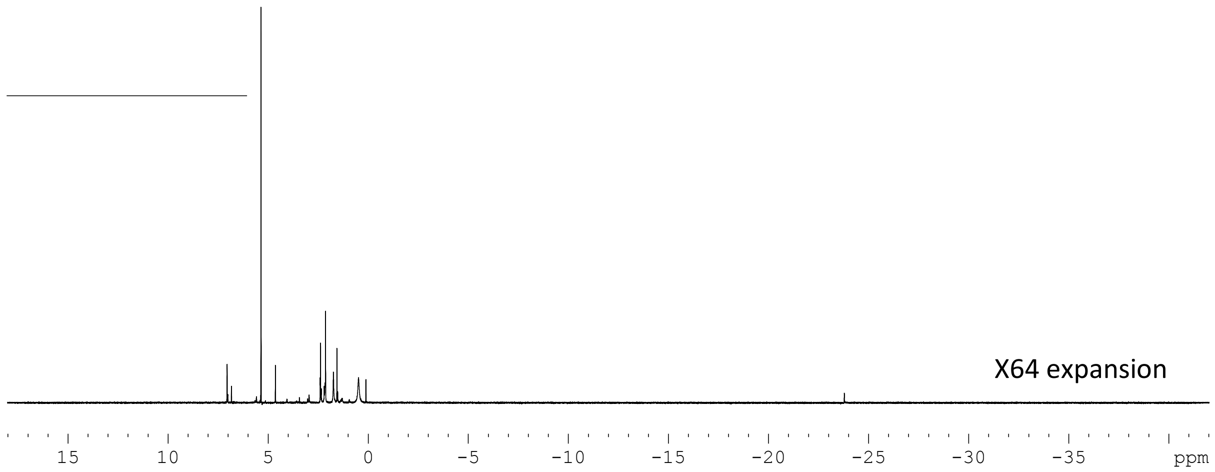

fig. S6a. Thermally polarized ^{1}H NMR spectrum for 1-propanol at lower concentration.

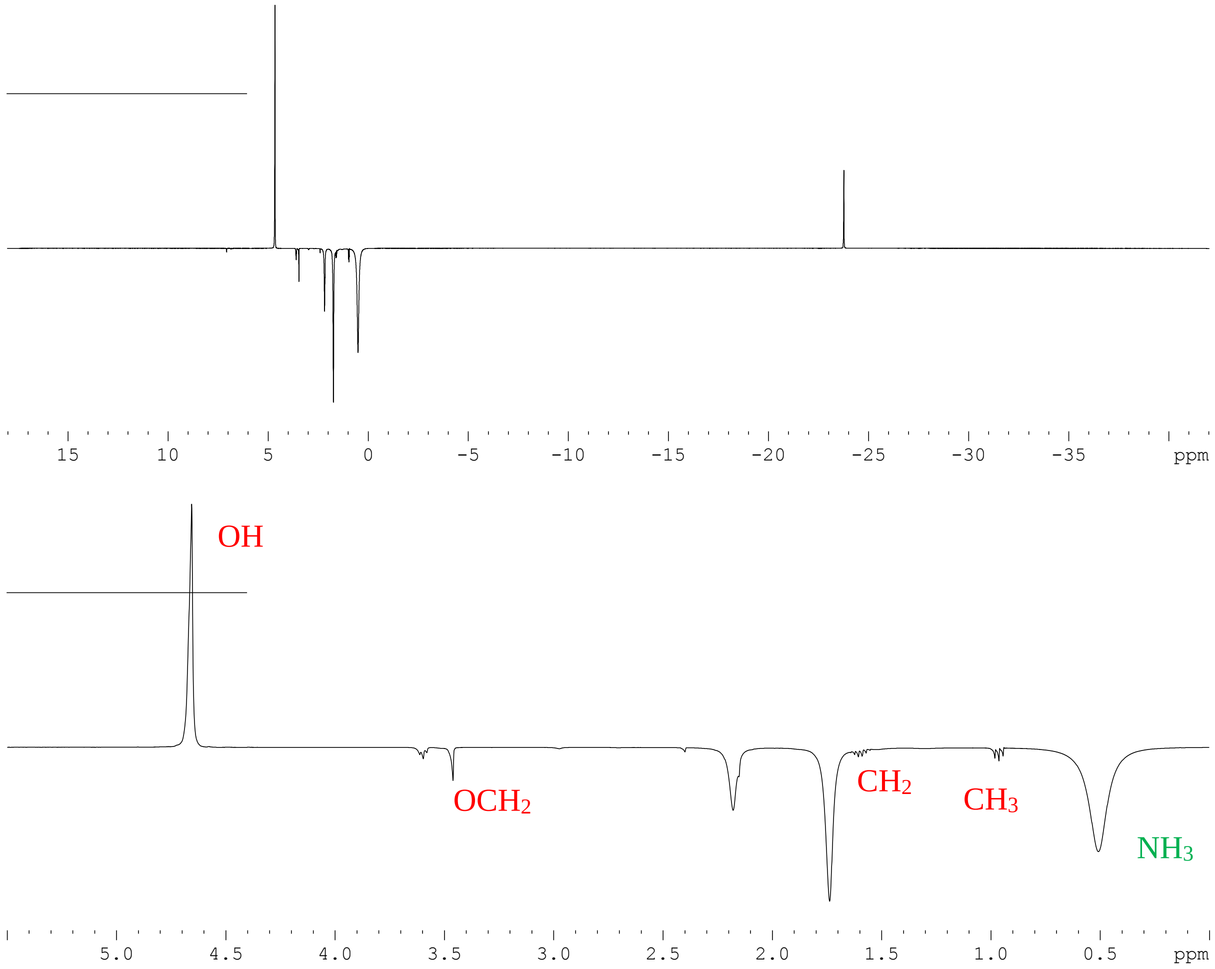

fig. S6b. ^{1}H SABRE-RELAY NMR spectrum for 1-propanol at low concentration (top) and expansion (bottom).

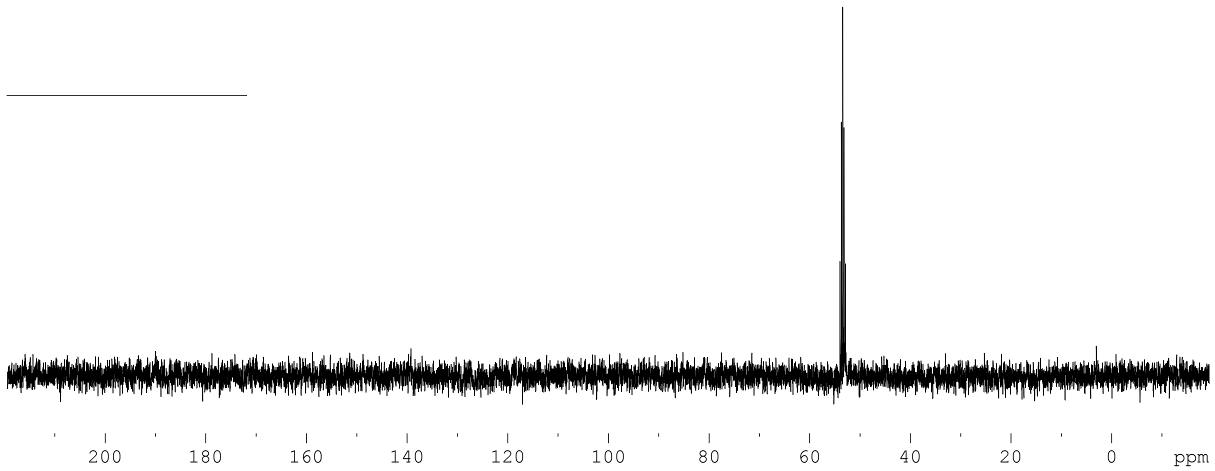

fig. S6c. ^{13}C Thermally polarized polarized NMR spectrum for 1-propanol at lower concentration showing the CD_2Cl_2 signal at 54 ppm.

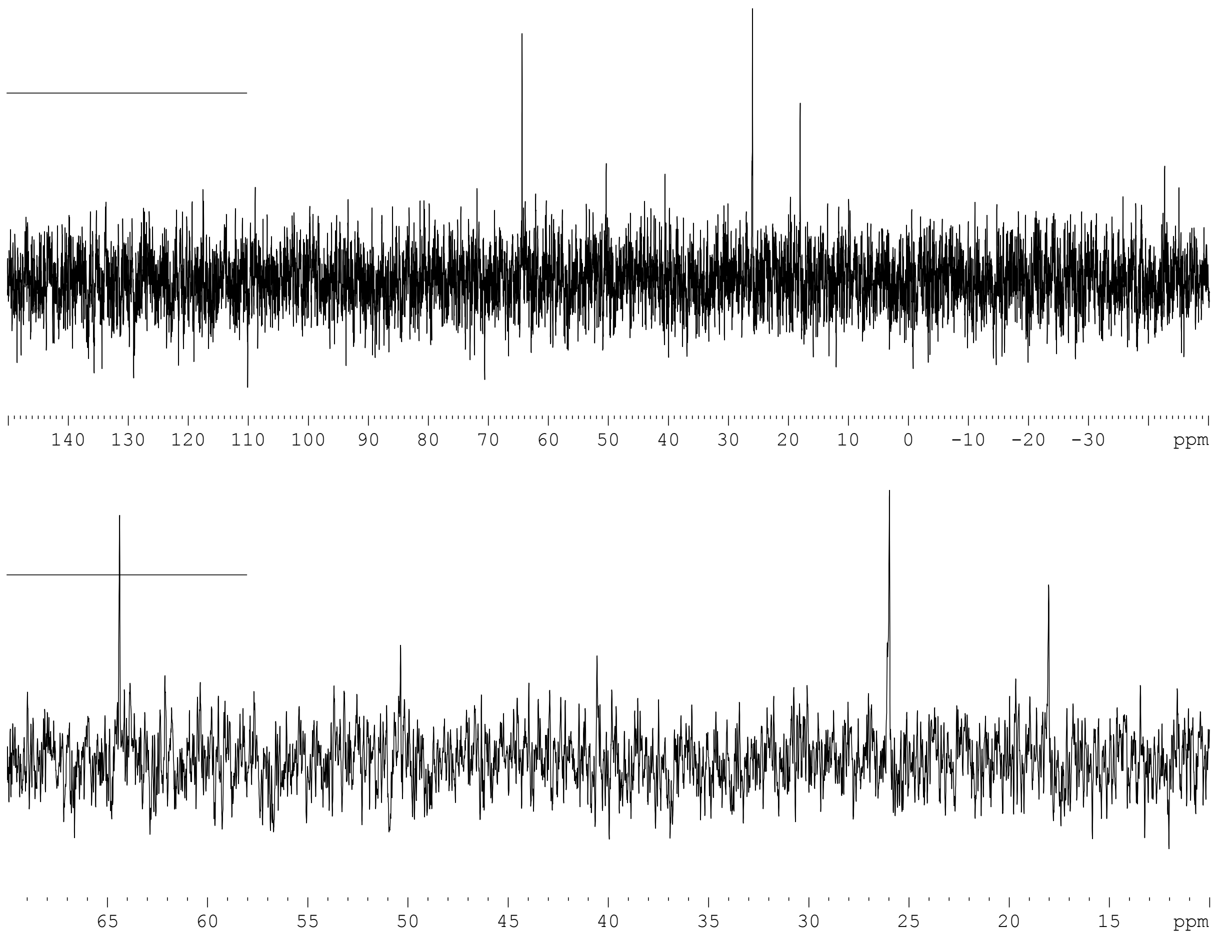

fig. S6d. ^{1}H-^{13}CSABRE-RELAY-refocused INEPT NMR spectrum for 1-propanol at low concentration (top) and expansion (bottom). J_{XH} value used was 122.

1-Butanol

fig. S7. SABRE-RELAY NMR spectra butanol. (**a** to **d**) reflect a series of single scan NMR spectra: (a) ^{1}H NMR spectrum thermally polarized (x32 vertical expansion), b) ^{1}H SABRE-RELAY NMR spectrum, (c) ^{13}C thermally polarized NMR spectrum, (d) ^{13}C SABRE RELAY NMR spectrumand (**e**) ^{1}H-^{13}C refocused INEPT NMR spectrum after SABRE-RELAY for 1-butanol using conditions: [IrCl(COD)(IMes)] (5 mM), 1-butanol l (1 uL.), CD_2Cl_2 (0.6 mL), NH_3; Substrate Concentration: 18.5 mM. ^{1}H enhancement (fold/per proton): 474 (OH), 494 (-OCH_2-), 396 (-$[CH_2]_2$-), 444 (-CH_3).

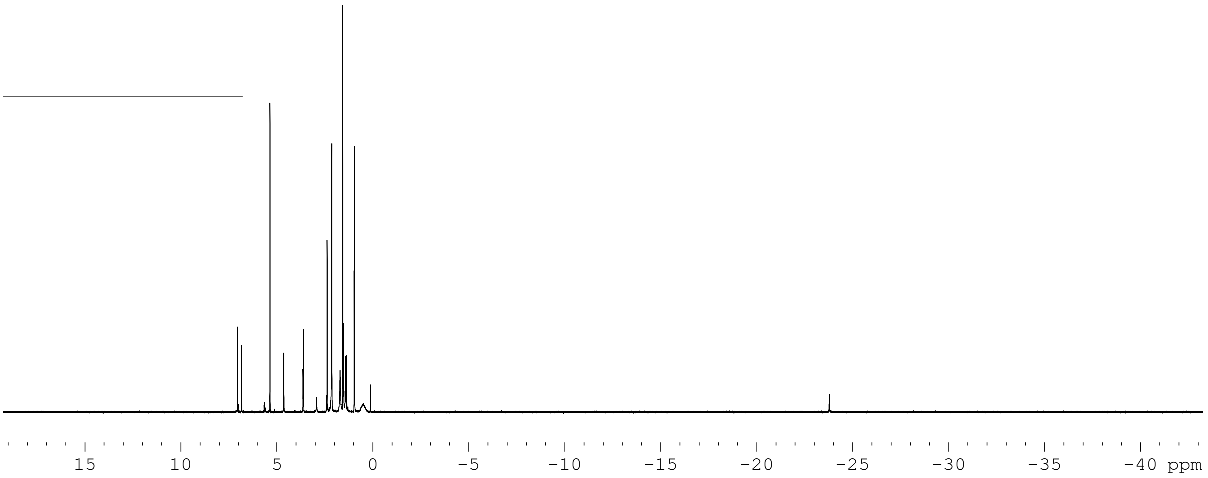

fig. S7a. Thermally polarized ^{1}H NMR reference spectrum for 1-butanol.

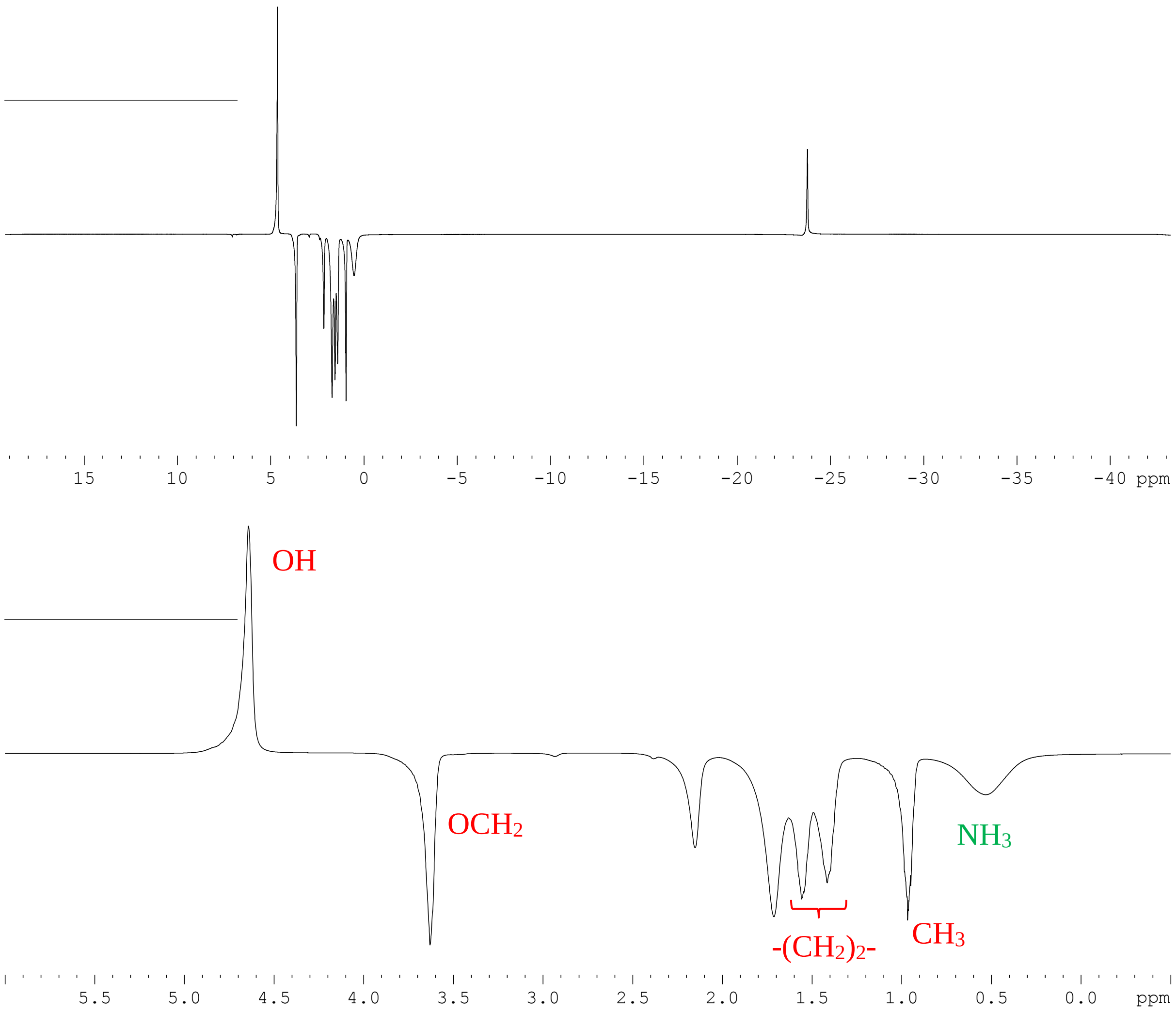

fig. S7b. ^{1}H SABRE-RELAY NMR spectrum for 1-butanol (top) and expansion (bottom).

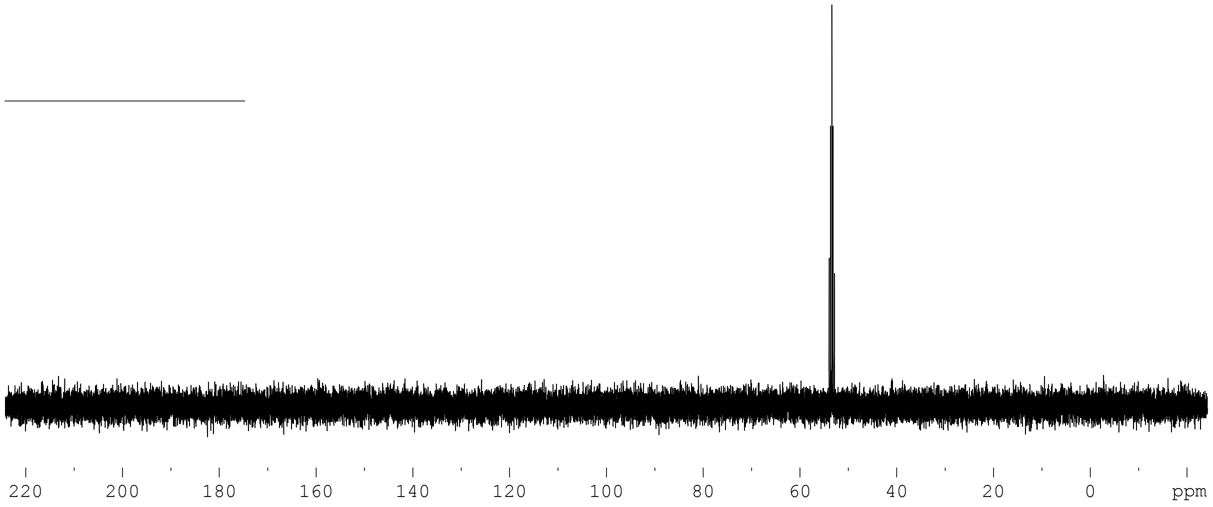

fig. S7c. ^{13}C thermally polarized control spectrum for 1-butanol showing the CD_2Cl_2 signal at 54 ppm.

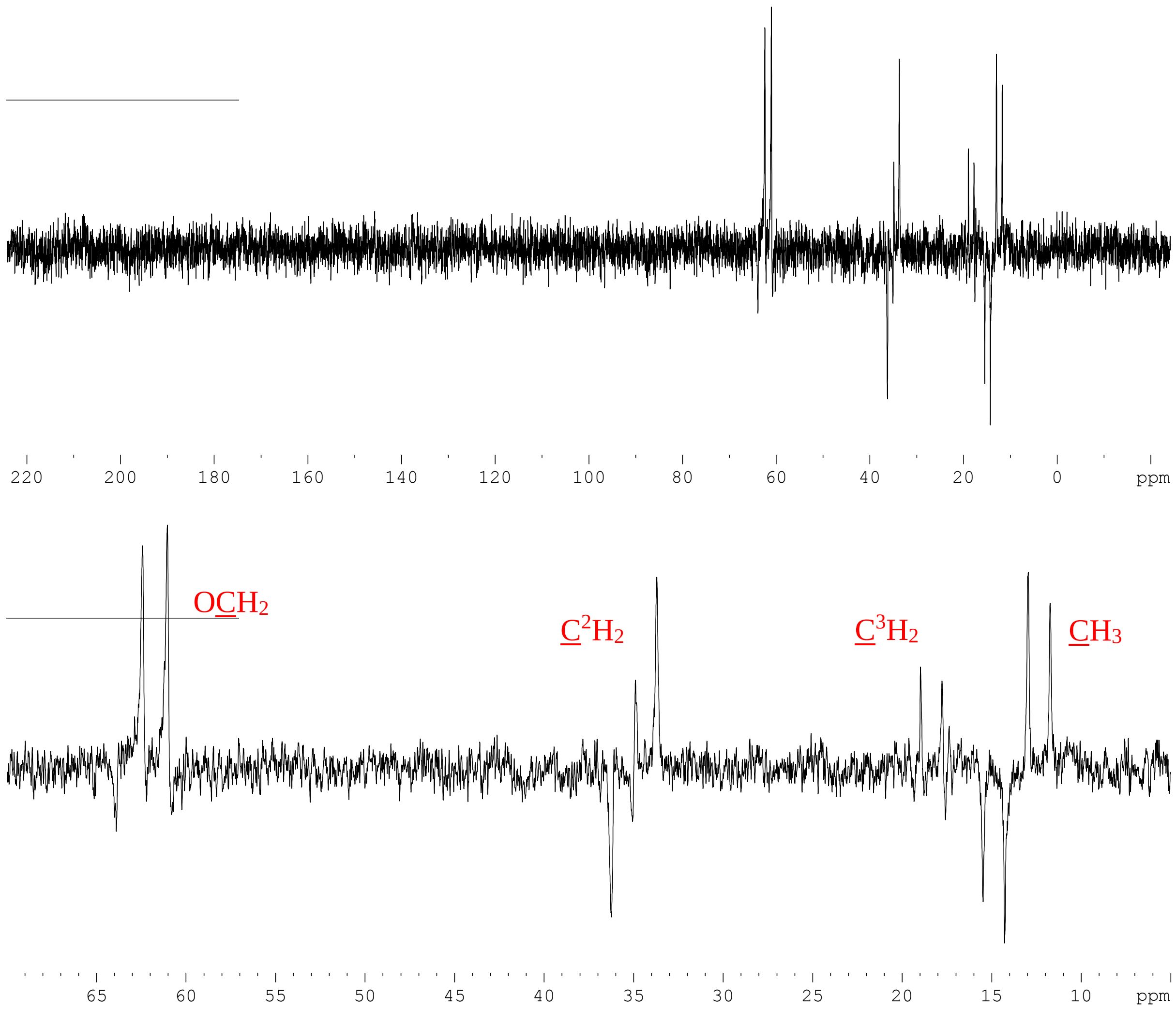

fig. S7d. ^{13}C hyperpolarized NMR spectrum for 1-butanol (top) and expansion (bottom).

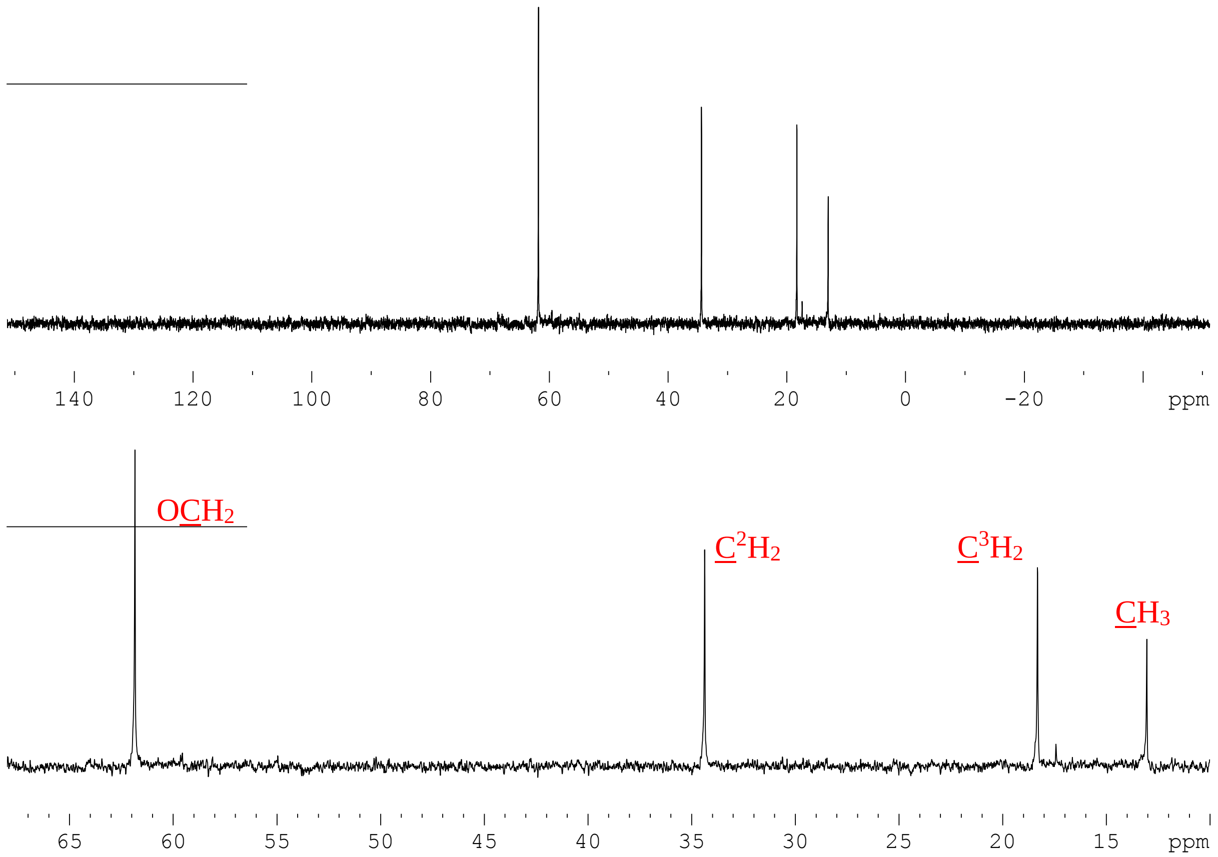

fig. S7e. ^{1}H-^{13}C SABRE-RELAY-refocused INEPT NMR spectrum for 1-butanol (top) and expansion (bottom). J_{XH} value used was 122.

1-Pentanol

fig. S8. SABRE-RELAY NMR spectra pentanol. (**a** to **e**) reflect a series of single scan NMR spectra: (a) ^{1}H NMR spectrum thermally polarized, (b) ^{1}H SABRE-RELAY NMR spectrum, (c) ^{13}C NMR spectrum thermally polarized , (d) ^{13}C SABRE-RELAY NMR spectrum and (e) ^{1}H-^{13}C refocused INEPT NMR spectrum after SABRE-RELAY of 1-pentanol using conditions: [IrCl(COD)(IMes)] (5 mM), 1-pentanol (1 uL.), CD_2Cl_2 (0.6 mL), NH_3; Substrate Concentration: 15.3 mM. ^{1}H enhancement: 134 (OH), 296 (-OCH_2-), 143 (-$[CH_2]_3$-), 209 (-CH_3).

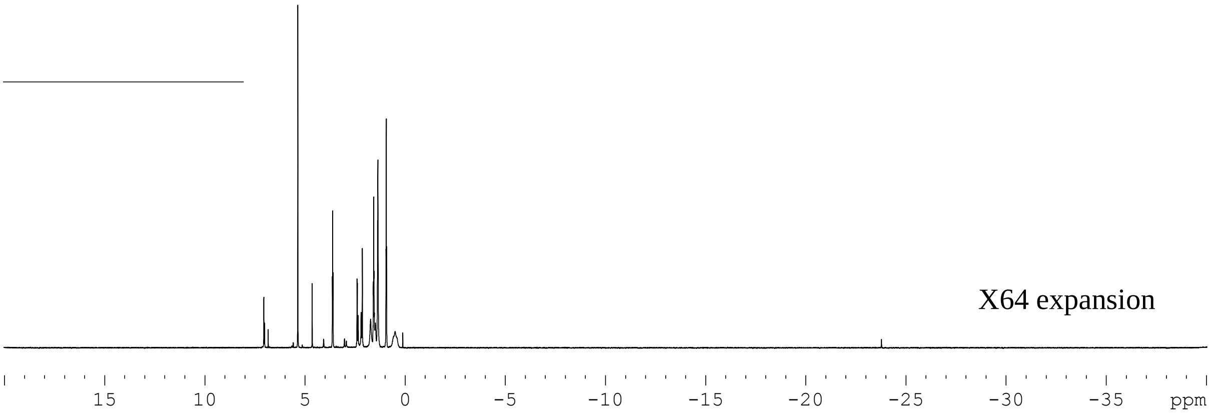

fig. S8a. Thermally polarized ^{1}H NMR reference spectrum for 1-pentanol.

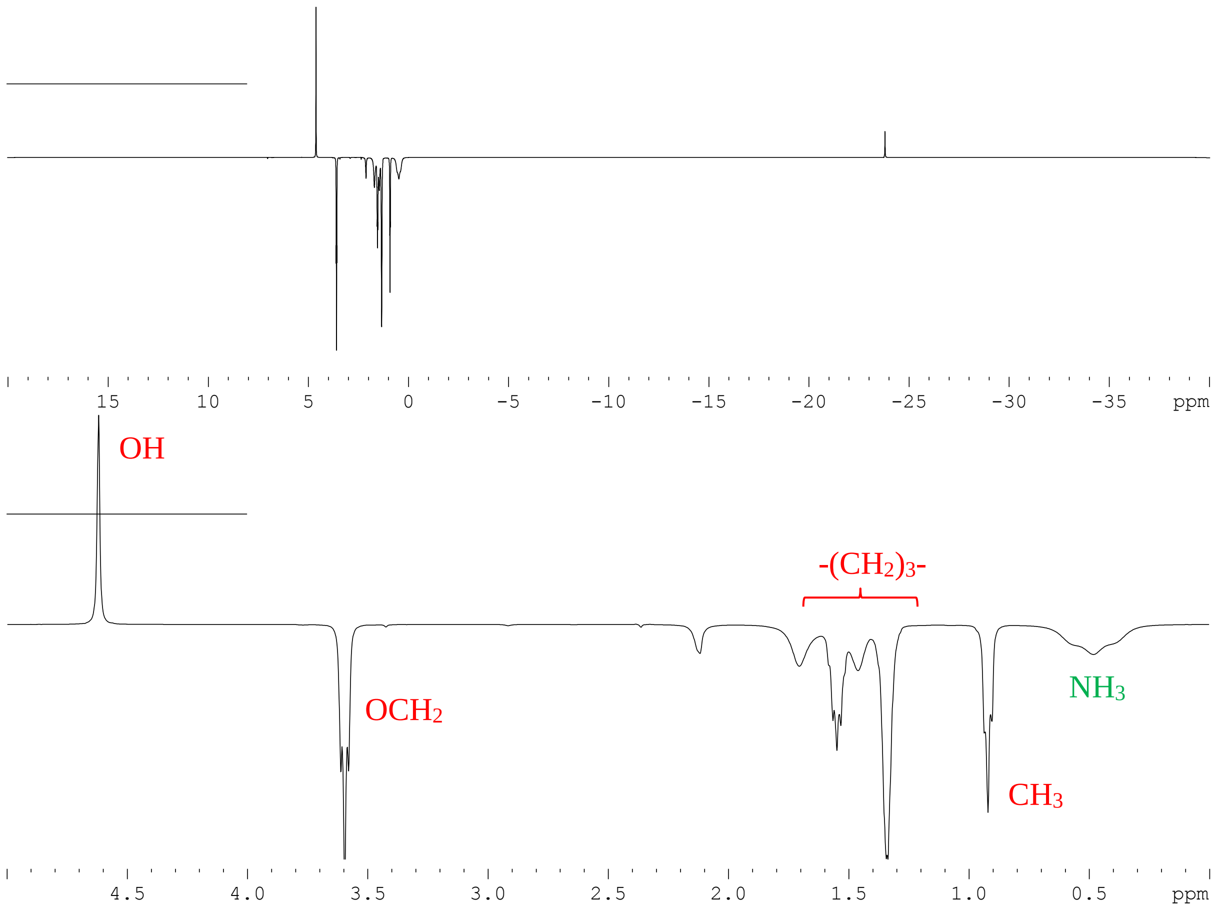

fig. S8b. ^{1}H SABRE-RELAY NMR spectrum for 1-pentanol (top) and expansion (bottom).

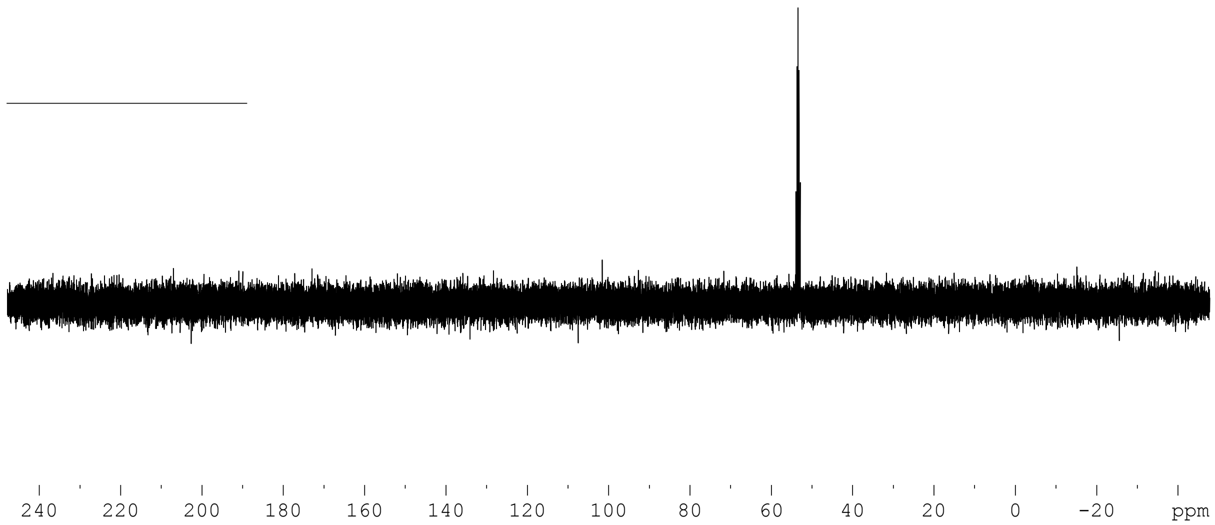

fig. S8c. ^{13}C thermally polarized control NMR spectrum for 1-pentanol showing the CD_2Cl_2 signal at 54 ppm.

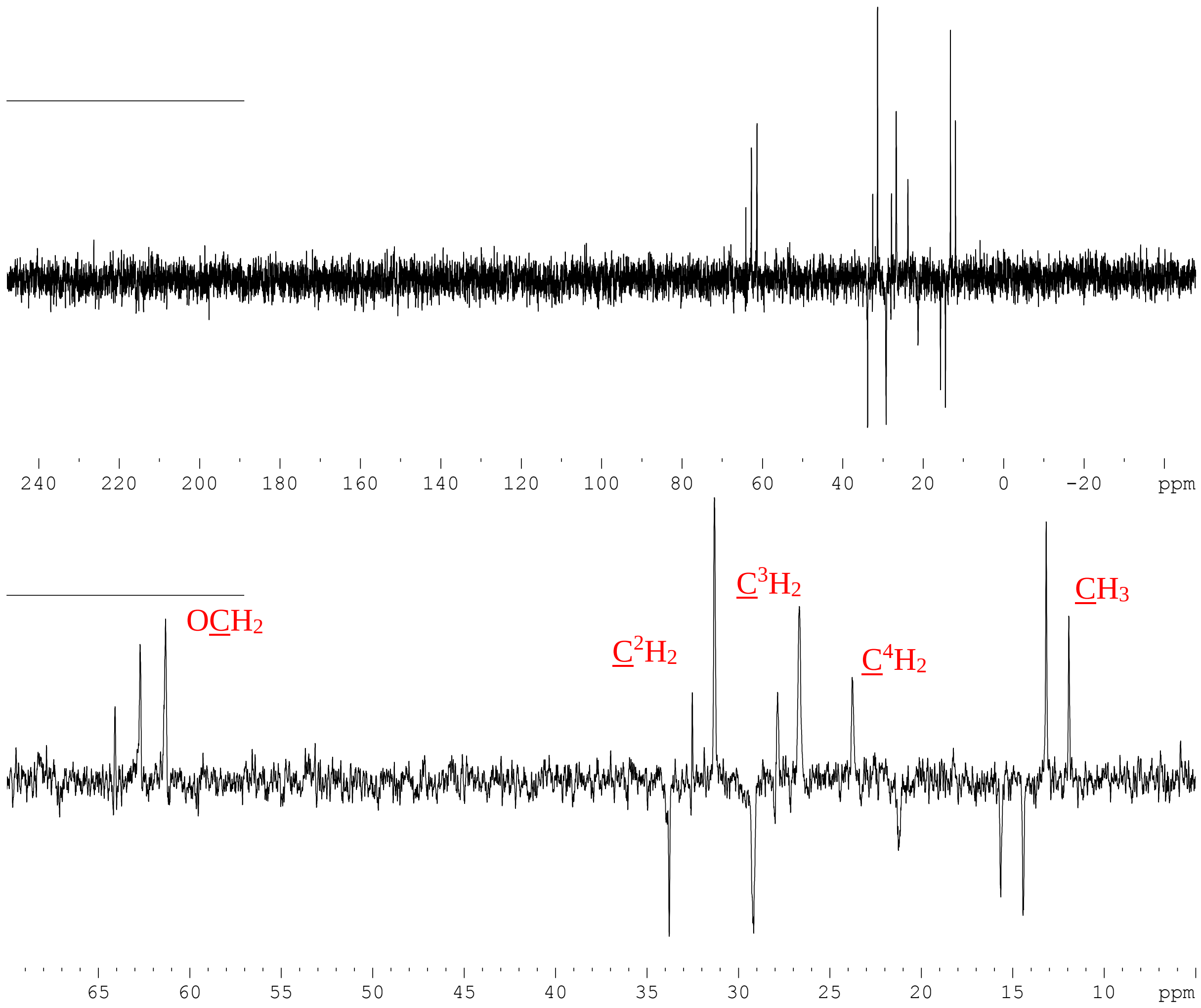

fig. S8d. ^{13}C SABRE-RELAY NMR spectrum for 1-pentanol (top) and expansion (bottom).

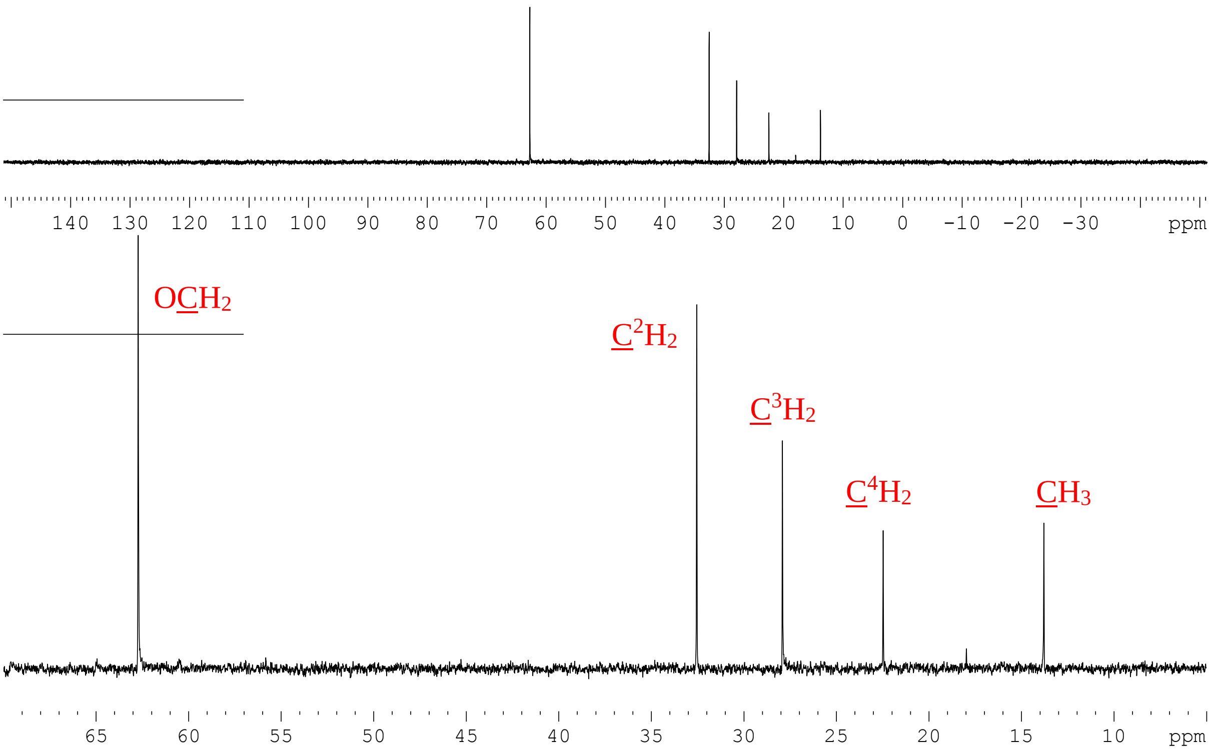

fig. S8e. ^{1}H-^{13}CSABRE-RELAY-refocused INEPT NMR spectrum for 1-pentanol (top) and expansion (bottom). J_{XH} value used was 122.

1-Hexanol

fig. S9. SABRE-RELAY NMR spectra hexanol. (**a** to **e**) reflect a series of single scan NMR spectra: (a) ^{1}H NMR spectrum thermally polarized, (b) ^{1}H SABRE-RELAY NMR spectrum, (c) ^{13}C NMR spectrum thermally polarized , (d) ^{13}C SABRE-RELAY NMR spectrum and (e) ^{1}H-^{13}C refocused INEPT NMR spectrum after SABRE-RELAY for 1-hexanol using conditions: [IrCl(COD)(IMes)] (5 mM), 1-hexanol (1 uL.), CD_2Cl_2 (0.6 mL), NH_3; Substrate Concentration: 13.2 mM. ^{1}H enhancement (fold/per proton): 179 (OH), 327 (-OCH_2-), 149 (-$[CH_2]_4$-), 212 (-CH_3).

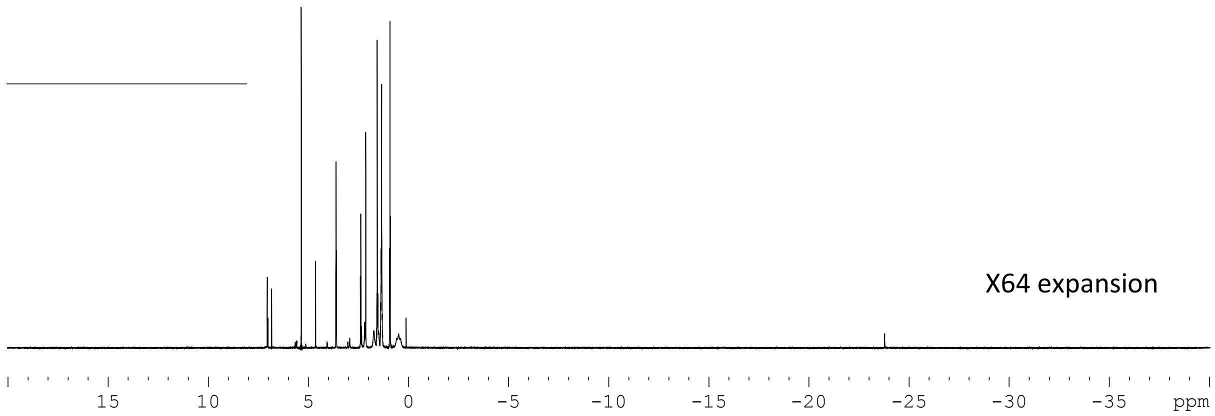

fig. S9a. Thermally polarized ^{1}H NMR reference spectrum for 1-hexanol.

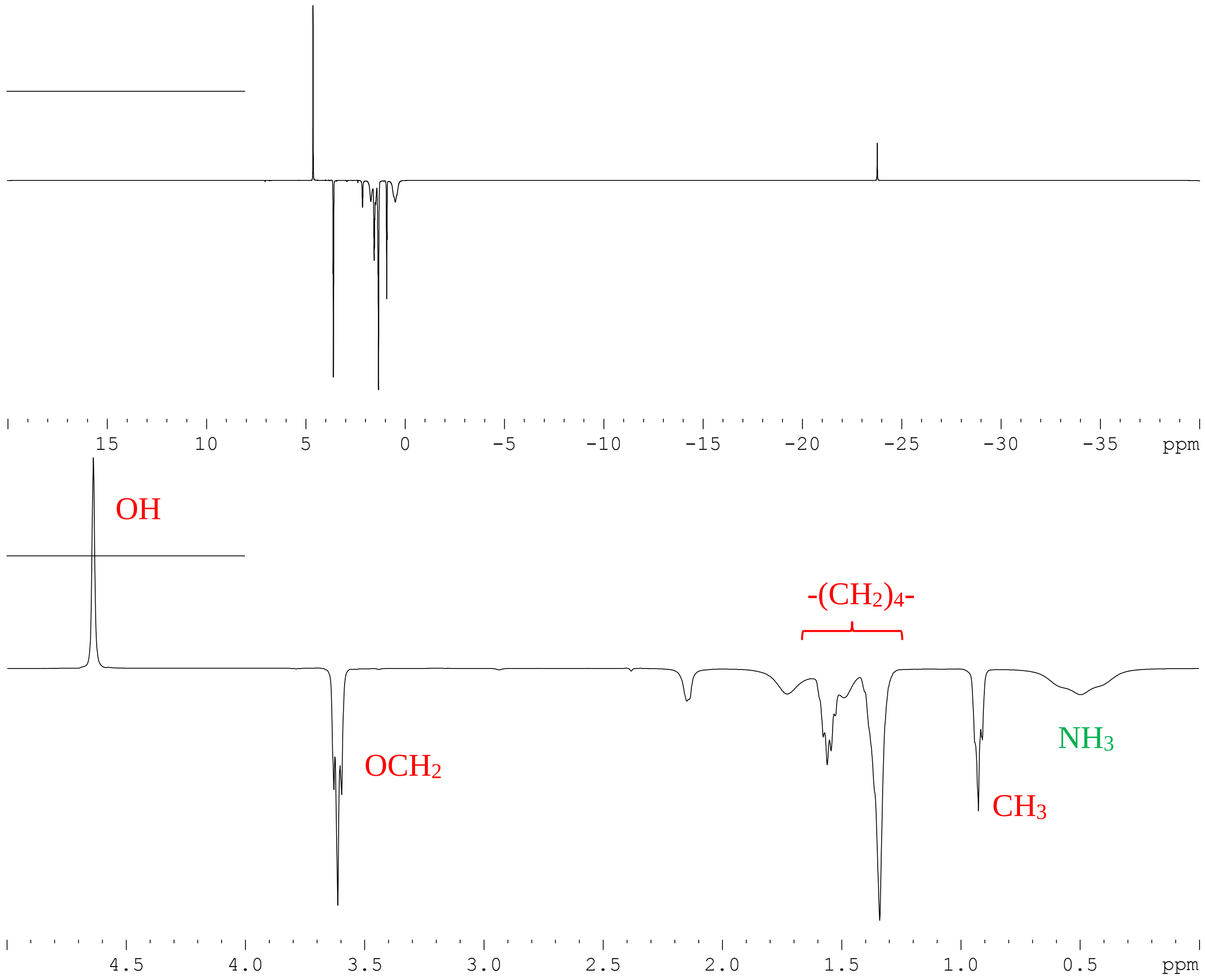

fig. S9b. ^{1}H SABRE-RELAY NMR spectrum for 1-hexanol (top) and expansion (bottom).

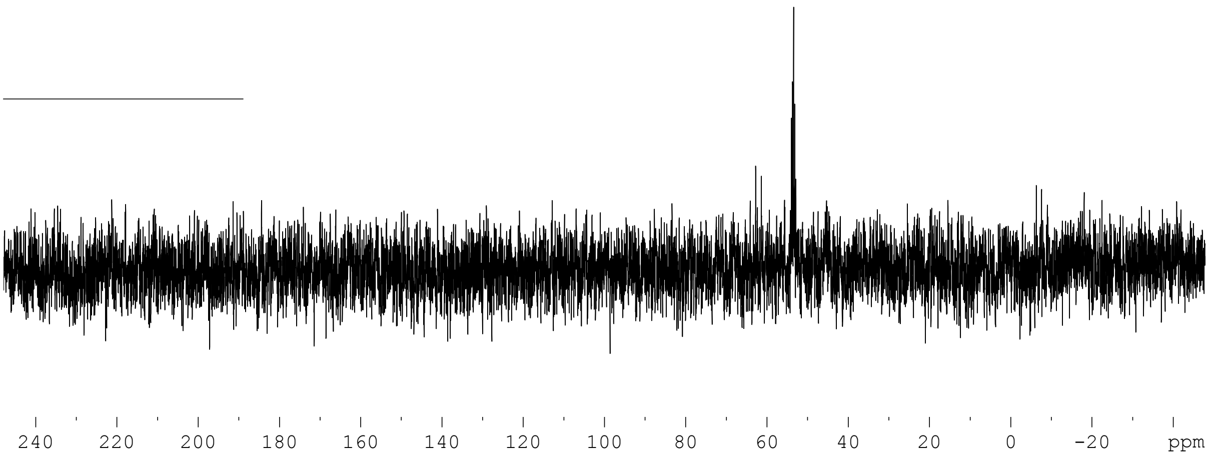

fig. S9c. ^{13}C thermally polarized control NMR spectrum for 1-hexanol showing the CD_2Cl_2 signal at 54 ppm.

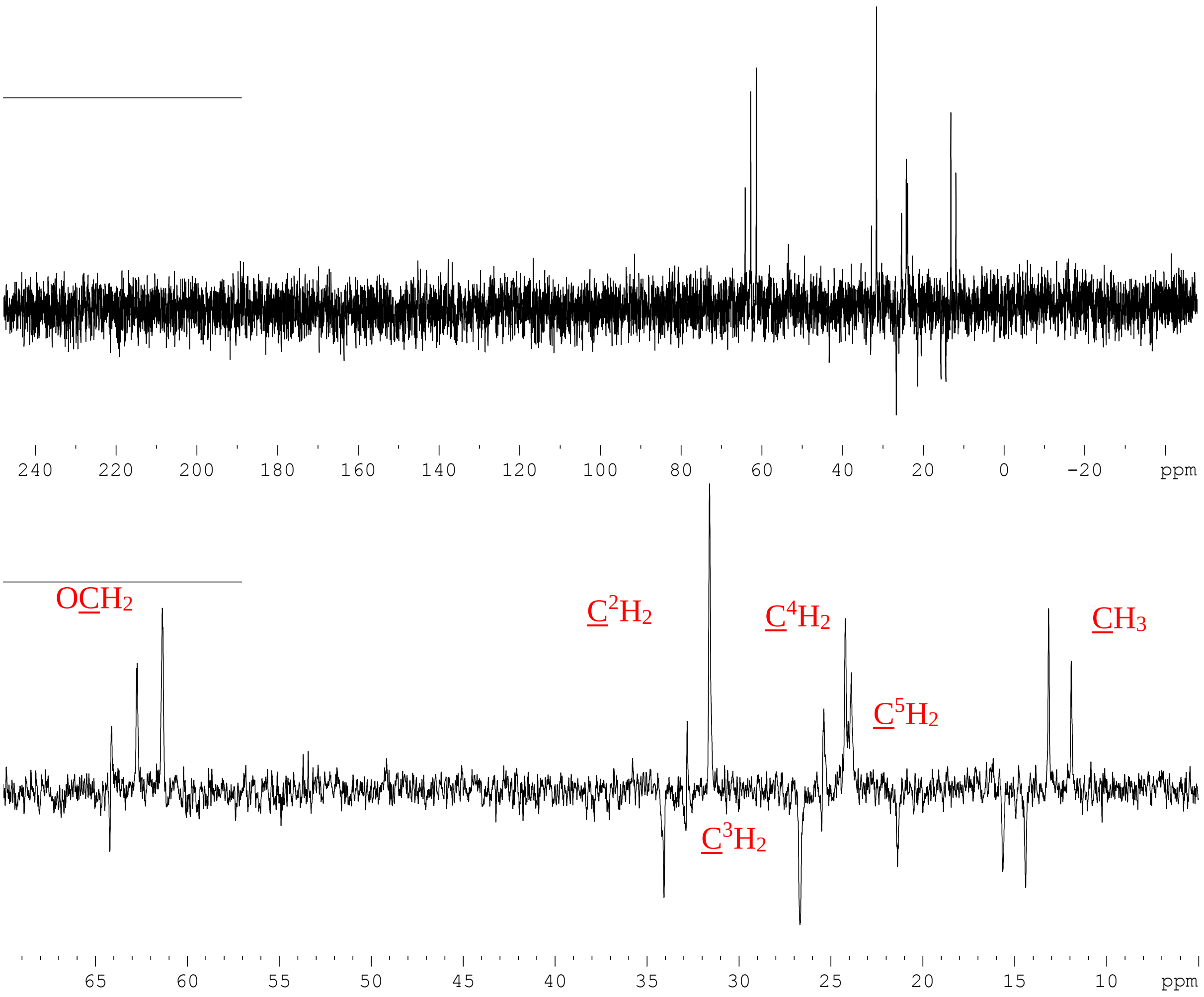

fig. S9d. ^{13}C SABRE-RELAY NMR spectrum for 1-hexanol (top) and expansion (bottom).

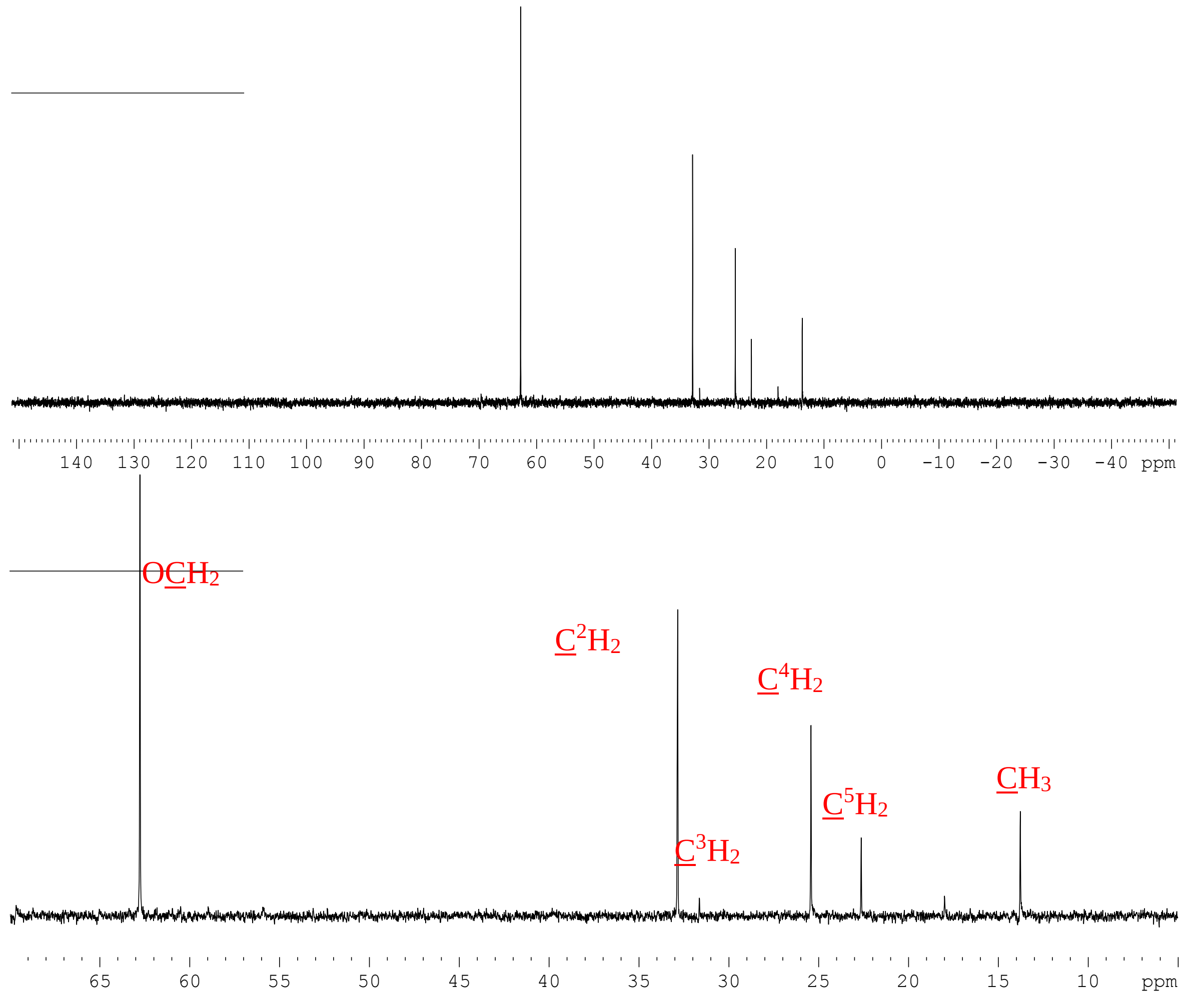

fig. S9e. ^{1}H-^{13}CSABRE-RELAY-refocused INEPT NMR spectrum for 1-hexanol (top) and expansion (bottom). J_{XH} value used was 122.

1-Heptanol

fig. S10. SABRE-RELAY NMR spectra heptanol. (**a** to **e**) reflect a series of single scan NMR spectra: (a) ^{1}H NMR spectrum thermally polarized, (b) ^{1}H SABRE-RELAY NMR spectrum, (c) ^{13}C NMR spectrum thermally polarized , (d) ^{13}C SABRE-RELAY NMR spectrum and (e) ^{1}H-^{13}C refocused INEPT NMR spectrum after SABRE-RELAY for 1-heptanol using conditions: [IrCl(COD)(IMes)] (5 mM), 1-heptanol (1 uL.),CD_2Cl_2 (0.6 mL), NH_3; Substrate Concentration: 11.8 mM. ^{1}H enhancement (fold/per proton): 191 (OH), 253 (-OCH_2-), 107 (-$[CH_2]_5$-), 142 (-CH_3).

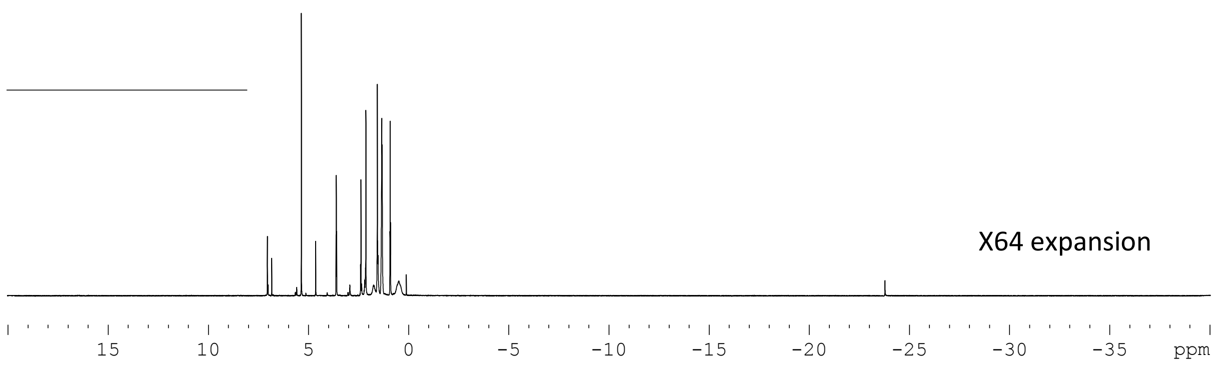

fig. S10a. Thermally polarized ^{1}H NMR reference spectrum for 1-heptanol.

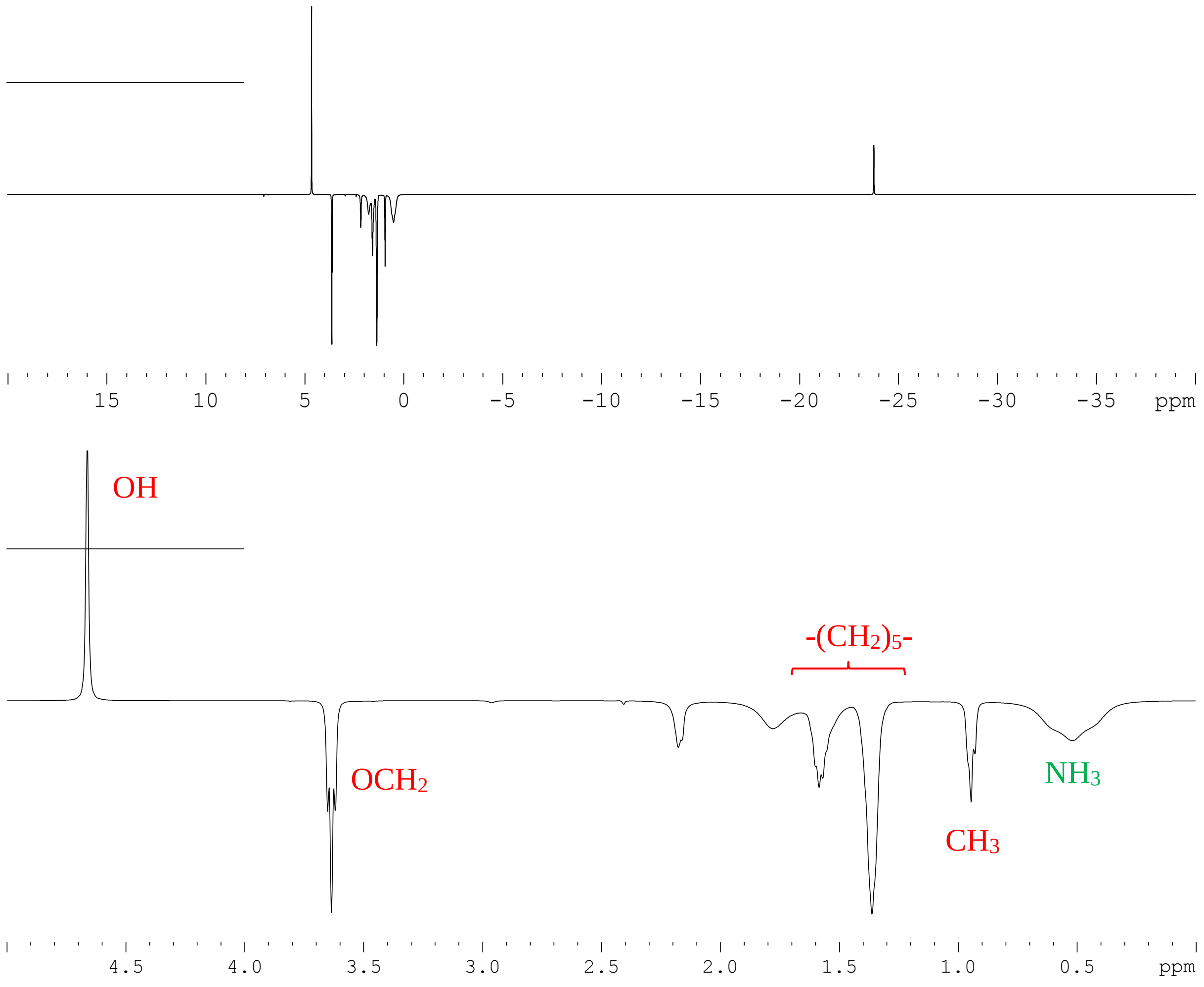

fig. S10b. ^{1}H SABRE-RELAY NMR spectrum for 1-heptanol (top) and expansion (bottom).

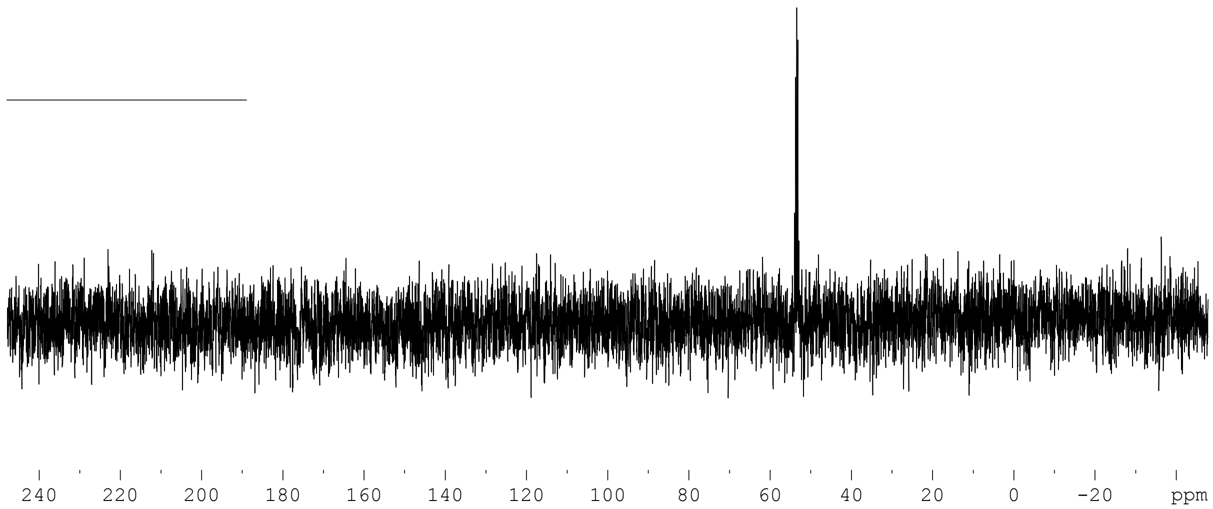

fig. S10c. ^{13}C thermally polarized control NMR spectrum for 1-heptanol showing the CD_2Cl_2 signal at 54 ppm.

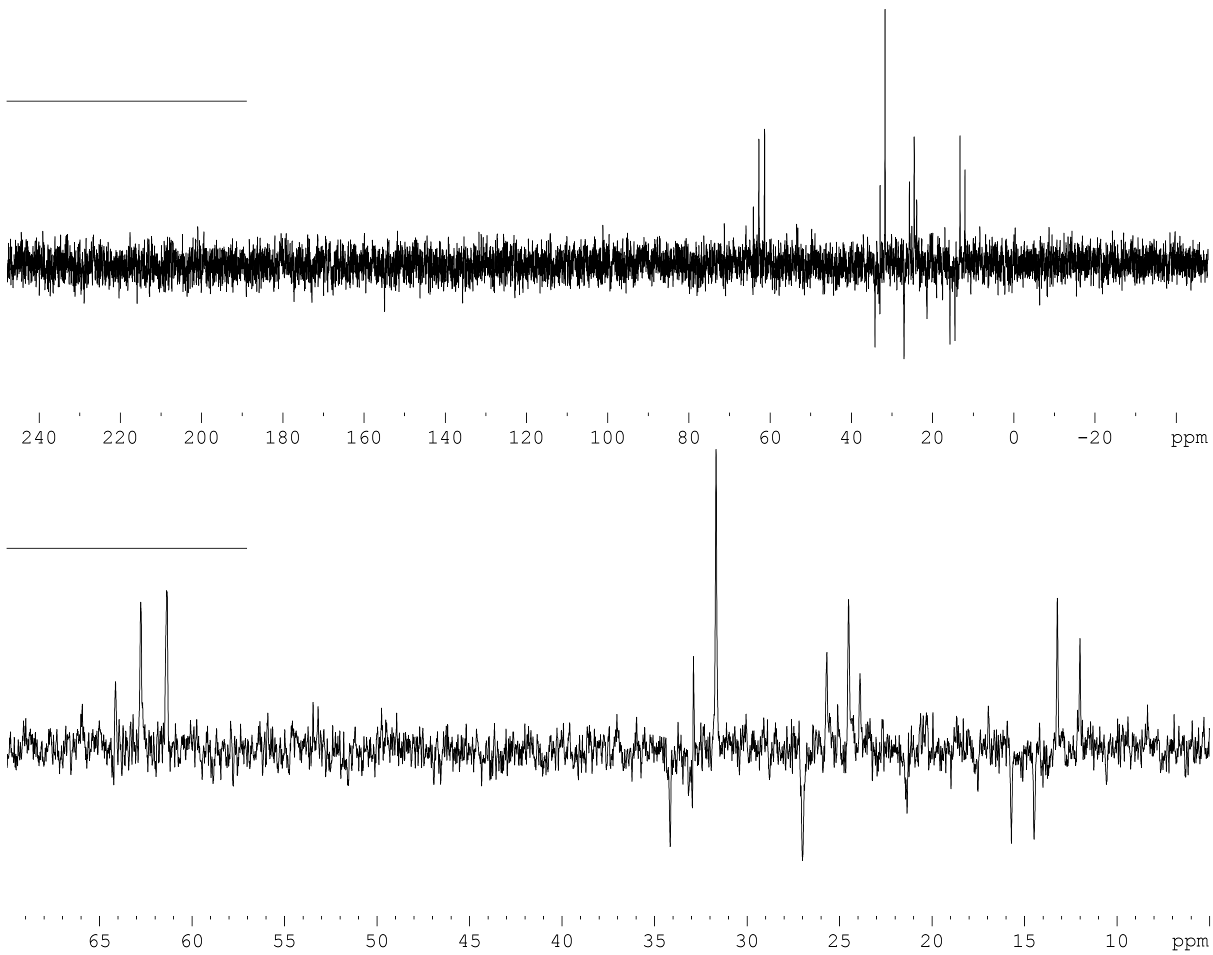

fig. S10d. ^{13}C SABRE-RELAY NMR spectrum for 1-heptanol (top) and expansion (bottom).

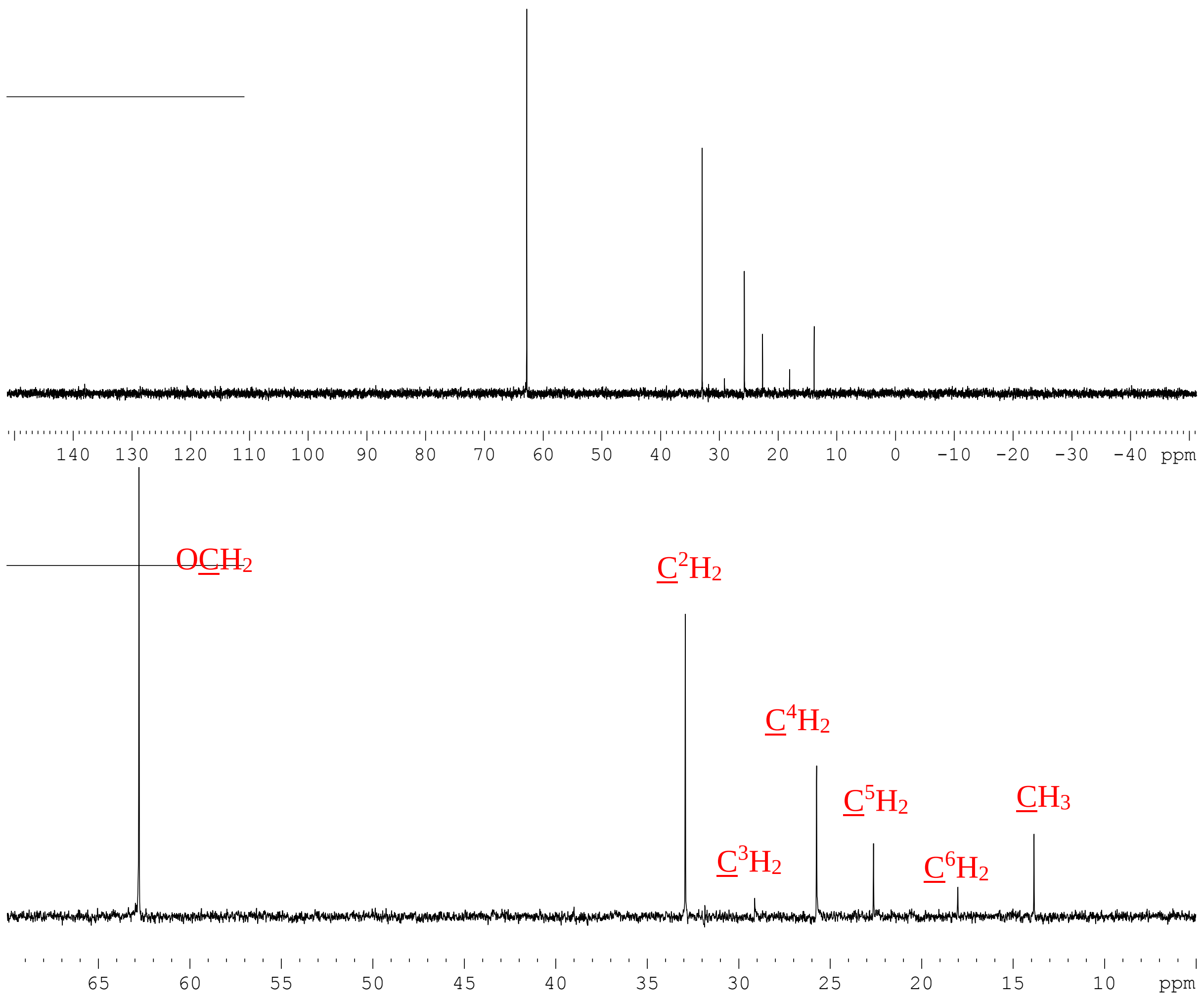

fig. S10e. ^{1}H-^{13}CSABRE-RELAY-refocused INEPT NMR spectrum for 1-heptanol (top) and expansion (bottom). J_{XH} value used was 122.

1-Octanol

fig. S11. SABRE-RELAY NMR spectra octanol. (**a** to **e**) reflect a series of single scan NMR spectra: (a) ^{1}H NMR spectrum thermally polarized, (b) ^{1}H SABRE-RELAY NMR spectrum, (c) ^{13}C NMR spectrum thermally polarized , (d) ^{13}C SABRE-RELAY NMR spectrum and (e) ^{1}H-^{13}C refocused INEPT NMR spectrum after SABRE-RELAY for 1-octanol using conditions: [IrCl(COD)(IMes)] (5 mM), 1-octanol (1 uL.),CD_2Cl_2 (0.6 mL), NH_3; Substrate Concentration: 10.6 mM. ^{1}H enhancement (fold/per proton): 125 (OH), 274 (-OCH_2-), 98 (-$[CH_2]_6$-), 171 (-CH_3).

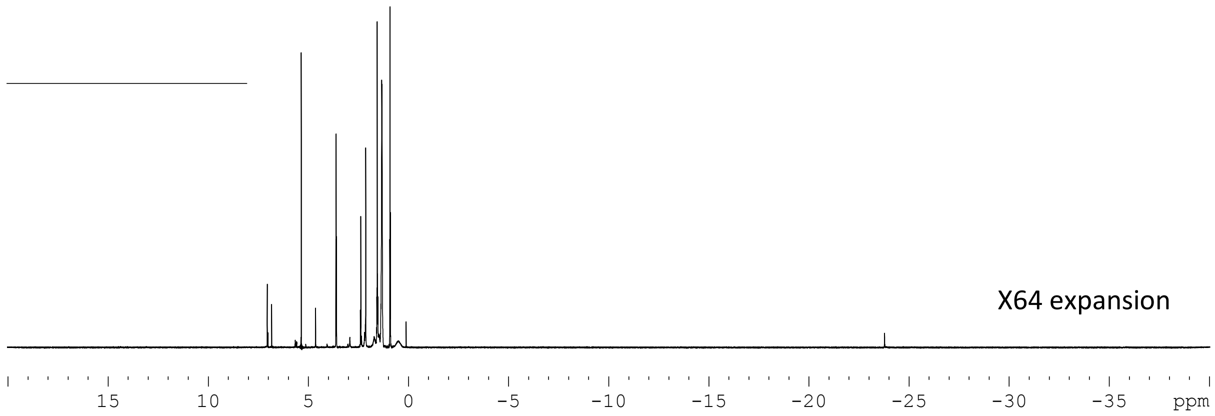

fig. S11a. Thermally polarized ^{1}H NMR reference spectrum for the 1-octanol doped sample.

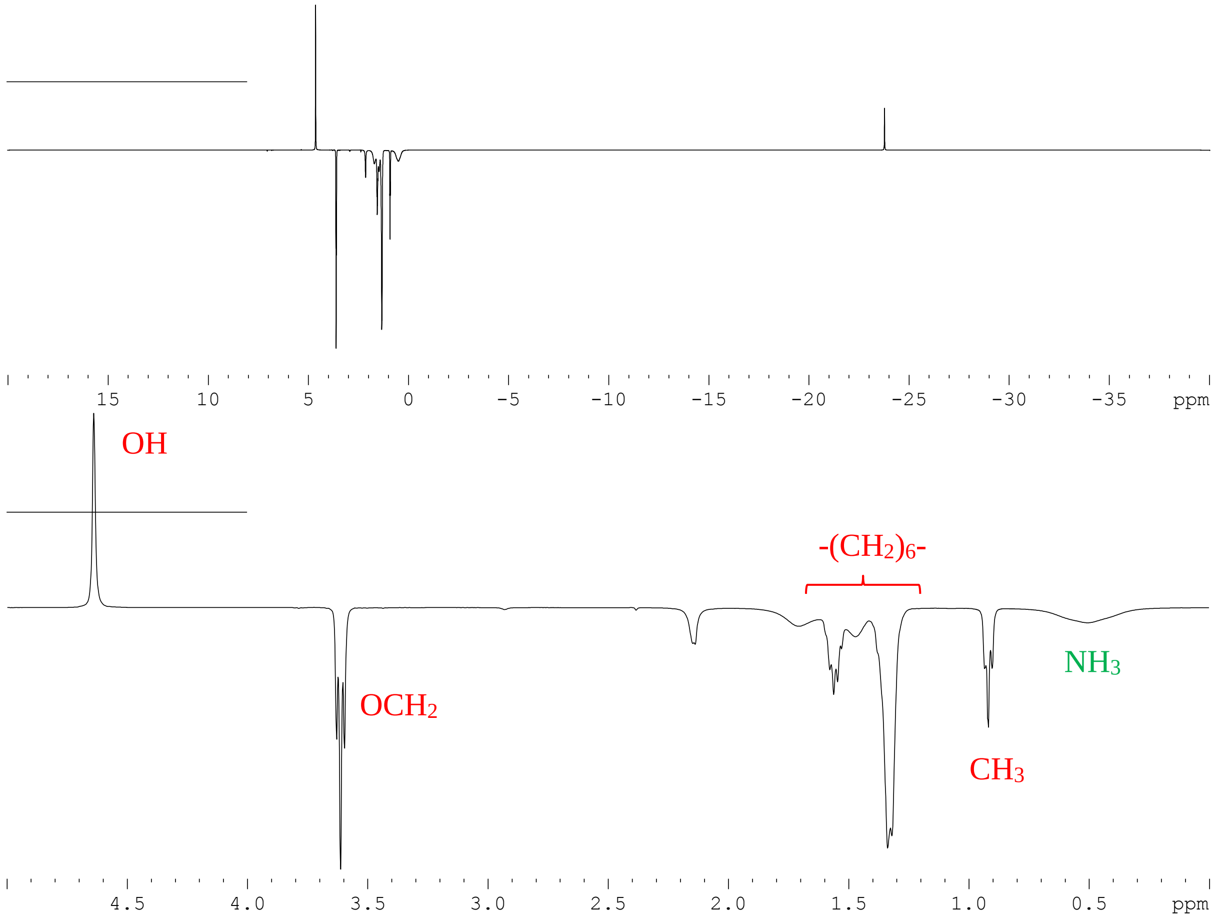

fig. S11b. ^{1}H SABRE-RELAY NMR spectrum for 1-octanol (top) and expansion (bottom).

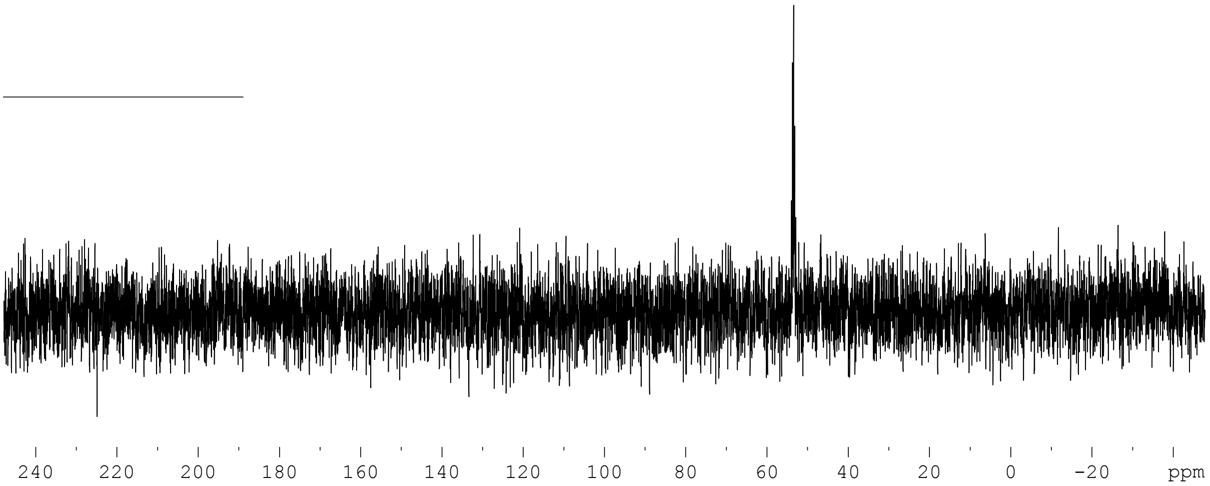

fig. S11c. ^{13}C thermally polarized background NMR spectrum for the 1-octanol sample showing the CD_2Cl_2 signal at 54 ppm.

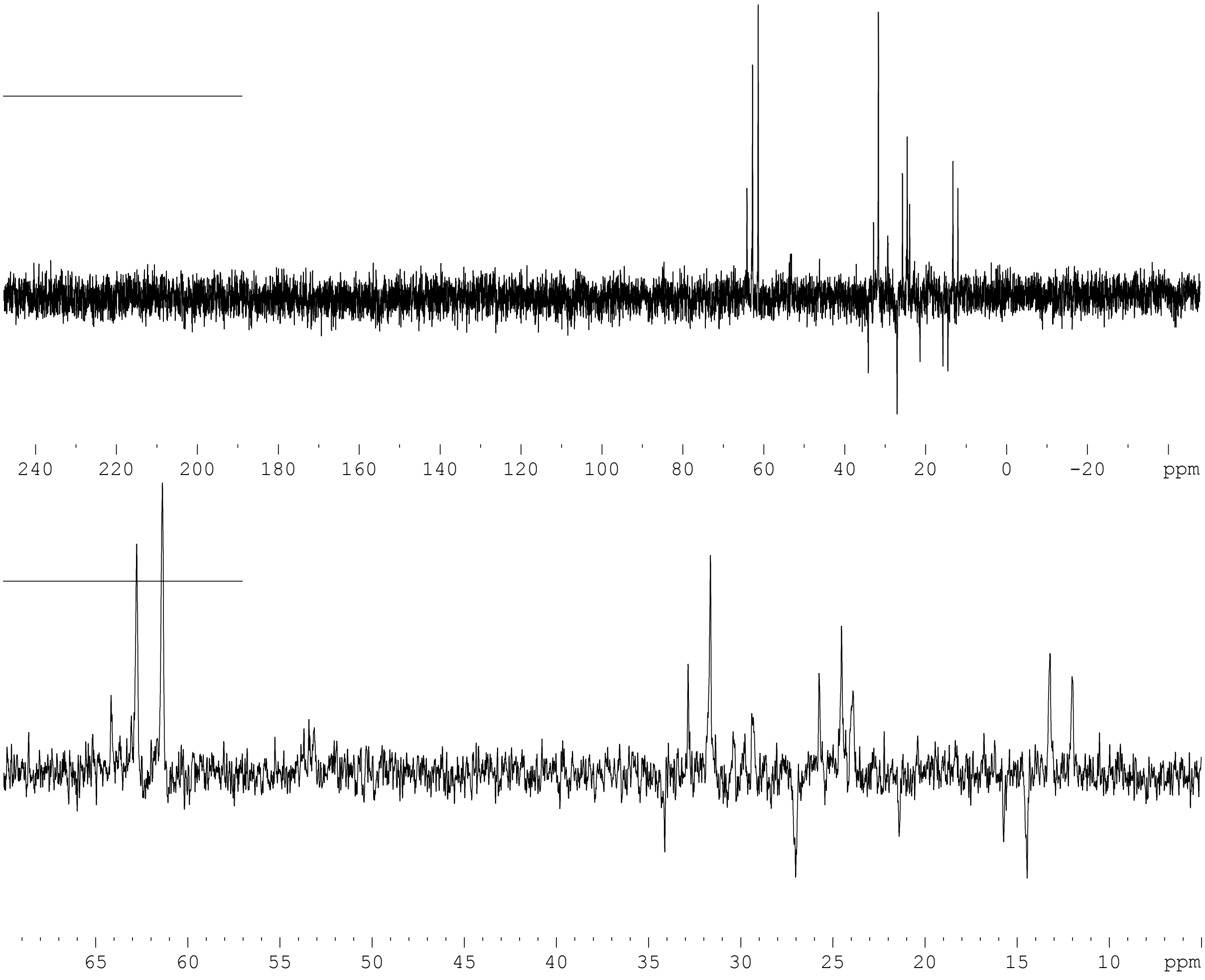

fig. S11d. ^{13}C SABRE-RELAY NMR spectrum for 1-octanol.

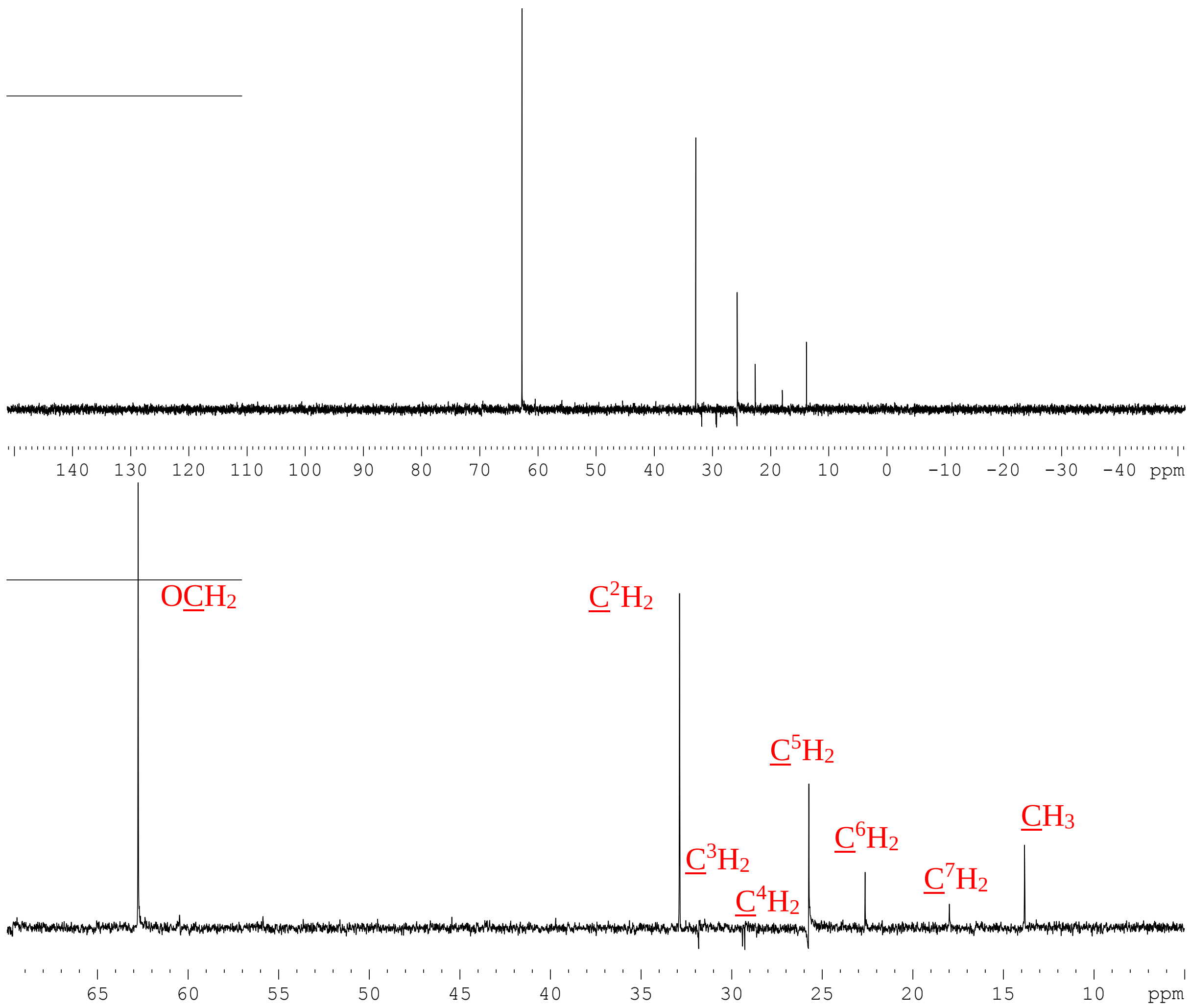

fig. S11e. ^{1}H-^{13}CSABRE-RELAY-refocused INEPT NMR spectrum for 1-octanol. J_{XH} value used was 122.

Isopropanol

fig. S12. SABRE-RELAY NMR spectra isopropanol. (**a** to **f**) reflect a series of NMR spectra: (a) ^{1}H NMR spectrum thermally polarized, (b) ^{1}H SABRE-RELAY NMR spectrum, (c) ^{13}C NMR spectrum thermally polarized , (d) ^{13}C SABRE-RELAY NMR spectrum, (e) ^{1}H-^{13}C refocused INEPT NMR spectrum after SABRE-RELAY and (f) ^{13}C SABRE-RELAY-DEPT NMR spectrum for isopropanol using conditions: [IrCl(COD)(IMes)] (5 mM), isopropanol (1 uL.),CD_2Cl_2 (0.6 mL), NH_3; Substrate Concentration: 22 mM. ^{1}H enhancement (fold/per proton): 69 (OH), 63 (-OCH-), 42 (-[CH_3]$_3$).

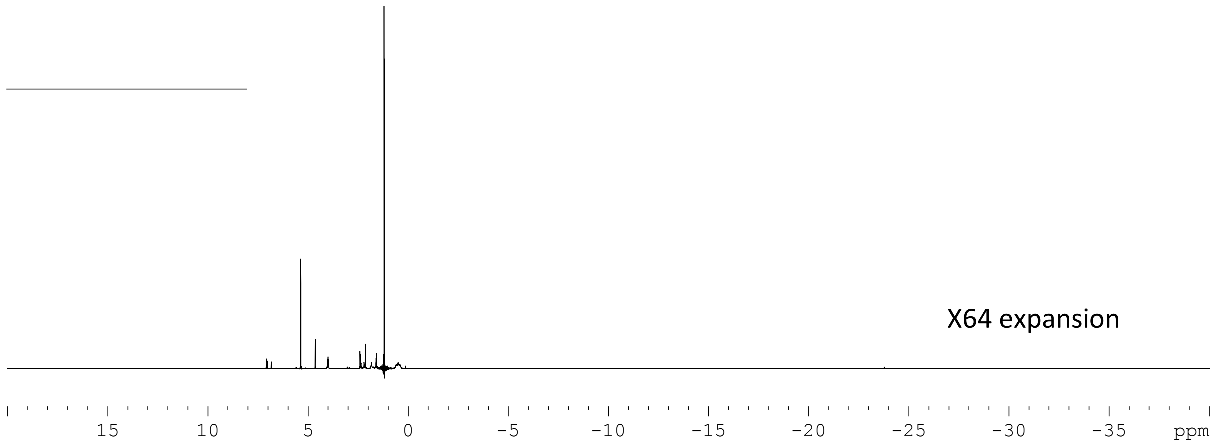

fig. S12a. Thermally polarized ^{1}H NMR reference spectrum for isopropanol.

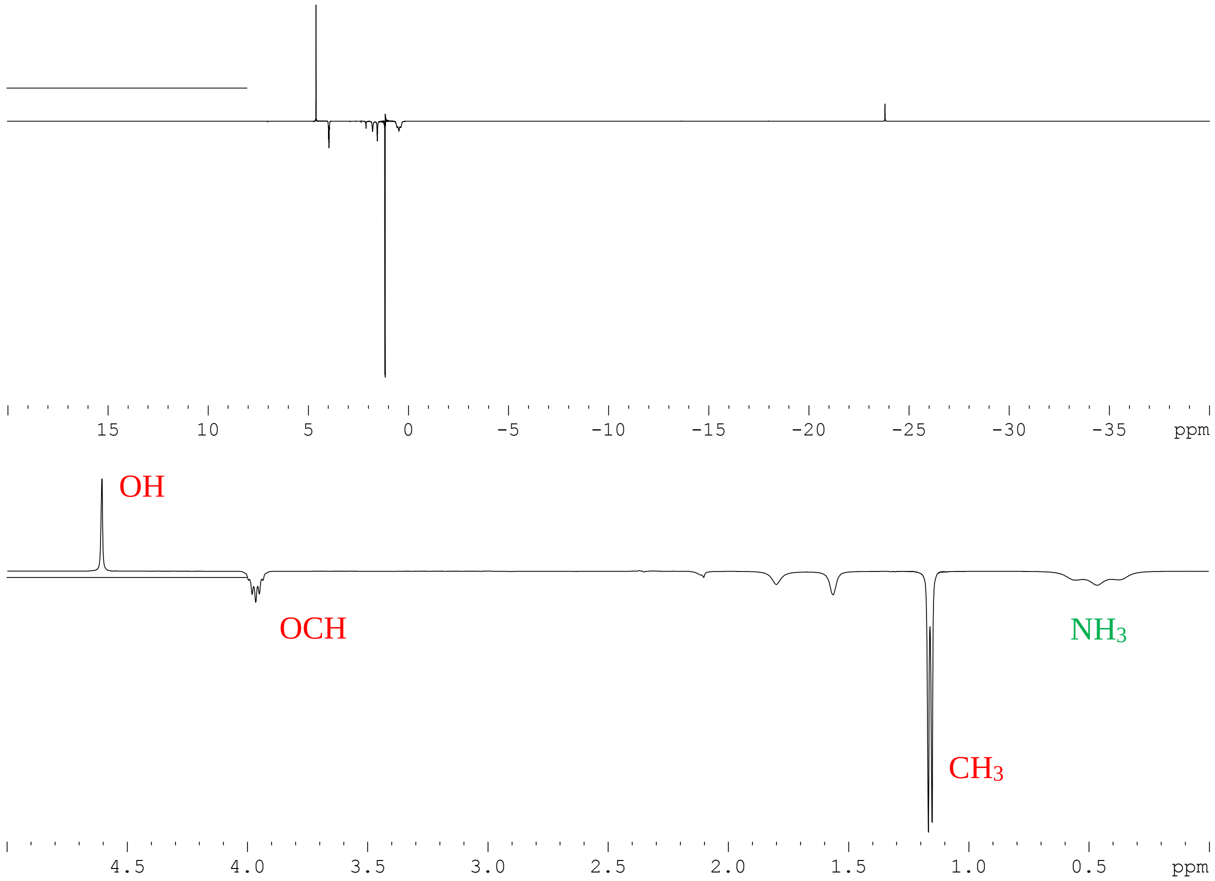

fig. S12b. ^{1}H SABRE-RELAY NMR spectrum for isopropanol (top) and expansion (bottom).

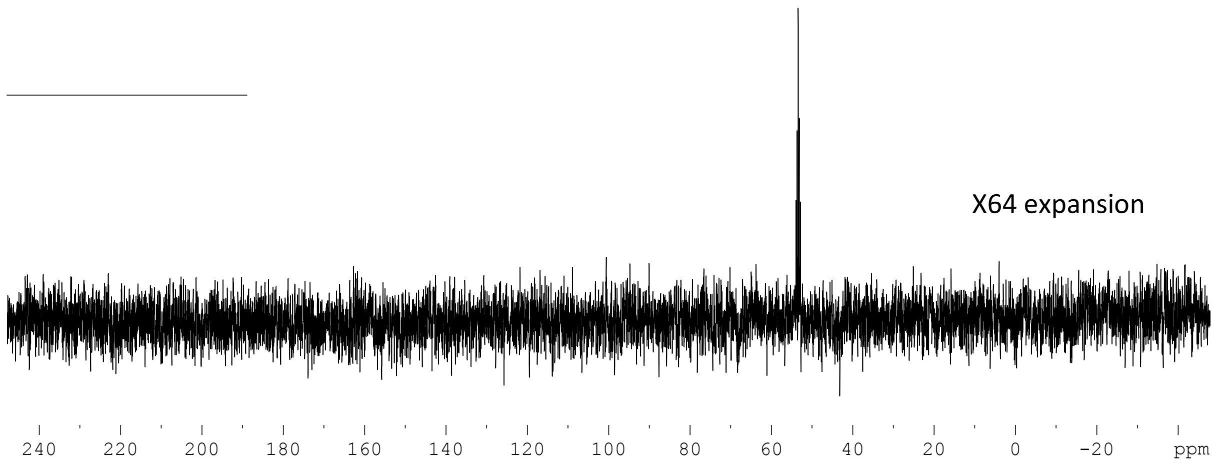

fig. S12c. Thermally polarized ^{13}C NMR reference spectrum for isopropanol.

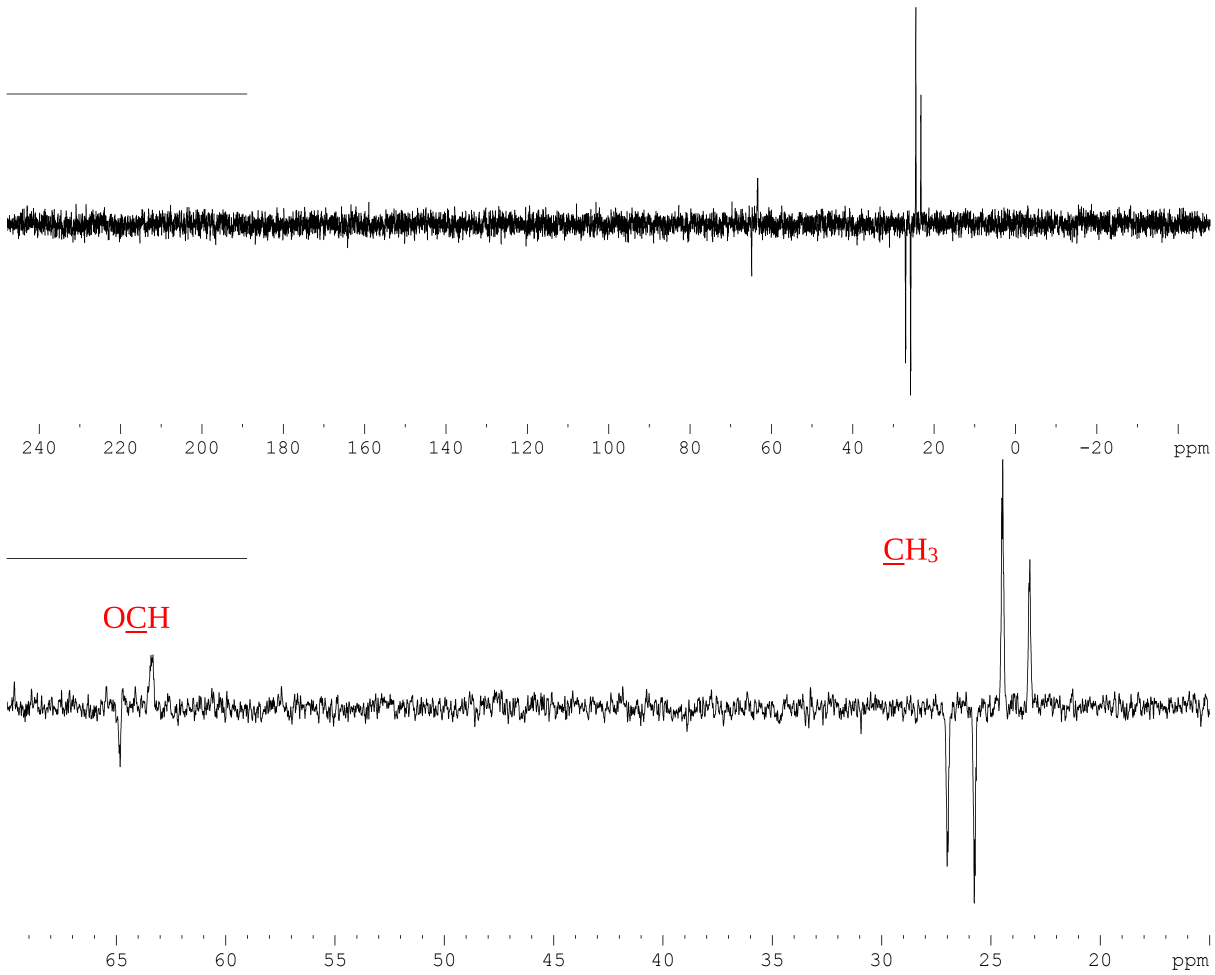

fig. S12d. ^{13}C SABRE-RELAY NMR spectrum for isopropanol (top) and expansion (bottom).

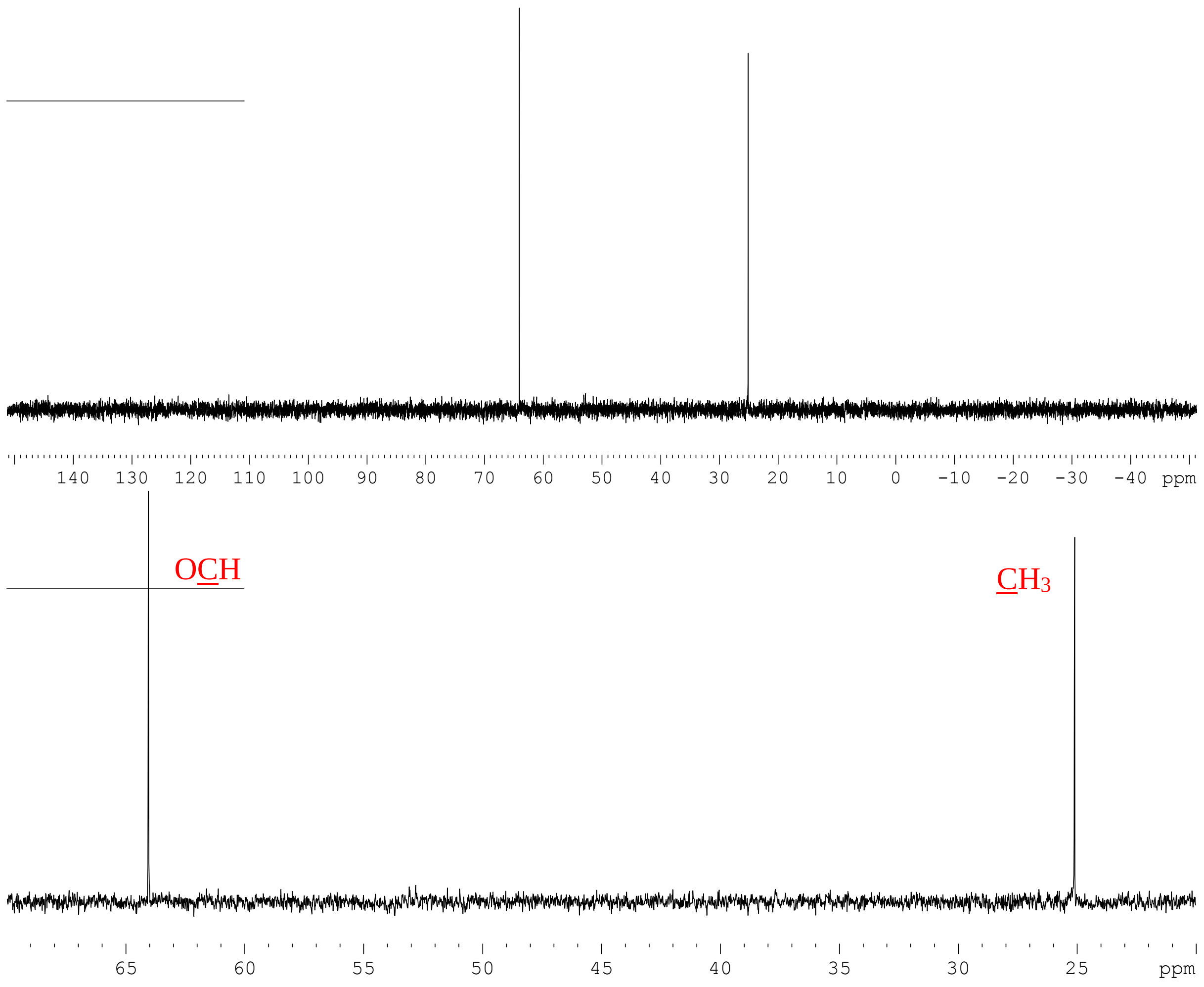

fig. S12e. ^{1}H-^{13}CSABRE-RELAY-refocused INEPT NMR spectrum for isopropanol (top) and expansion (bottom). J_{XH} value used was 122.

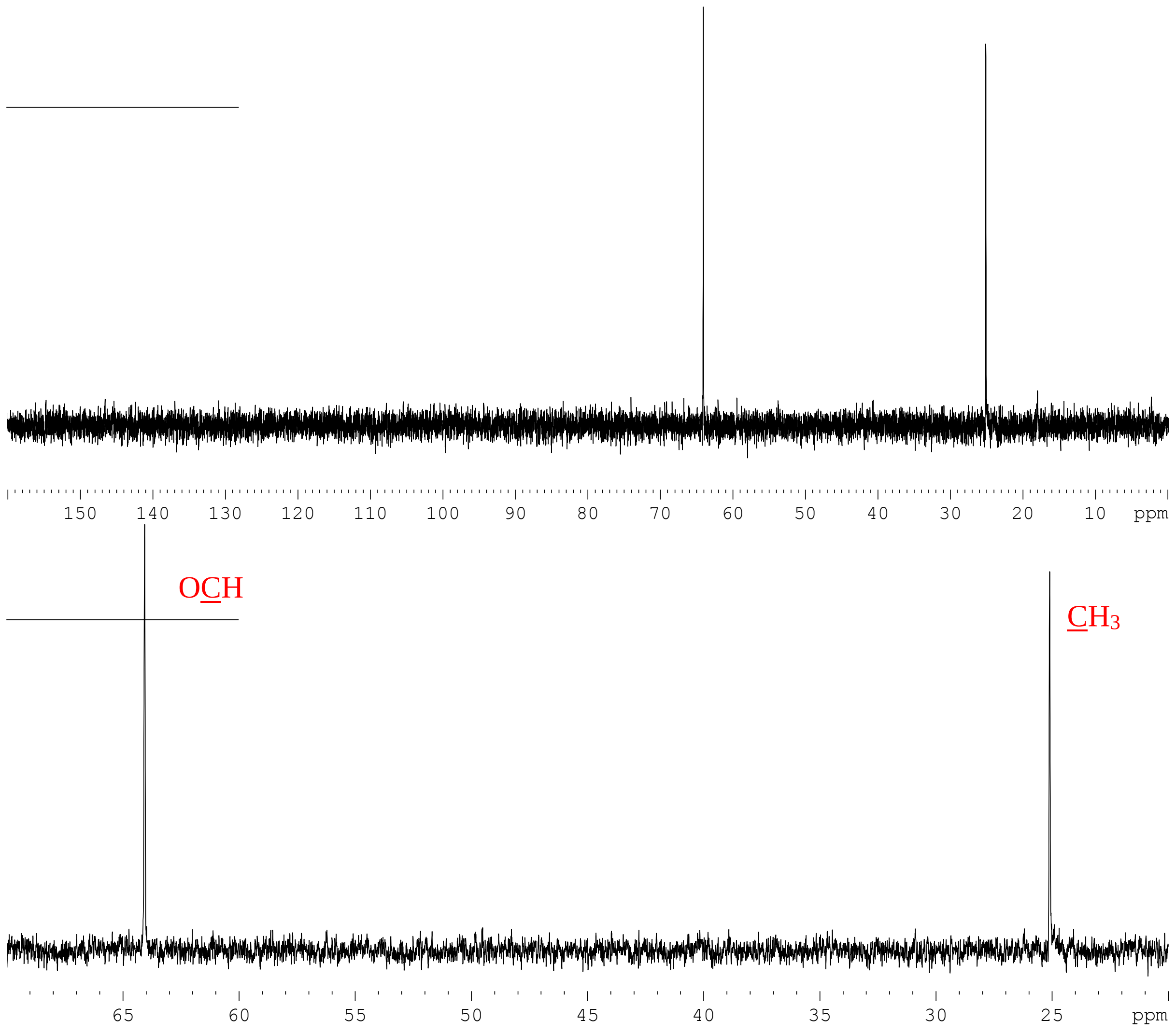

fig. S12f. ^{1}H-^{13}CSABRE-RELAY-DEPT NMR spectrum for isopropanol (top) and expansion (bottom). J_{XH} value used was 122.

***Tert*-butanol**

fig. S13. SABRE-RELAY NMR spectra *tert*-butanol. (**a** to **b**) reflect a series of NMR spectra: (a) ^{1}H NMR spectrum, thermally polarized, (b) ^{1}H SABRE-RELAY NMR spectrum for t-butanol using conditions: [IrCl(COD)(IMes)] (5 mM), tert-butanol (1 uL.),CD_2Cl_2 (0.6 mL), NH_3; Substrate Concentration: 18 mM. ^{1}H enhancement (fold/per proton): 480 (OH), 154 (-$[CH_3]_3$).

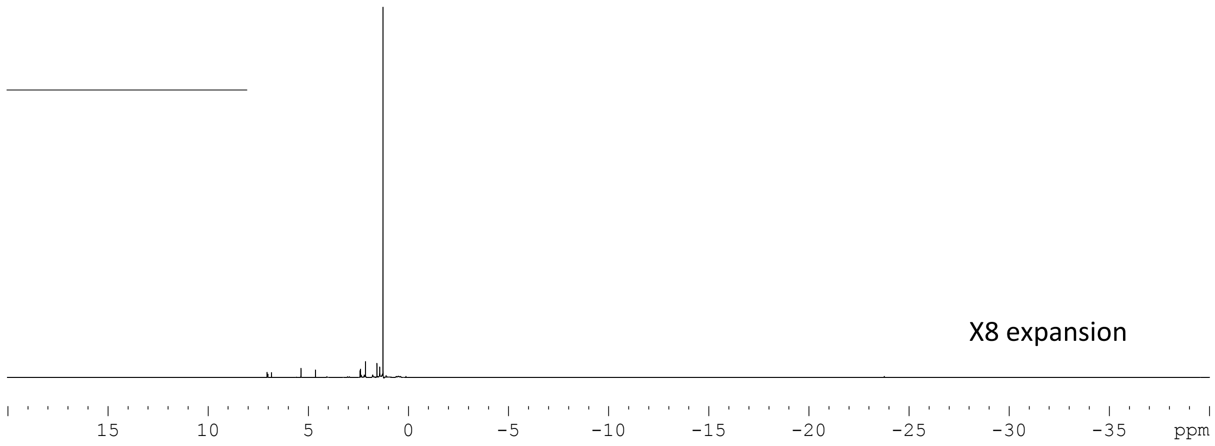

fig. S13a. Thermally polarized ^{1}H NMR reference spectrum for t-butanol.

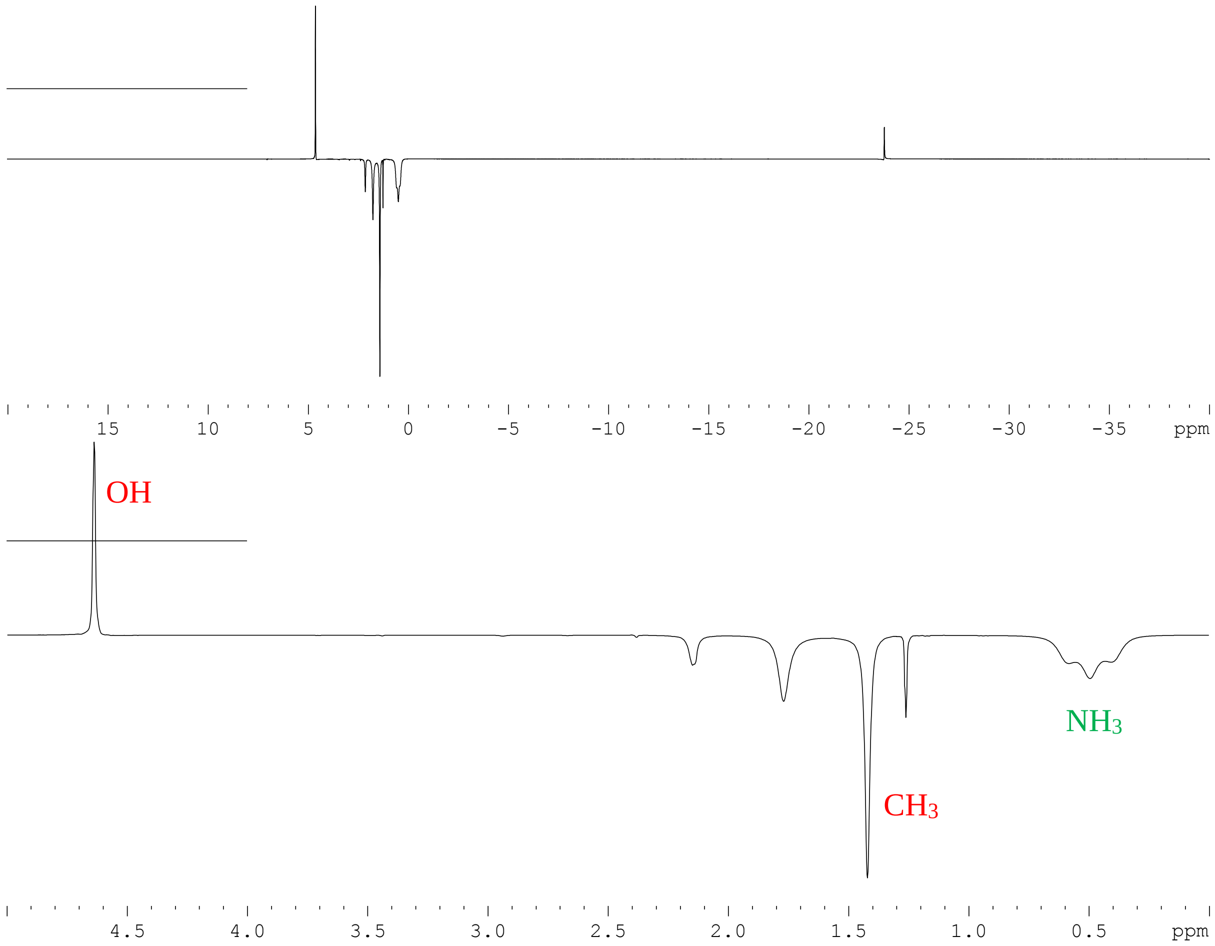

fig. S13b. ^{1}H SABRE-RELAY NMR spectrum for t-butanol (top) and expansion (bottom).

D-glucose

fig. S14. SABRE-RELAY NMR spectra D-glucose. (**a** to **b**) reflect a series of NMR spectra for a sample containing a 10 fold excess of glucose: (a) ^{1}H NMR spectrum thermally polarized x4, (b) ^{1}H SABRE-RELAY NMR spectrum for D-glucose-$^{13}C_6$; Substrate Concentration: 50 mM. Acetone was employed as the solvent with 50 μL of DMSO due to low solubility. ^{1}H enhancement (fold/per proton): 40

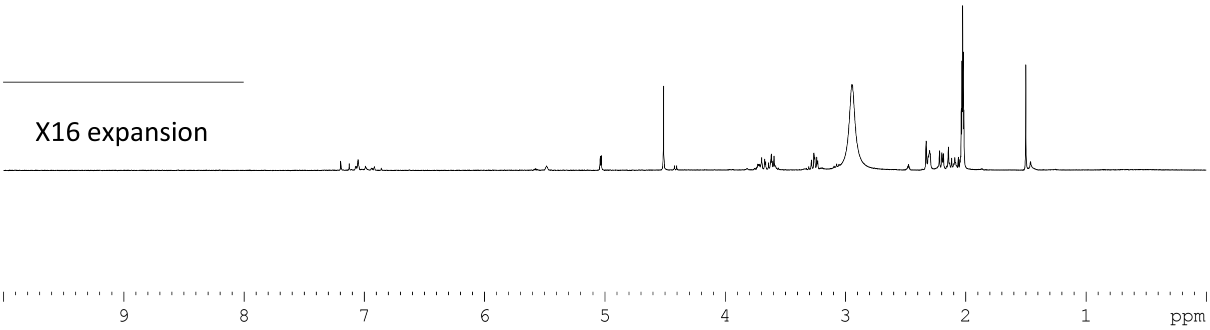

fig. S14a. ^{1}H thermally polarized NMR reference spectrum for D-glucose x16.

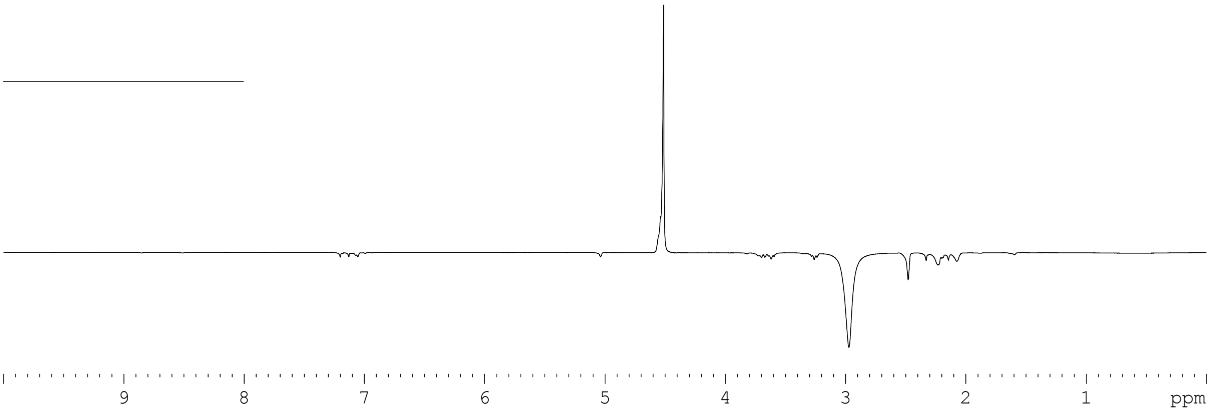

fig. S14b. ^{1}H SABRE-RELAY NMR spectrum for D-glucose.

D-glucose-$^{13}C_6$

fig. S15. SABRE-RELAY NMR spectra D-glucose-^{13}C. (**a** to **e**) reflect a series of NMR spectra: (a) ^{1}H NMR spectrum thermally polarized, (b) ^{1}H SABRE-RELAY NMR spectrum, (c) $^{13}C\{^1H\}$ NMR spectrum thermally polarized, (d) $^{13}C\{^1H\}$ NMR spectrum SABRE-RELAY and (e) ^{1}H-^{13}C refocused INEPT NMR spectrum after SABRE-RELAY for D-glucose-$^{13}C_6$ using conditions: [IrCl(COD)(1,3-bis(2,4,6-trimethylphenyl)-4,5-

dimethylimidazole)] (5 mM), glucose-$^{13}C_6$ (10 eq.),CD_2Cl_2 (0.6 mL), PEA (1 µL), H_2O (5 µL); Substrate Concentration: 50 mM.

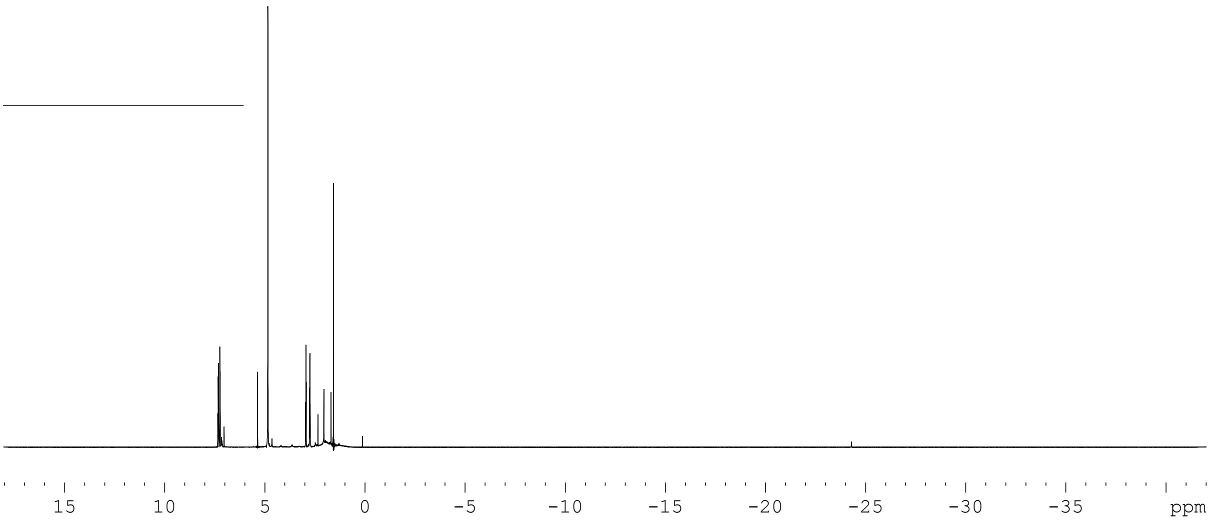

fig. S15a. ^{1}H thermally polarized polarized background NMR spectrum for D-glucose-$^{13}C_6$.

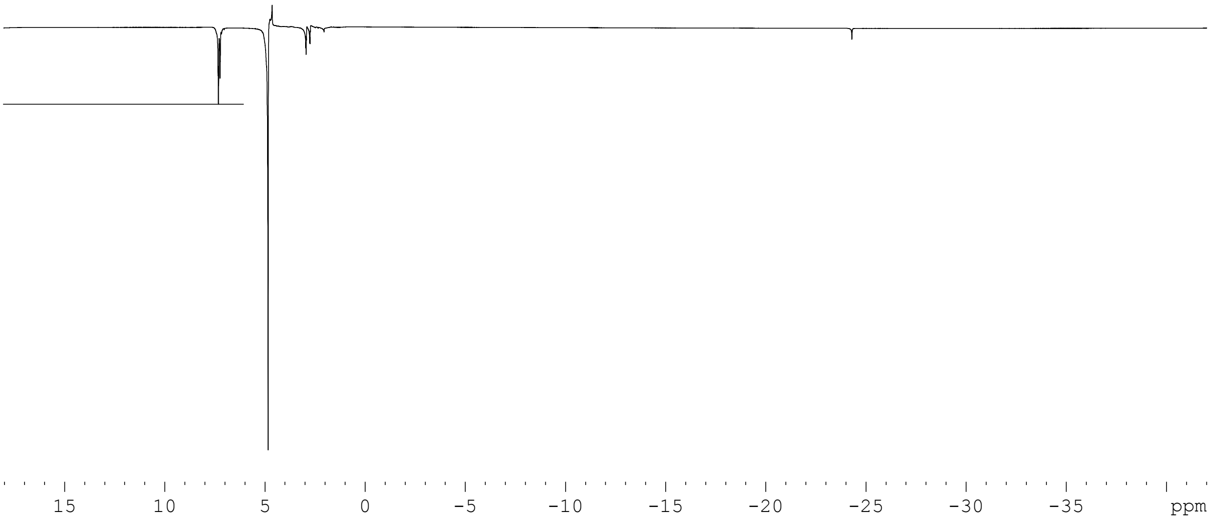

fig. S15b. ^{1}H SABRE-RELAY NMR spectrum for D-glucose-$^{13}C_6$.

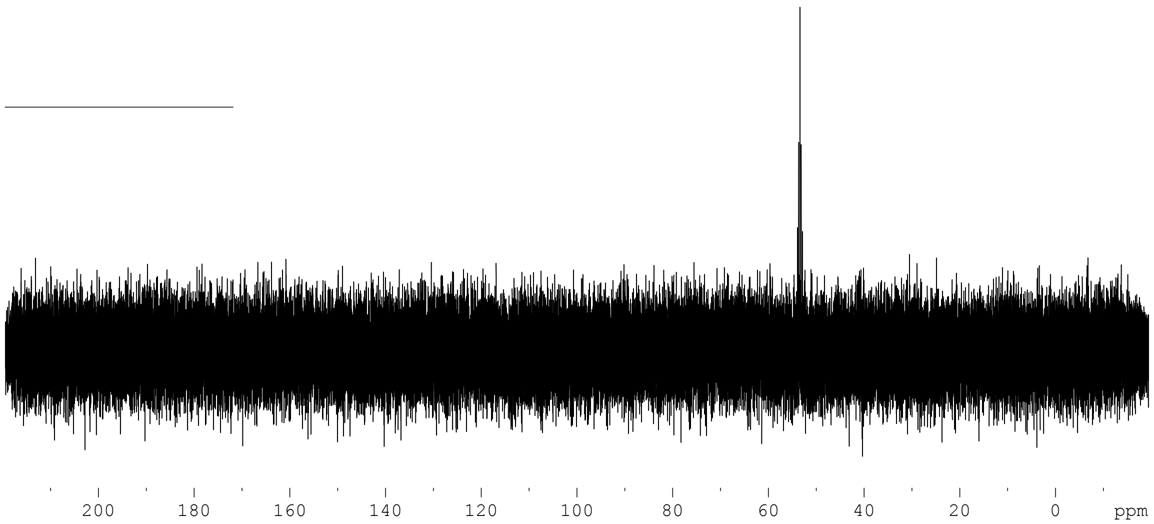

fig. S15c. Thermally polarized $^{13}C\{^1H\}$NMR reference spectrum for D-glucose-$^{13}C_6$.

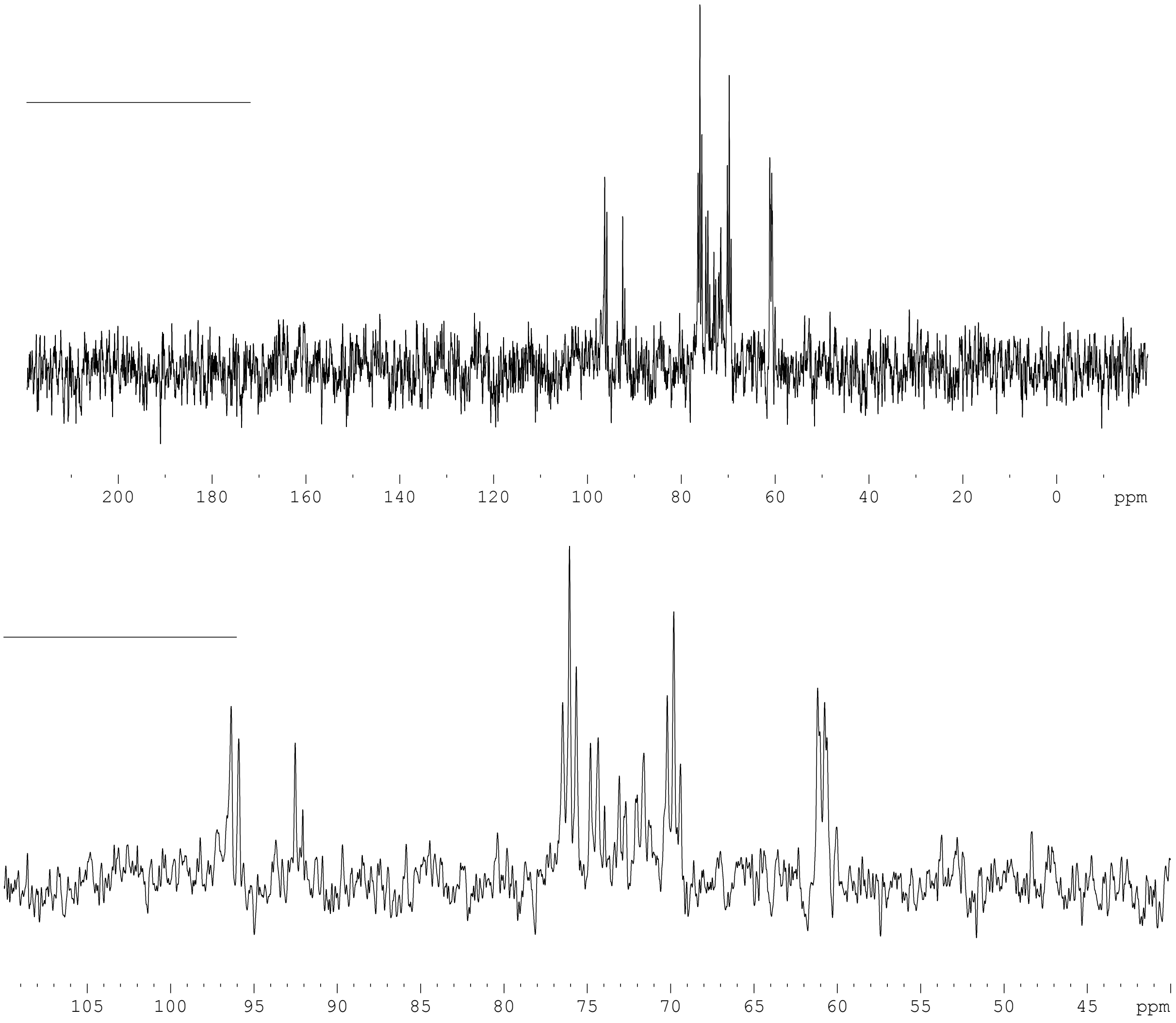

fig. S15d. $^{13}C\{^1H\}$ SABRE-RELAY NMR spectrum for D-glucose-$^{13}C_6$ (top) and expansion (bottom).

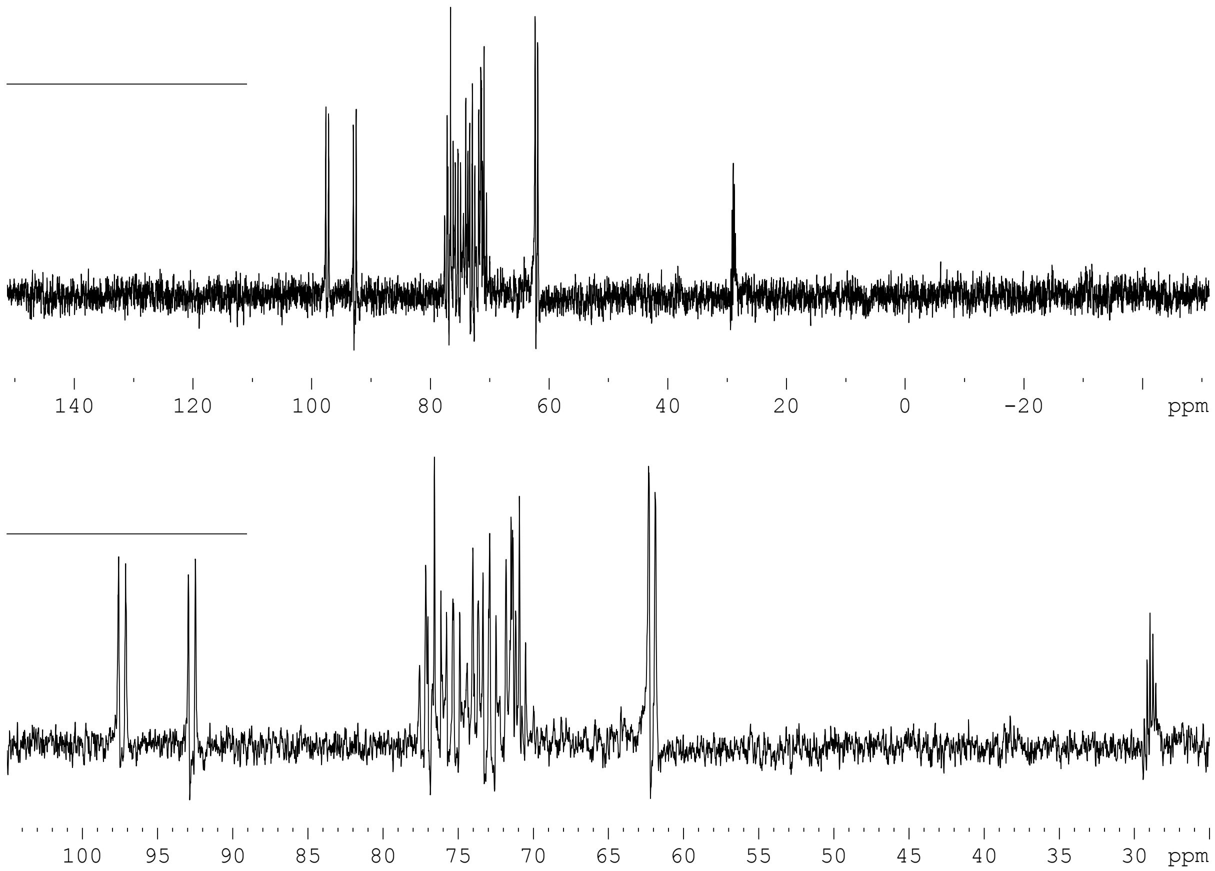

fig. S15e. ^{1}H-^{13}CSABRE-RELAY refocused INEPT spectrum for D-glucose-$^{13}C_6$ (top) and expansion (bottom). J_{XH} value used was 122.

Glycerol

fig. S16. SABRE-RELAY NMR spectra glycerol. (**a** to **b**) reflect a series of NMR spectra: (a) ^{1}H NMR spectrum thermally polarized and (b) ^{1}H SABRE-RELAY NMR spectrum for glycerol using conditions: [IrCl(COD)(IMes)] (5 mM), glycerol (20 eq.), CD_2Cl_2 (0.6 mL), NH_3; Substrate Concentration: 100 mM. ^{1}H enhancement (fold/per proton): 15 (OH), 7.5 (OCH).

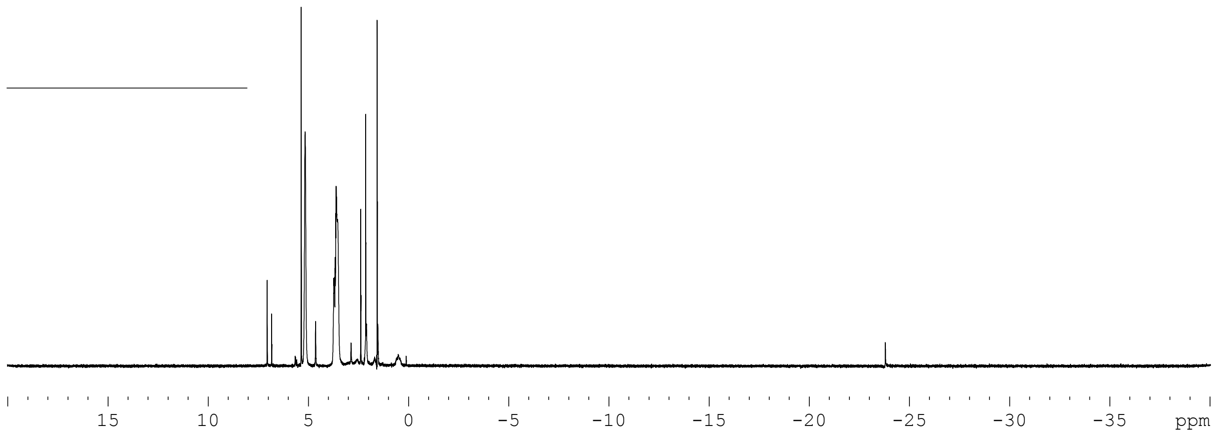

fig. S16a. Thermally polarized ^{1}H NMR reference spectrum for glycerol.

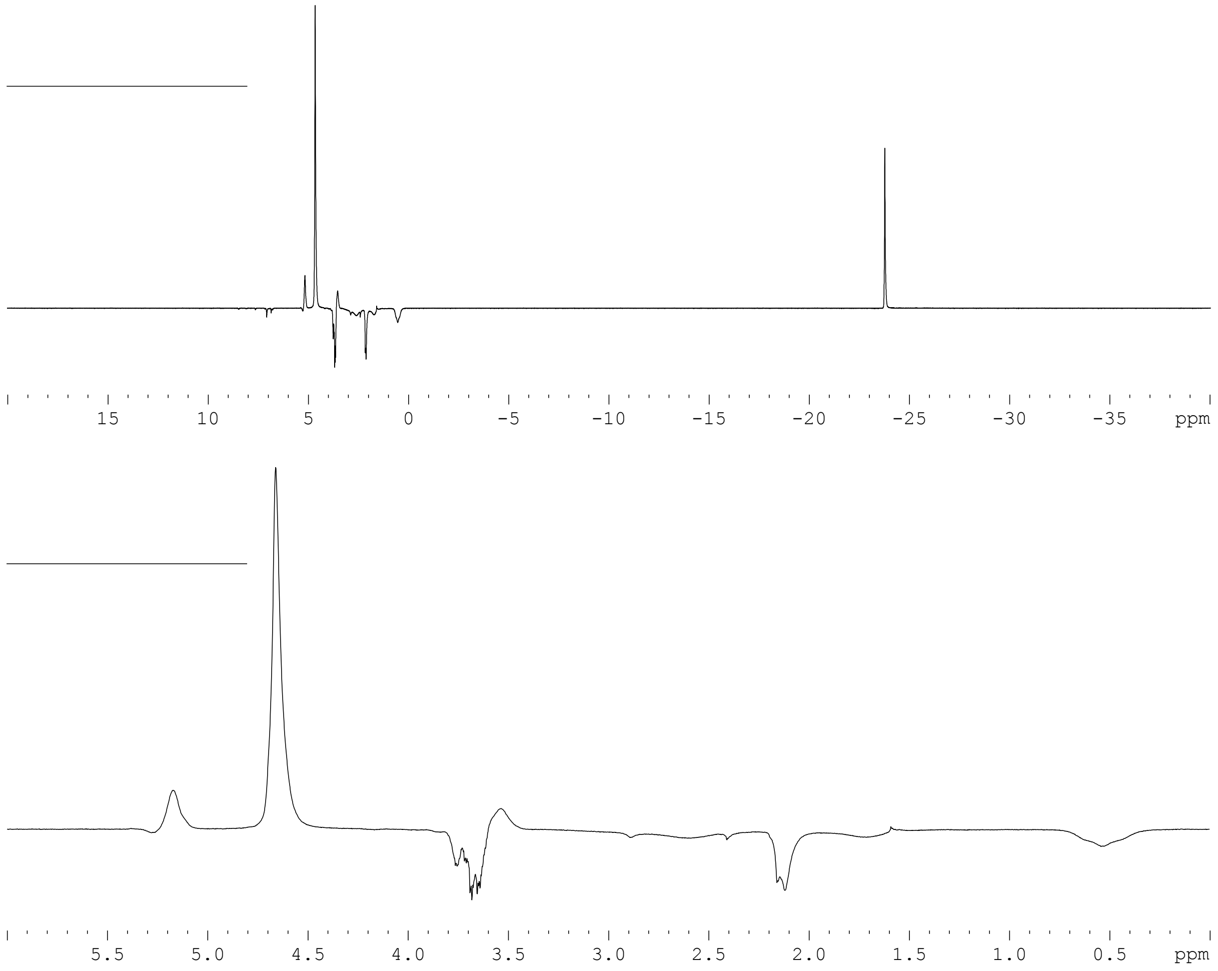

fig. S16b. ^{1}H SABRE-RELAY NMR spectrum for glycerol (top) and expansion (bottom).

Carboxylic Acids and Carboxylates

Sodium Acetate-1-^{13}C

fig. S17. SABRE-RELAY NMR spectra sodium acetate-^{13}C. (**a** to **d**) reflect a series of NMR spectra: (a) ^{1}H NMR spectrum thermally polarized, (b) ^{1}H SABRE-RELAY NMR spectrum, (c) ^{13}C NMR spectrum thermally polarized and (d) ^{13}C SABRE-RELAYNMR spectrum for sodium acetate-1-^{13}C using conditions: [IrCl(COD)(IMes)] (5 mM), sodium acetate-1-^{13}C (10 eq.),CD_2Cl_2 (0.6 mL), NH_3, H_2O (5 µL); Substrate Concentration: 50 mM. ^{13}C enhancement (fold/per ^{13}C): 48.

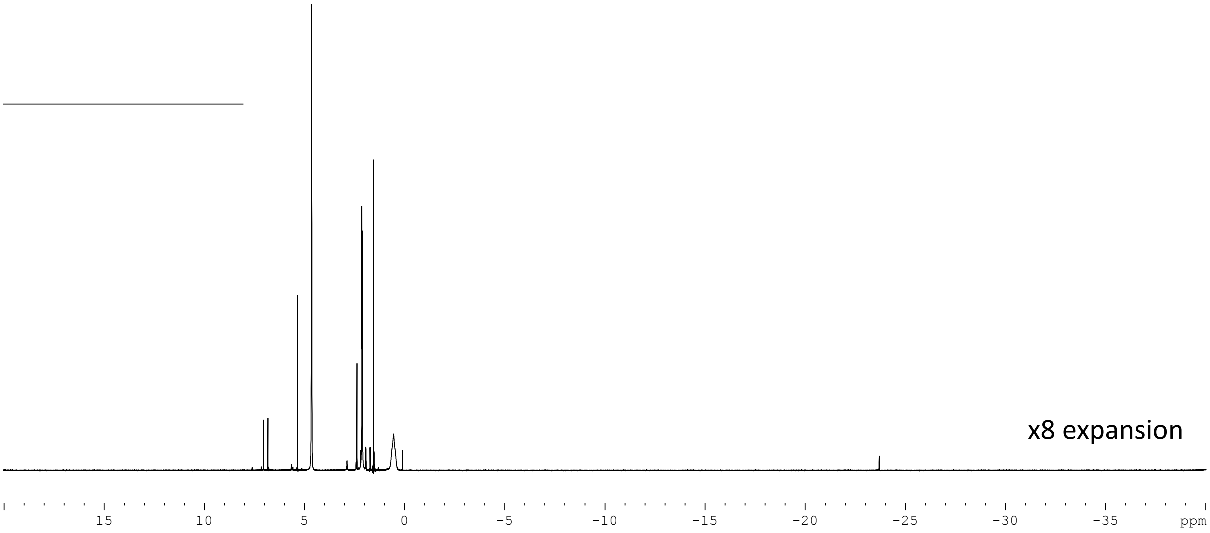

fig. S17a. Thermally polarized ^{1}H NMR reference spectrum for sodium acetate-1-^{13}C.

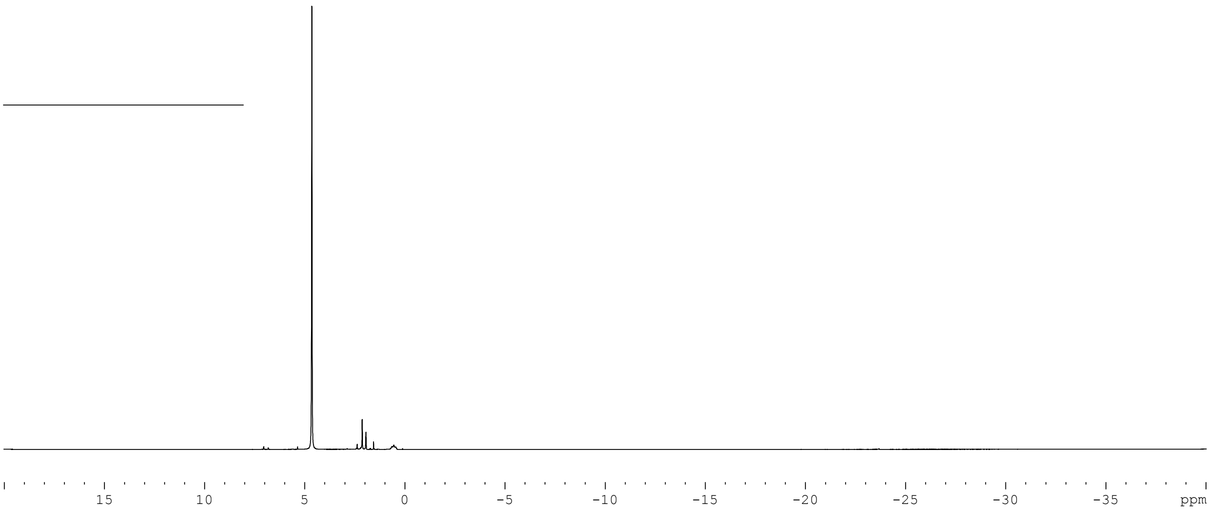

fig. S17b. ^{1}H SABRE-RELAY NMR spectrum for sodium acetate-1-^{13}C.

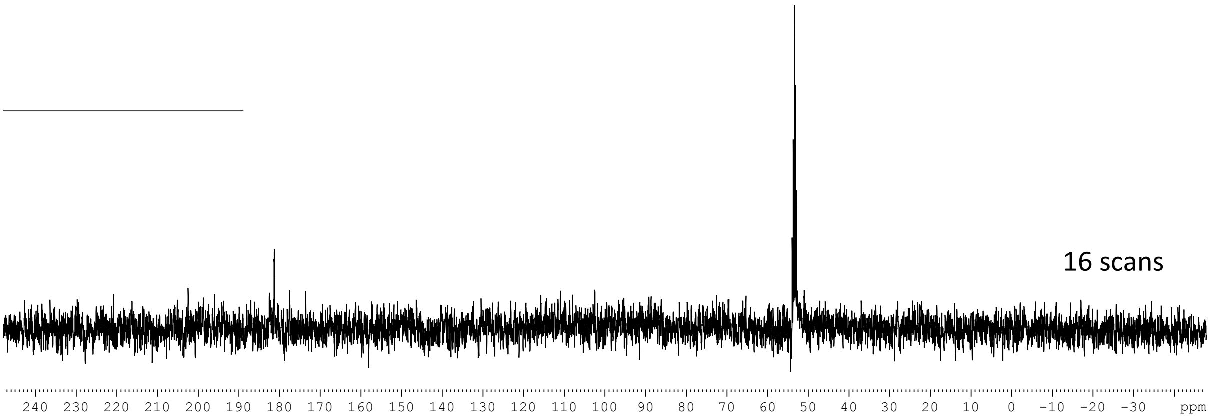

fig. S17c. Thermally polarized ^{13}C NMR reference spectrum for sodium acetate-1-^{13}C.

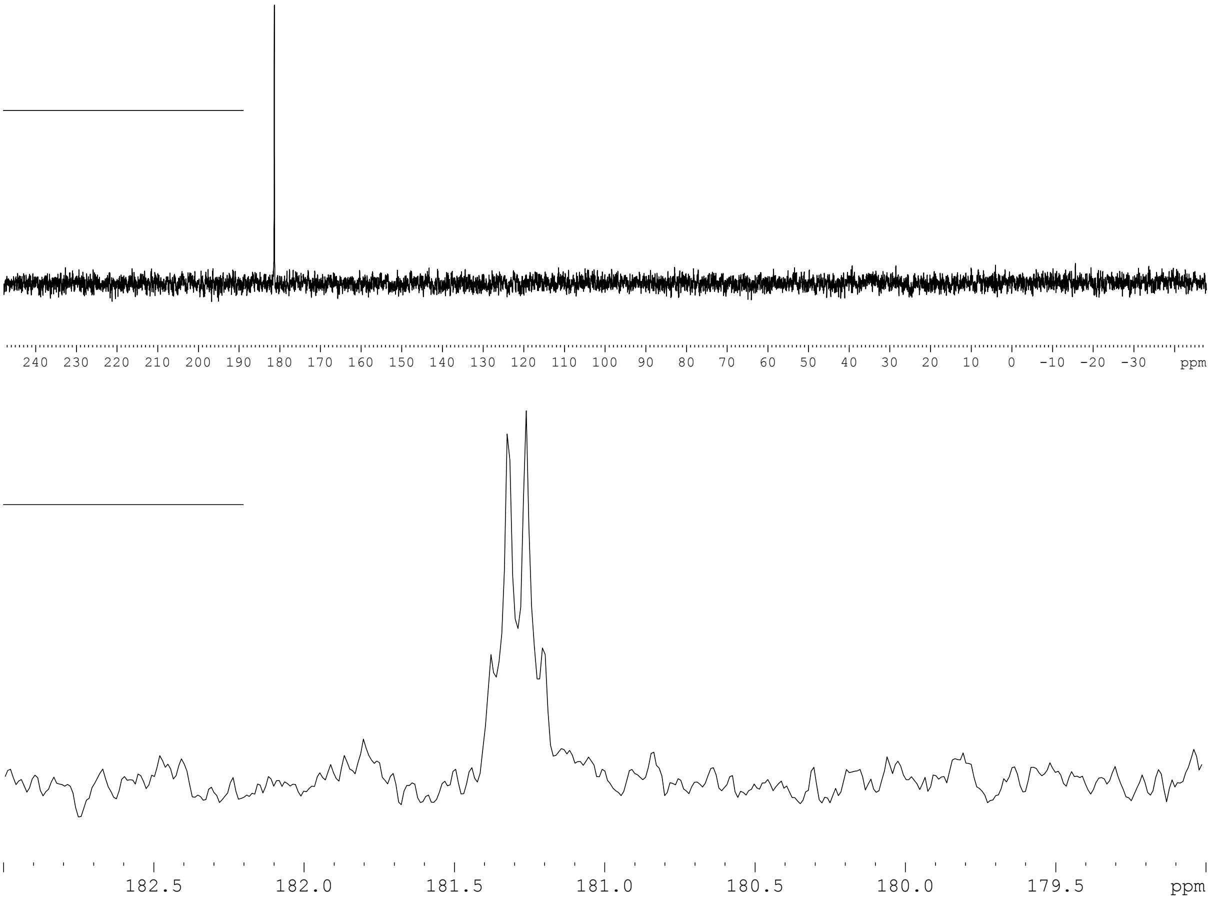

fig. S17d. ^{13}C SABRE-RELAY NMR spectrum for sodium acetate-1-^{13}C (top) and expansion (bottom).

Sodium pyruvate-1-^{13}C

fig. S18. SABRE-RELAY NMR spectra sodium pyruvate-^{13}C. (**a** to **b**) reflect a series of NMR spectra: (a) ^{13}C NMR spectrum thermally polarized (b) ^{13}C SABRE-RELAY NMR spectrum of sodium pyruvate-1-^{13}C using conditions: [IrCl(COD)(1,3-bis(2,4,6-trimethylphenyl)-4,5-dimethylimidazole)] (5 mM), sodium pyruvate-1-^{13}C (10 eq.),CD_2Cl_2 (0.6 mL), PEA (10 eq.), H_2O (10 µL); Substrate Concentration: 50 mM. ^{13}C enhancement (fold/per ^{13}C): 48 n.b. over a period of ca. 30 mins the Schiff base condensation between PEA and pyruvate is observed.

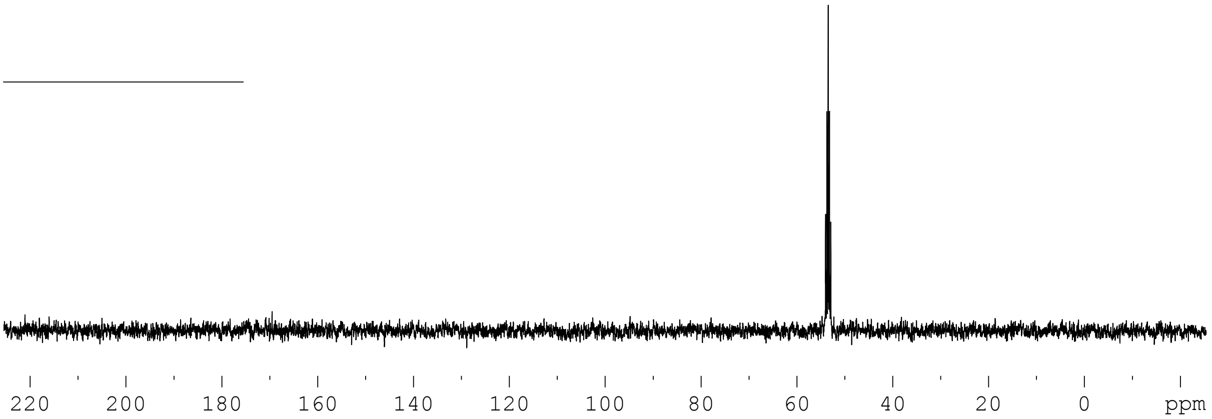

fig S18a. Thermally polarized ^{13}C NMR reference spectrum for sodium acetate-1-^{13}C.

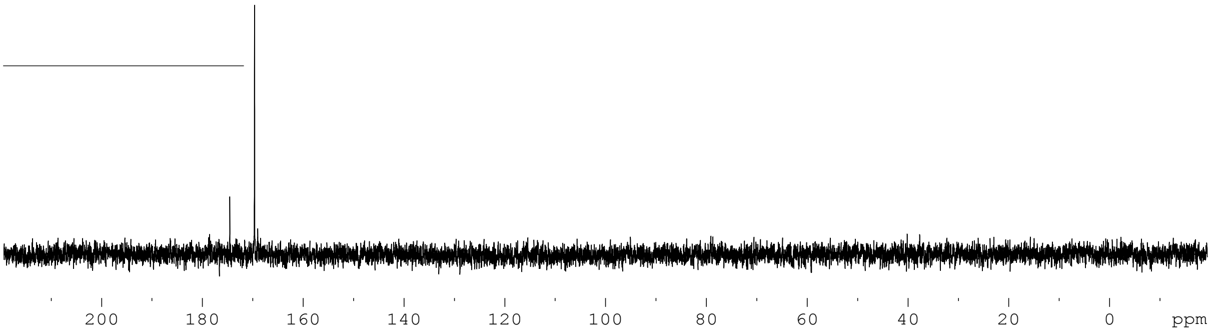

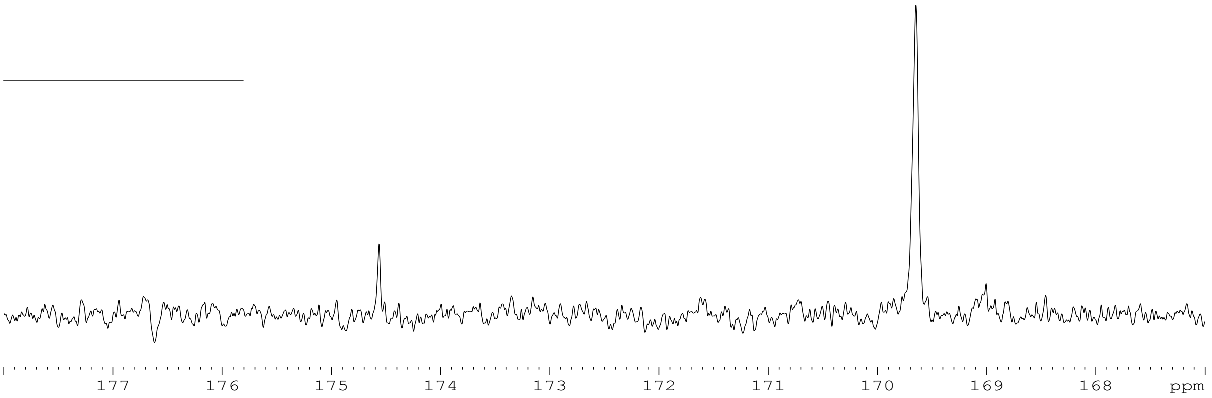

fig. S18b. ^{13}C SABRE-RELAY NMR spectrum for sodium pyruvate-1-^{13}C.

Sodium Acetate 1,2-$^{13}C_2$

fig. S19. SABRE-RELAY NMR spectra sodium acetate-1,2 ^{13}C2. (**a** to **b**) are the spectra for reflect a series of NMR spectra: (a) ^{13}C NMR spectrum thermally polarized and (b) ^{13}C SABRE-RELAY NMR spectrum for Sodium Acetate 1,2-$^{13}C_2$ using conditions: [IrCl(COD)(IMes)] (5 mM), sodium acetate 1,2-$^{13}C_2$ (10 eq.), CD_2Cl_2 (0.6 mL), NH_3, H_2O (10 µL); Substrate Concentration: 50 mM. ^{13}C enhancement (fold/per ^{13}C): 18 (<u>C</u>OOH), 4.5 (<u>C</u>H_3).

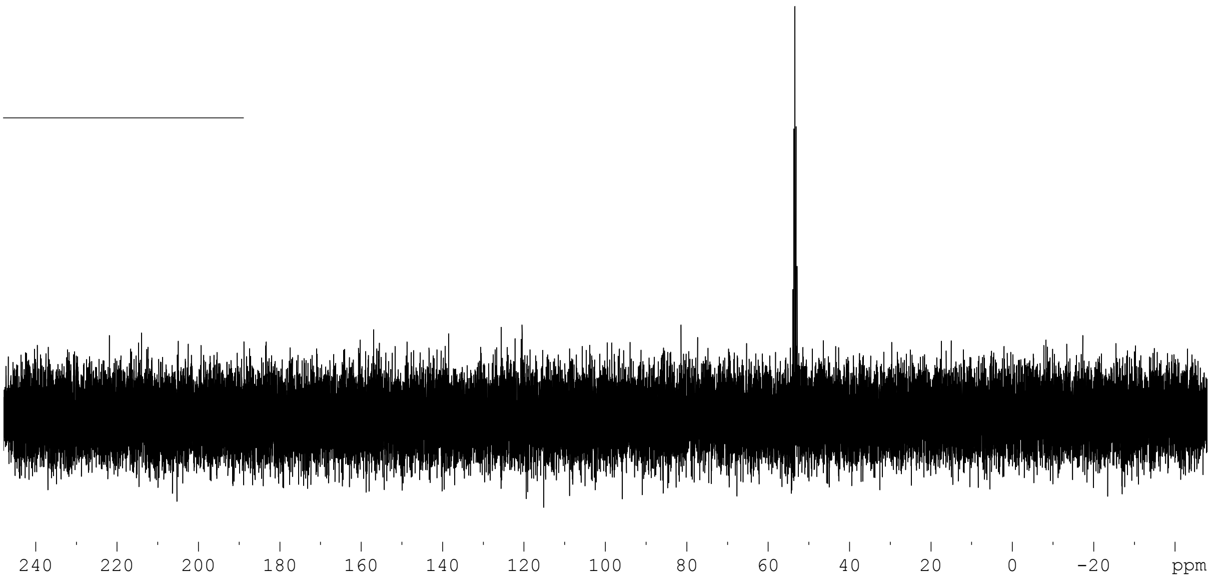

fig. S19a. Thermally polarized ^{13}C NMR reference spectrum for sodium acetate 1,2-$^{13}C_2$.

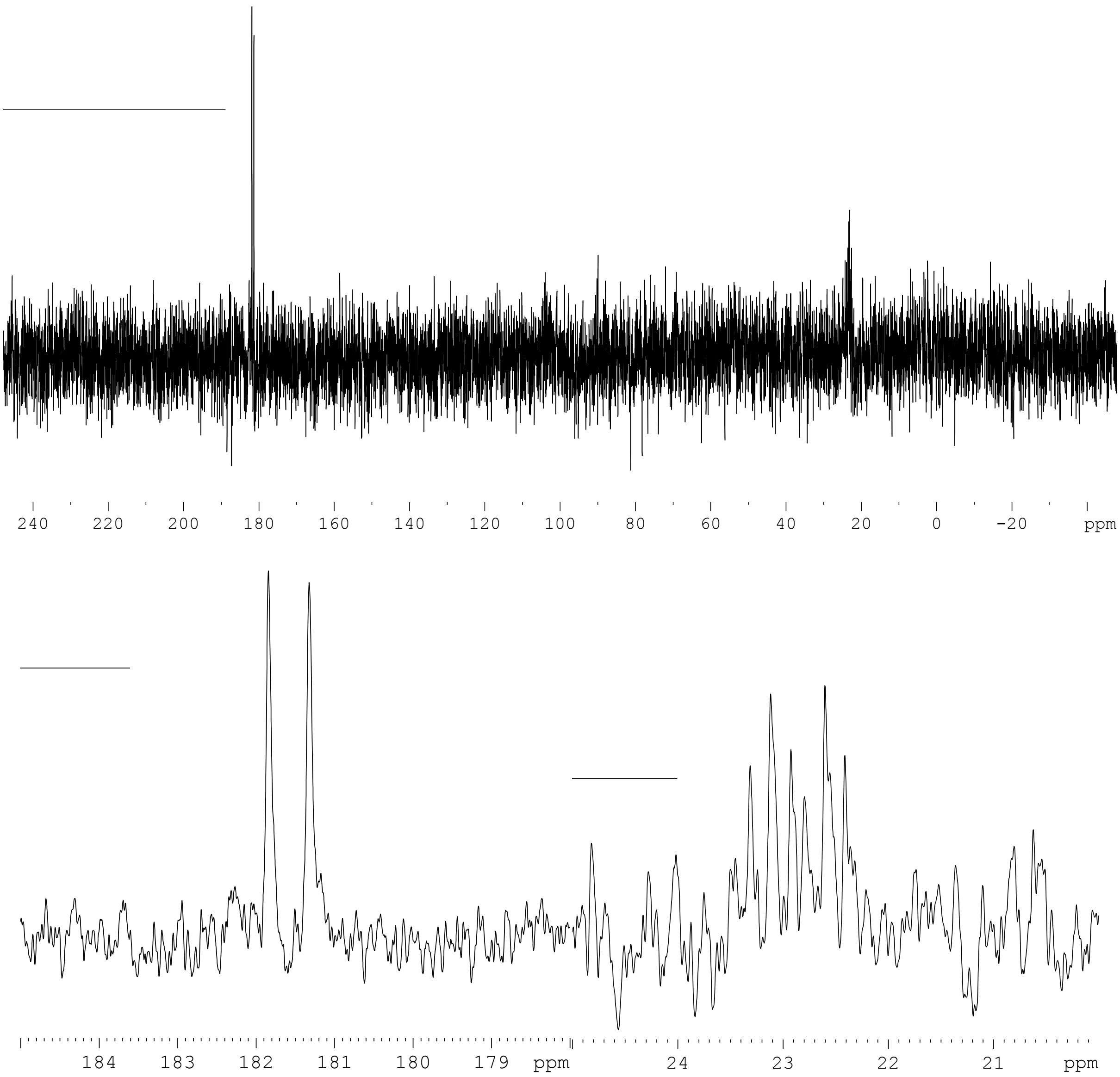

fig. S19b. ^{13}C SABRE-RELAY NMR spectrum for sodium acetate 1,2-$^{13}C_2$ (top) and expansions (bottom).

Propionic acid 1-^{13}C

fig. S20. SABRE-RELAY NMR spectra propionic acid-^{13}C. (**a** to **e**) reflect a series of single scan NMR spectra: (a) ^{1}H NMR spectrum thermally polarized, (b) ^{1}H SABRE-RELAY NMR spectrum, (c) ^{13}C NMR spectrum thermally polarized, (d) ^{13}C SABRE-RELAY NMR spectrum and (e) ^{13}C{^{1}H} SABRE-RELAYNMR spectrum for Propionic acid 1-^{13}C using conditions: [IrCl(COD)(IMes)] (5 mM), propionic acid 1-^{13}C (10 eq.), CD_2Cl_2 (0.6 mL), NH_3, H_2O (10 µL), Cs_2CO_3 (10 eq.); Substrate Concentration: 50 mM. ^{13}C enhancement (fold/per ^{13}C): 109.

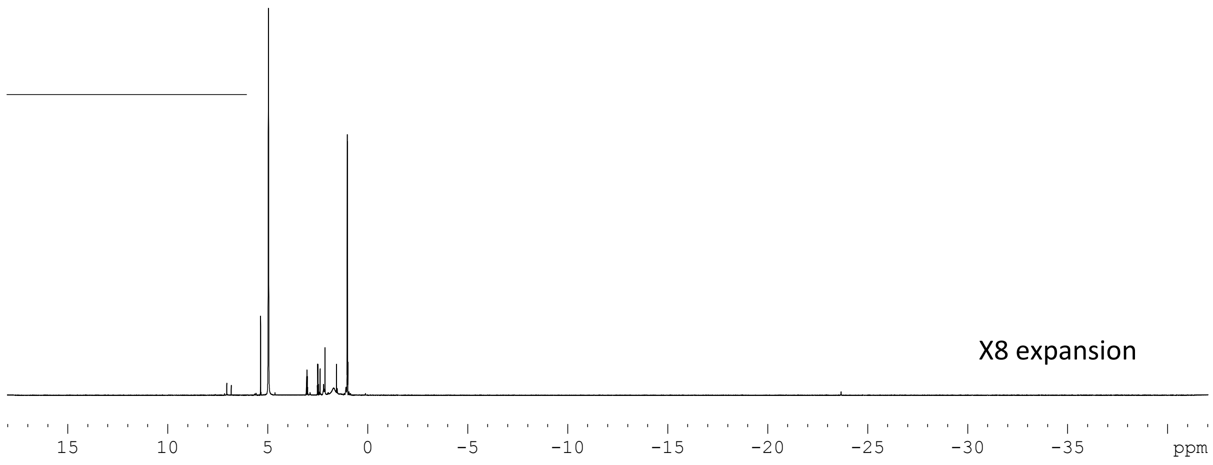

fig. S20a. Thermally polarized ^{1}H NMR reference spectrum for propionic acid 1-^{13}C.

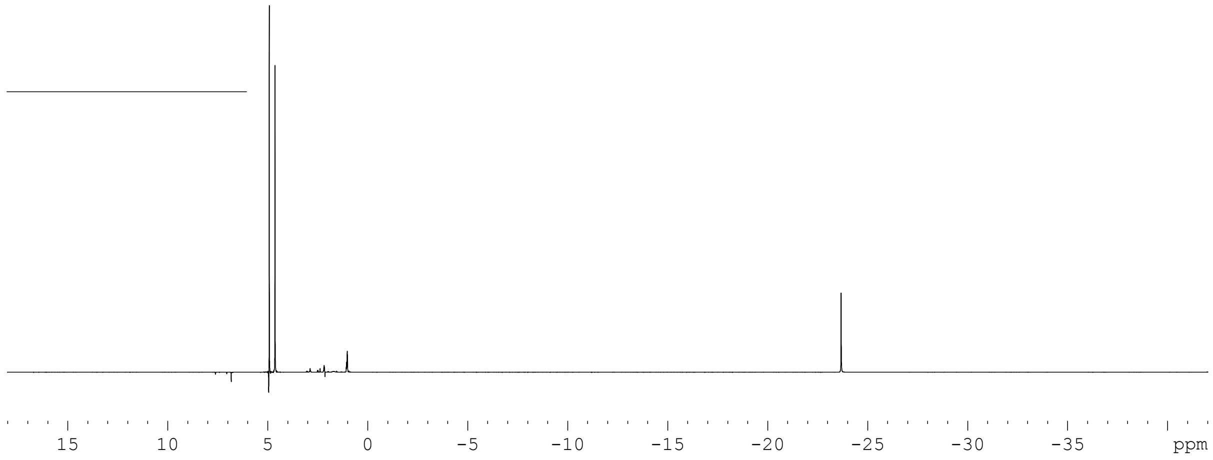

fig. S20b. ^{1}H SABRE-RELAY NMR spectrum for propionic acid 1-^{13}C

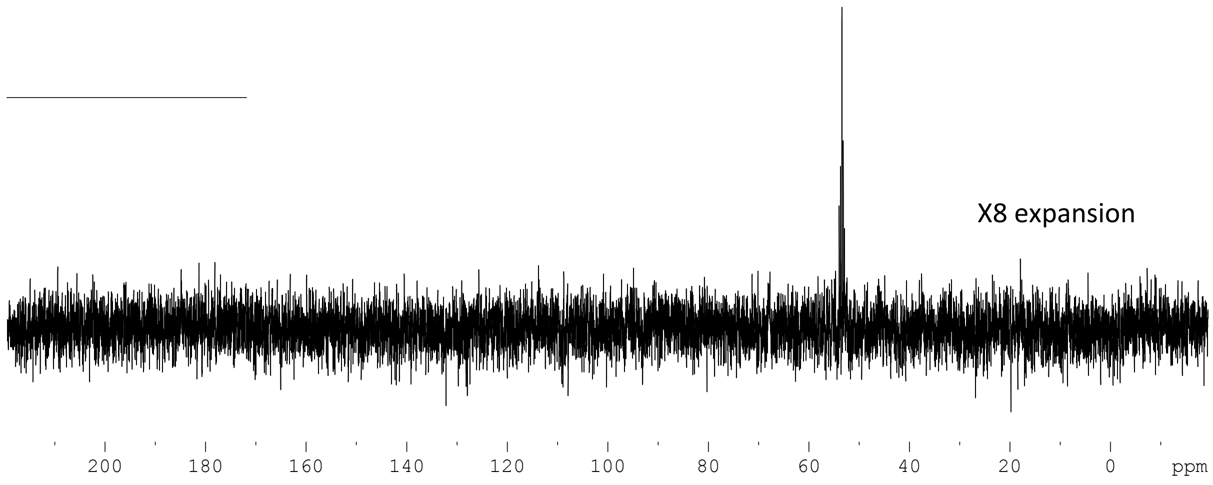

fig. S20c. Thermally polarized ^{13}C reference spectrum for propionic acid 1-^{13}C.

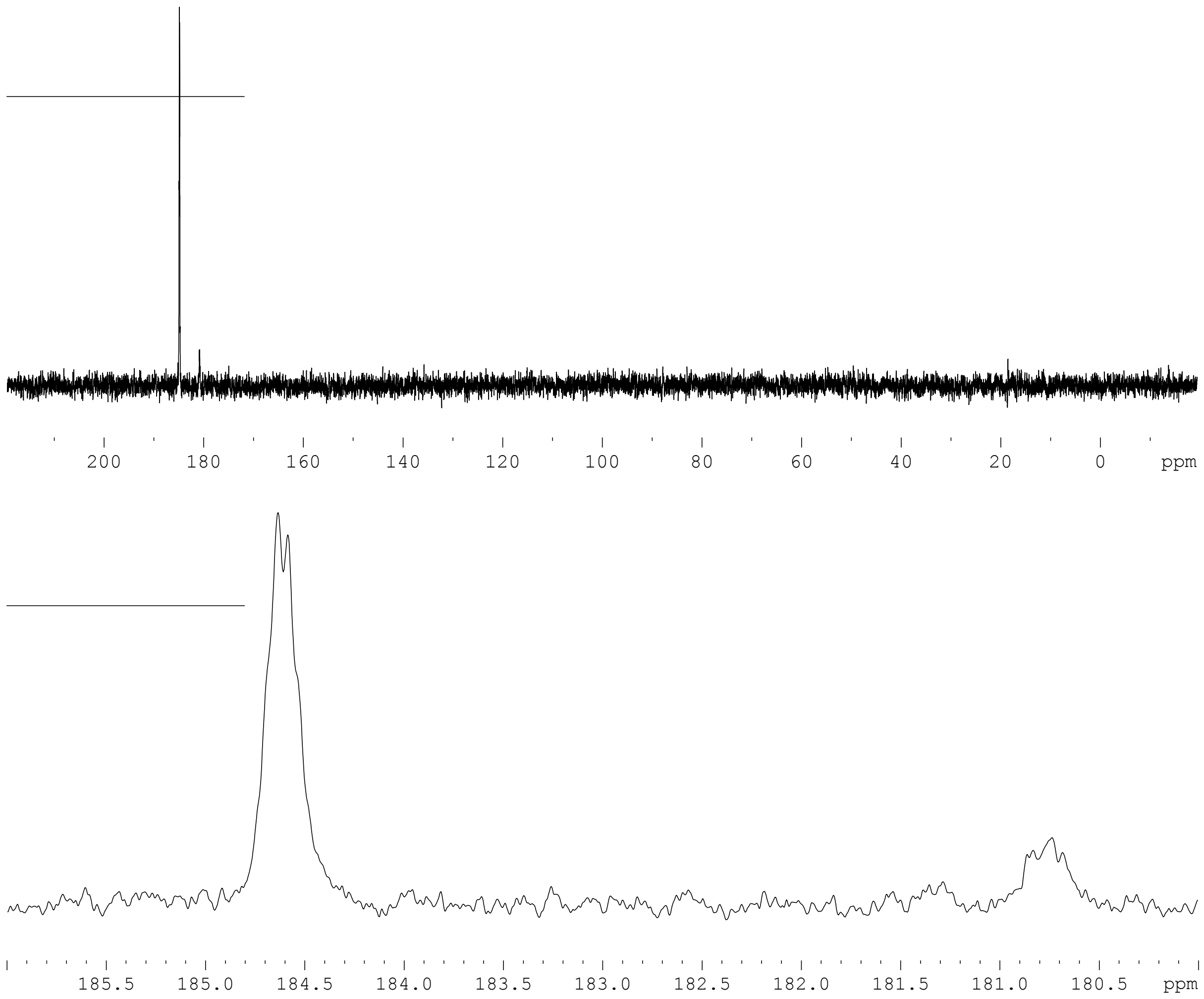

fig. S20d. ^{13}C SABRE-RELAY NMR spectrum for propionic acid 1-^{13}C (top) and expansion (bottom).

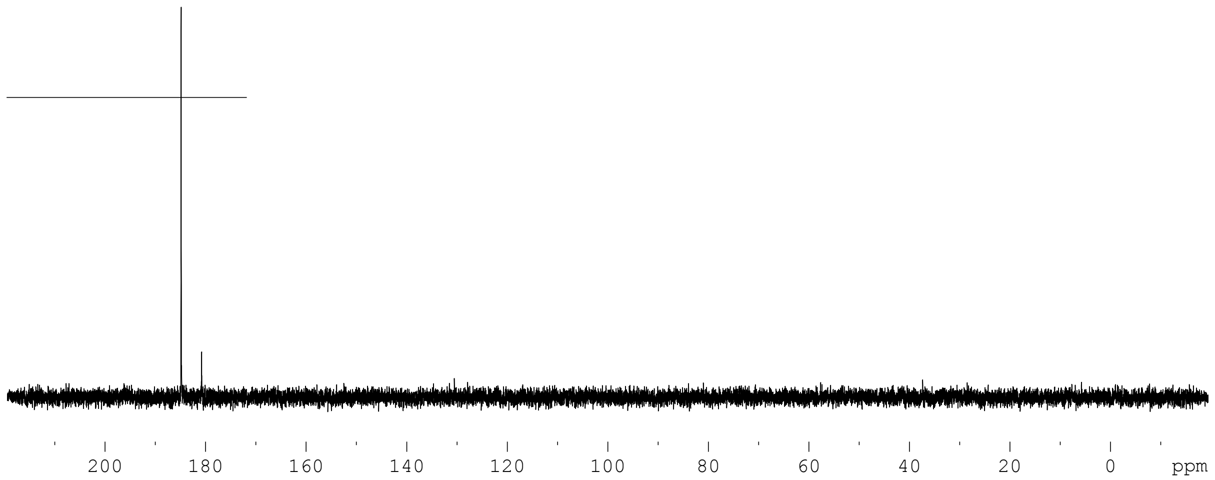

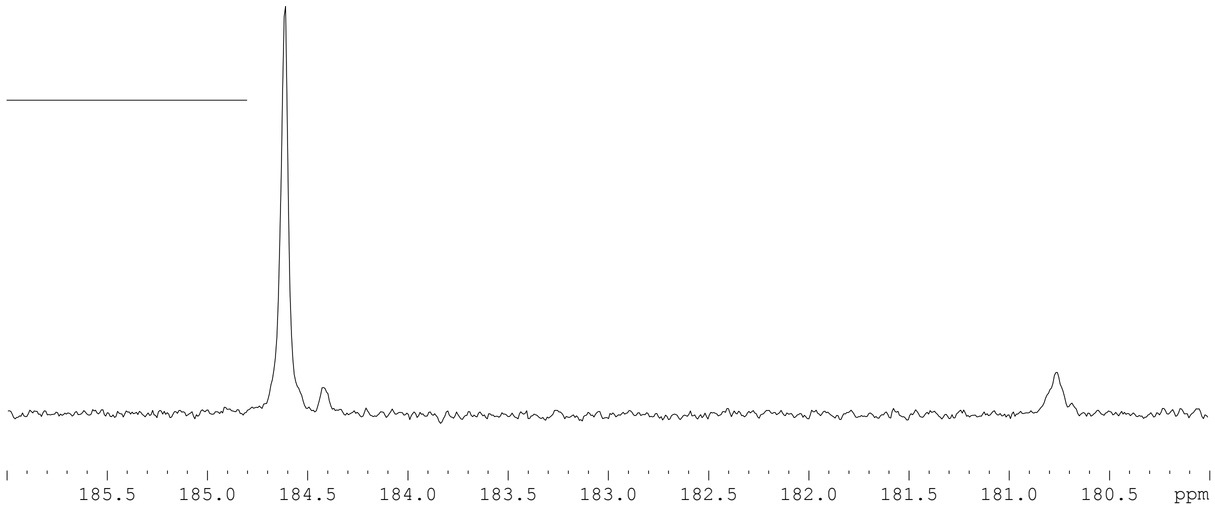

fig. S20e. $^{13}C\{^{1}H\}$ SABRE-RELAY NMR spectrum for propionic acid 1-^{13}C (top) and expansion (bottom).

Carbonates

Sodium hydrogen carbonate-^{13}C

fig. S21. SABRE-RELAY NMR spectra sodium hydrogen carbonate-^{13}C. (**a** to **b**) reflect a series of single scan NMR spectra: (a) ^{13}C NMR spectrum thermally polarized and (b) ^{13}C SABRE-RELAY NMR spectrum for sodium hydrogen carbonate-^{13}C using conditions: [IrCl(COD)(IMes)] (5 mM), sodium hydrogen carbonate-^{13}C (10 eq.), CD_2Cl_2 (0.6 mL), NH_3, H_2O (10 µL); Substrate Concentration: 50 mM. ^{13}C enhancement (fold/per ^{13}C): 11.

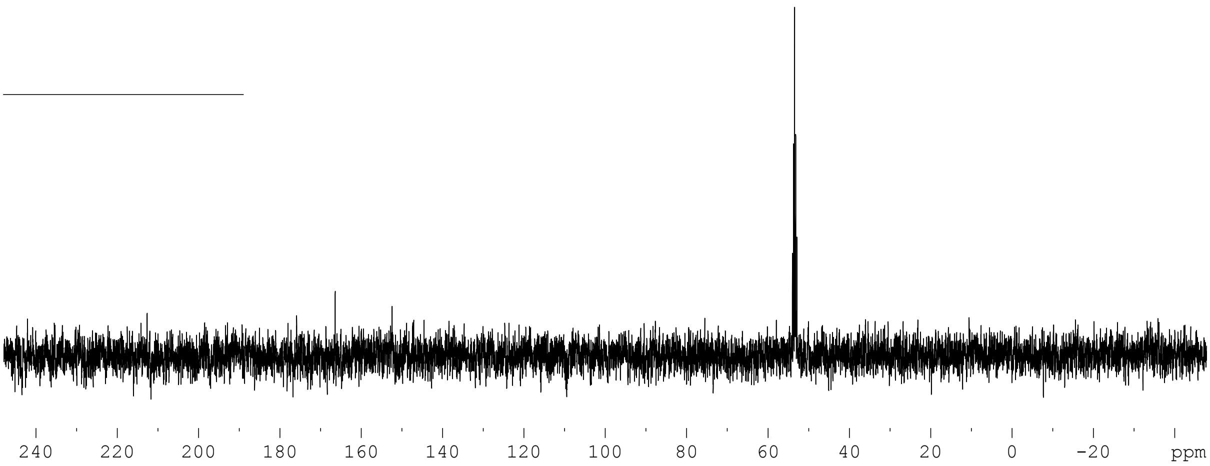

fig. S21a. Thermally polarized ^{13}C NMR reference spectrum for sodium hydrogen carbonate-^{13}C.

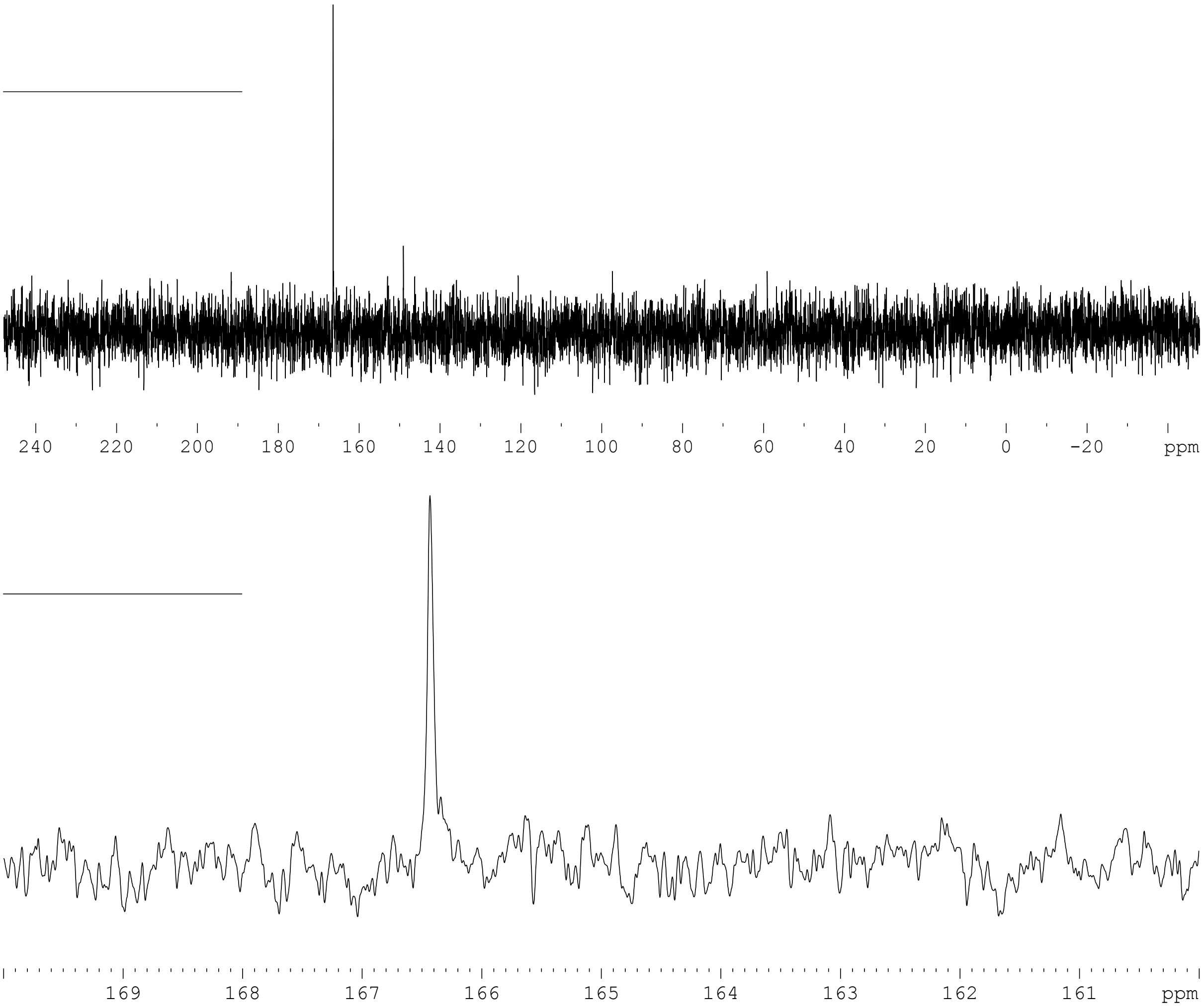

fig. S21b. ^{13}C SABRE-RELAY NMR spectrum for sodium hydrogen carbonate-^{13}C (top) and expansion (bottom).

Amides

Urea-^{13}C

fig. S22. SABRE-RELAY NMR spectra urea-^{13}C. (**a** to **d**) reflect a series of single scan NMR spectra: (a) ^{1}H NMR spectrum thermally polarized, (b) ^{1}H SABRE-RELAY NMR spectrum, (c) ^{13}C NMR spectrum thermally polarized and (d) ^{13}C SABRE-RELAY NMR spectrum for urea-^{13}C using conditions: [IrCl(COD)(IMes)] (5 mM), Urea-1-^{13}C (10 eq.),CD_2Cl_2 (0.6 mL), NH_3 and H_2O (5 µL); Substrate Concentration: 50 mM. ^{1}H enhancement (fold/per proton): 125 (NH). ^{13}C enhancement (fold/per ^{13}C): 182.

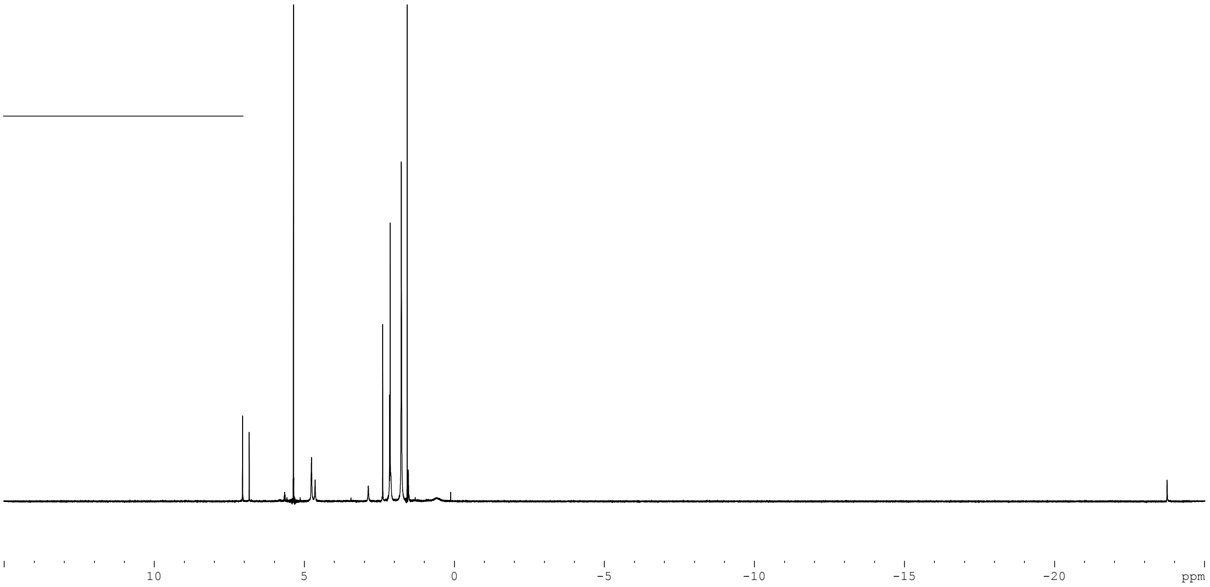

fig. S22a. Thermally polarized ^{1}H NMR reference spectrum for urea-^{13}C.

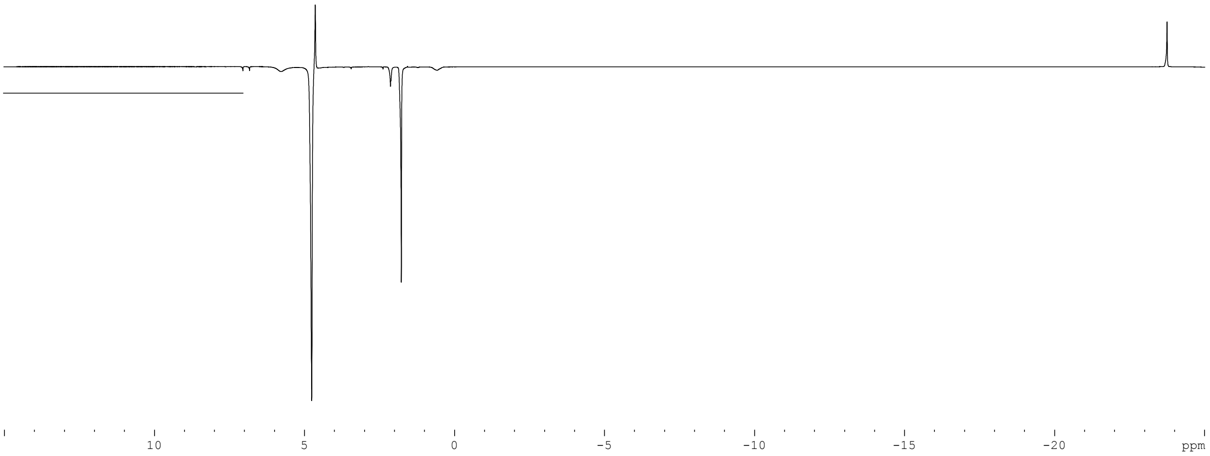

fig. S22b. ^{1}H SABRE-RELAY NMR spectrum for urea-^{13}C.

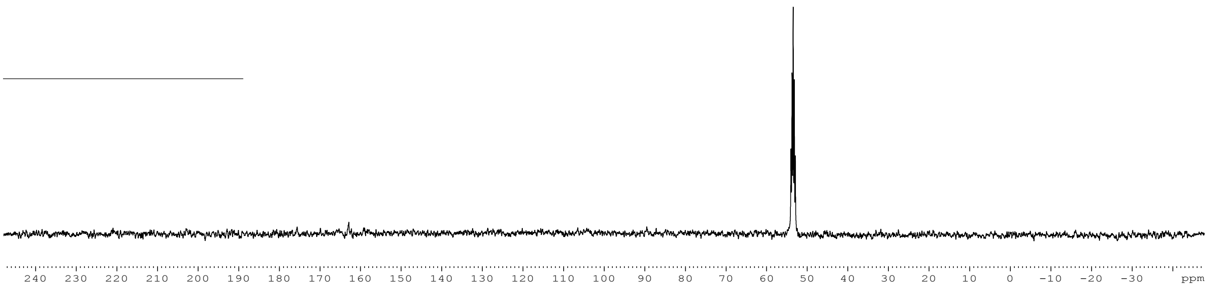

fig. S22c. Thermally polarized ^{13}C NMR reference spectrum for for urea-^{13}C.

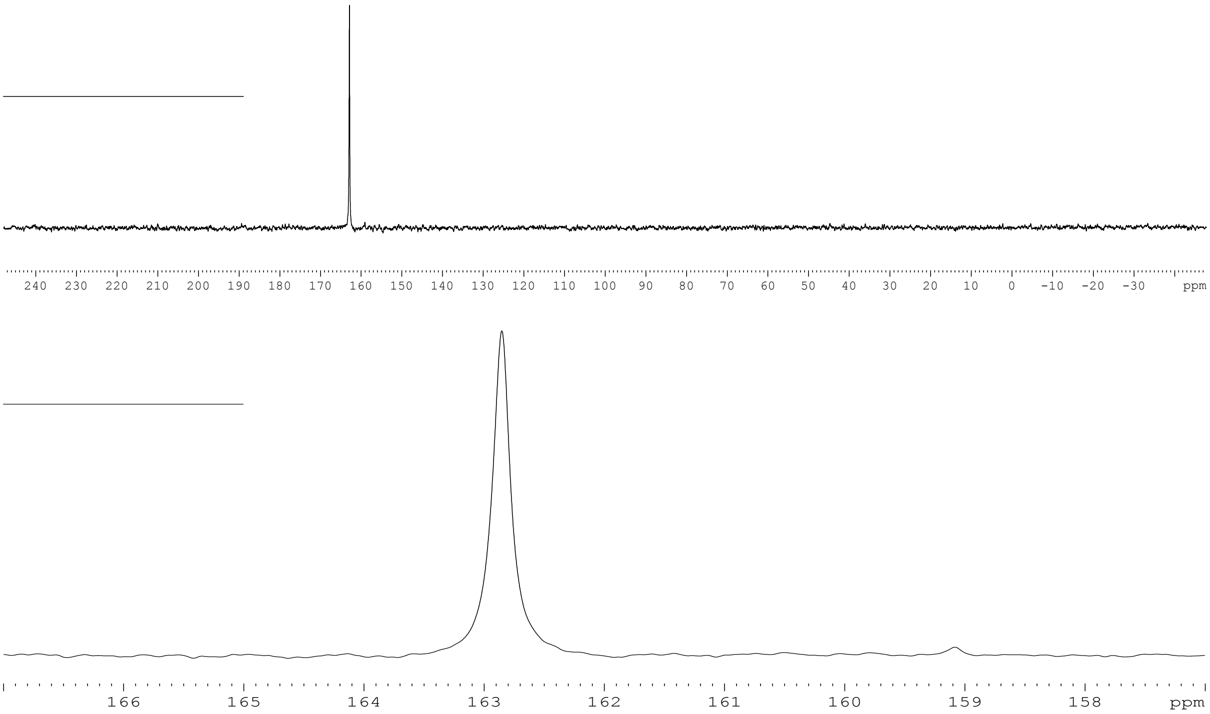

fig. S22d. ^{13}C SABRE-RELAY NMR spectrum for urea-^{13}C (top) expansion (bottom).

Urea-^{13}C-^{15}N$_2$

fig. S23. SABRE-RELAY NMR spectra urea-^{13}C-^{15}N2. (**a** to **e**) reflect a series of single scan NMR spectra: (a) ^{13}C NMR spectrum thermally polarized, (b) ^{13}C SABRE-RELAY NMR spectrum, (c) ^{15}N NMR spectrum thermally polarized, (d) ^{15}N SABRE-RELAY NMR spectrum for urea-^{13}C-$^{15}N_2$, (e) $^{15}N\{^1H\}$SABRE-RELAY NMR spectrum for urea-^{13}C-$^{15}N_2$using conditions: [IrCl(COD)(1,3-bis(2,4,6-trimethylphenyl)-4,5-dimethylimidazole)] (5 mM), Urea-^{13}C-$^{15}N_2$ (5 eq.),CD_2Cl_2 (0.6 mL), PEA (1 µL) , H_2O (5 µL); Substrate Concentration: 25 mM. ^{13}C enhancement (fold/per ^{13}C): 408.

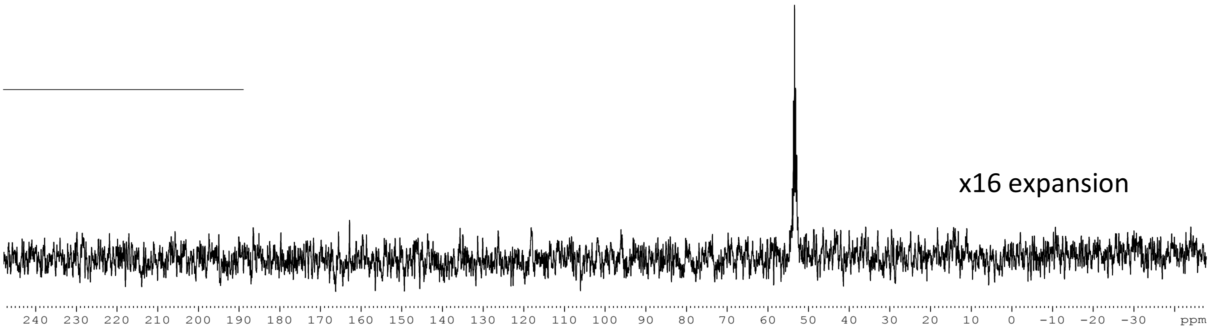

fig. S23a. Thermally polarized ^{13}C NMR reference spectrum for for urea-^{13}C-$^{15}N_2$.

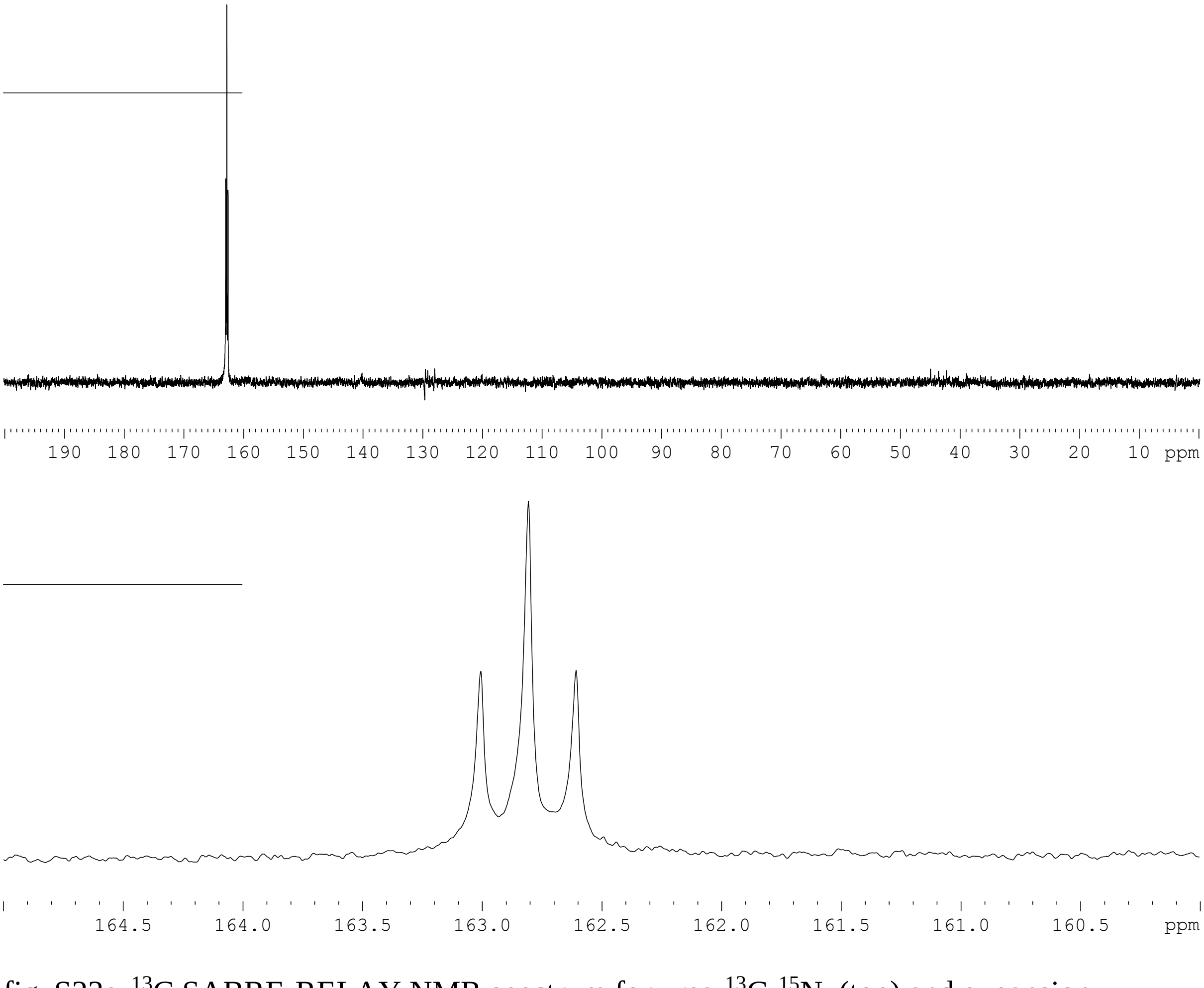

fig. S23e. ^{13}C SABRE-RELAY NMR spectrum for urea-^{13}C-$^{15}N_2$ (top) and expansion (bottom).

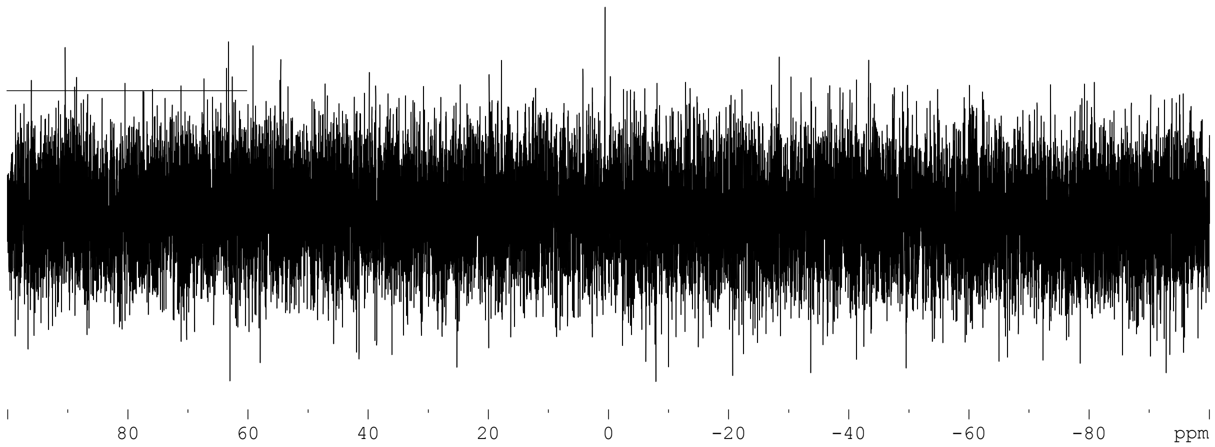

fig. S23c. Thermally polarized ^{15}N spectrum for urea-^{13}C-$^{15}N_2$.

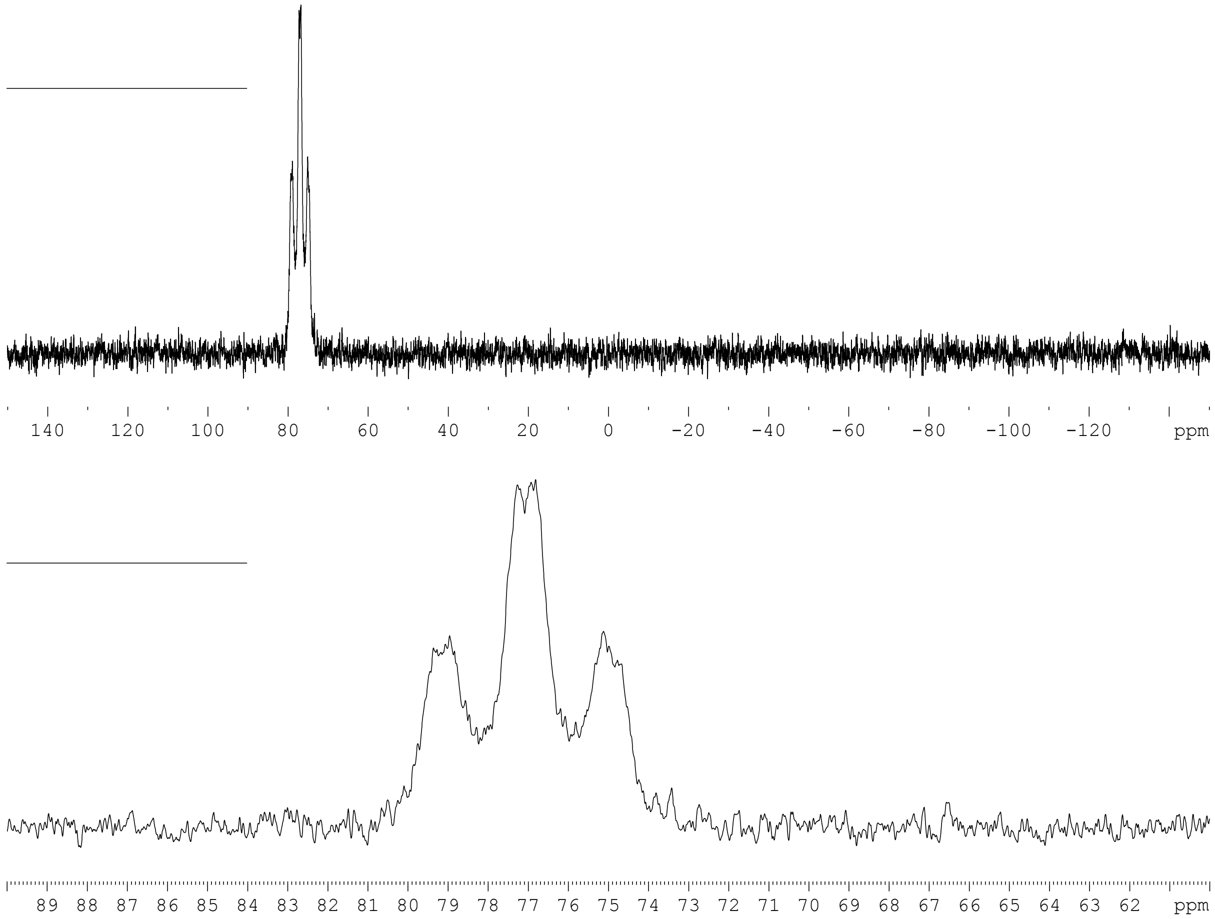

fig. S23d. ^{15}N SABRE-RELAY NMR spectrum for urea-^{13}C-$^{15}N_2$ (top) and expansion (bottom).

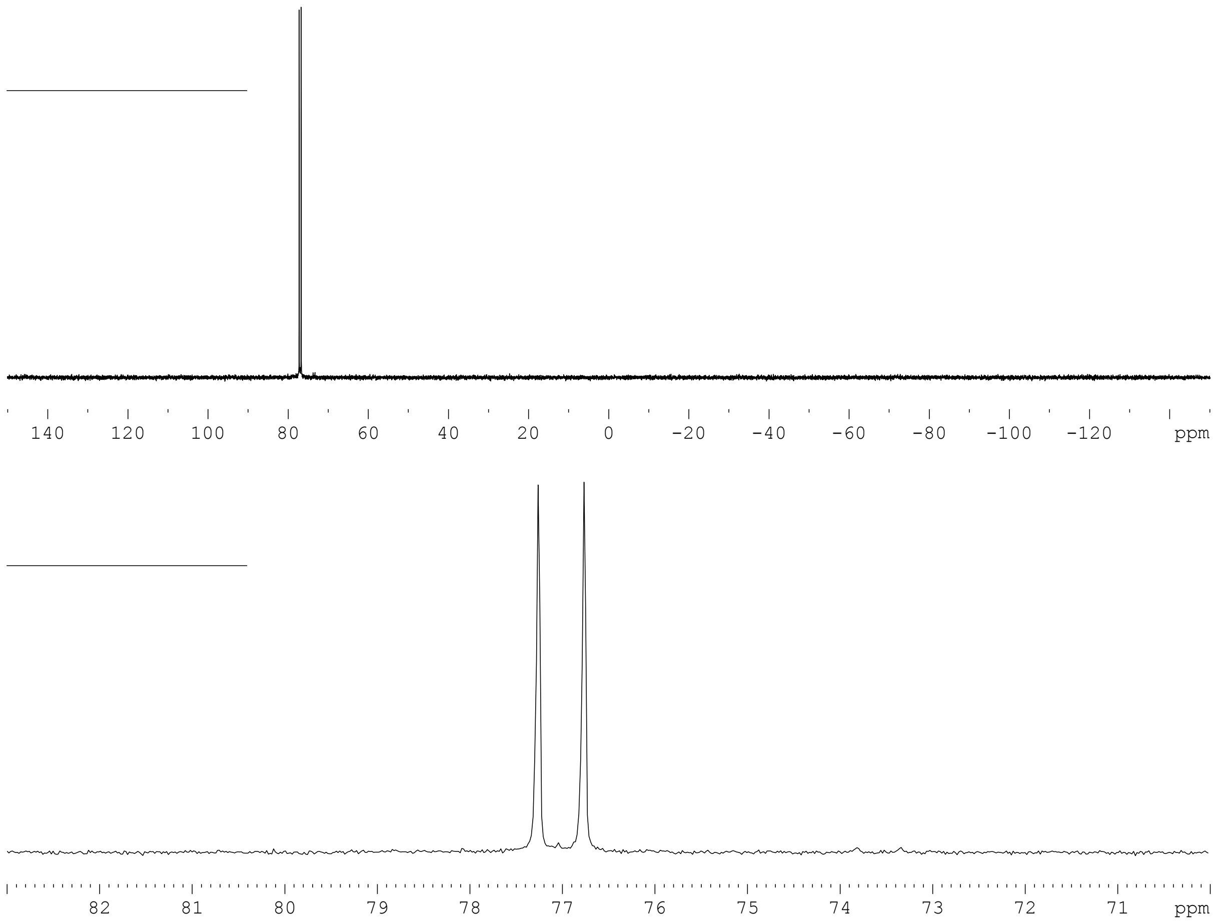

fig. S23e. $^{15}N\{^{1}H\}$ SABRE-RELAY NMR spectrum for urea-^{13}C-$^{15}N_2$ (top) and expansion (bottom).

fig. S24. SABRE-RELAY NMR spectra urea-^{13}C-^{15}N2. (**a** to **b**) reflect a series of single scan NMR spectra at reduced concentration: (a) ^{13}C NMR spectrum thermally polarized, (b) ^{13}C SABRE-RELAY NMR spectrum using conditions: [IrCl(COD)(1,3-bis(2,4,6-trimethylphenyl)-4,5-dimethylimidazole)] (5 mM), Urea-^{13}C-$^{15}N_2$ (1 eq.),CD_2Cl_2 (0.6 mL), PEA (1 µL) , H_2O (5 µL); Substrate Concentration: 5 mM.

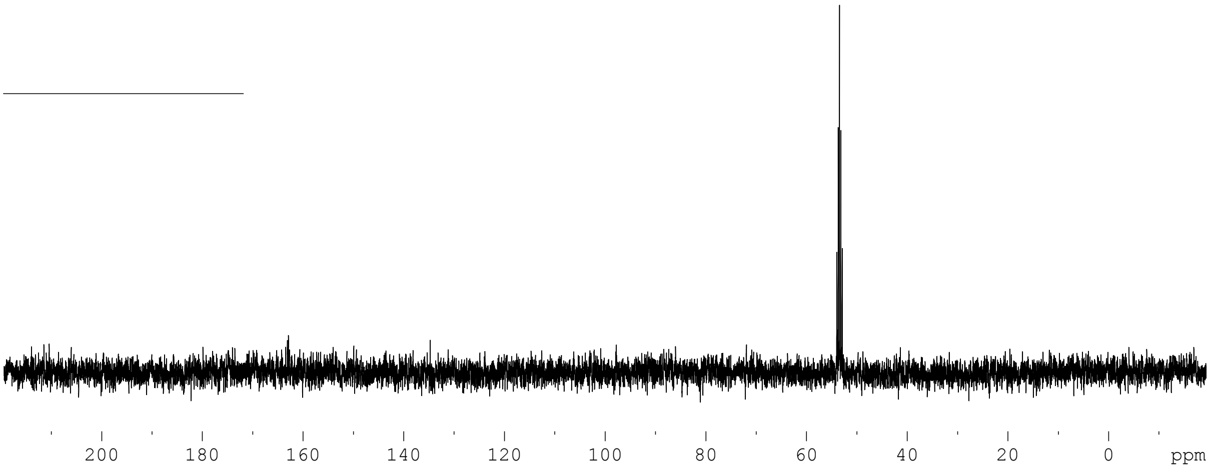

fig. S24a. Thermally polarized ^{13}C NMR reference spectrum for urea-^{13}C-$^{15}N_2$ at reduced concentration.

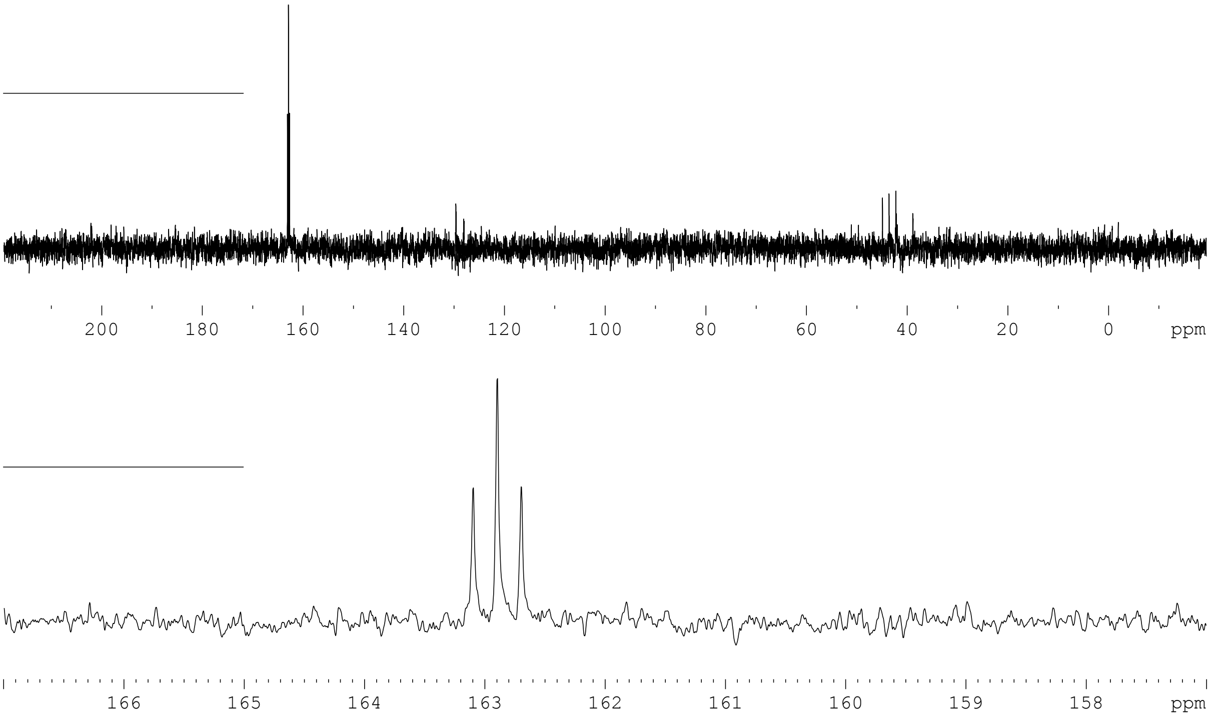

fig. S24b. ^{13}C SABRE-RELAY NMR spectrum for urea-^{13}C-$^{15}N_2$ at reduced concentration (top) and expansion (bottom).

Acetamide

fig. S25. SABRE-RELAY NMR spectra acetamide. (**a** to **b**) reflect a series of single scan NMR spectra: (a) ^{1}H NMR spectrum Thermally polarized, (b) ^{1}H SABRE-RELAY NMR spectrum, for acetamide using conditions: [IrCl(COD)(IMes)] (5 mM), acetmide (10 eq.),CD_2Cl_2 (0.6 mL), NH_3, D_2O (1 µL); Substrate Concentration: 50 mM. ^{1}H enhancement (fold/per proton): 248 (NH), 166 (CH_3). The NH_2 group of acetamide returned a 170-fold signal gain per proton while its CH_3 groups signal gain was 80-fold per proton, and the added water return 130-fold per proton.

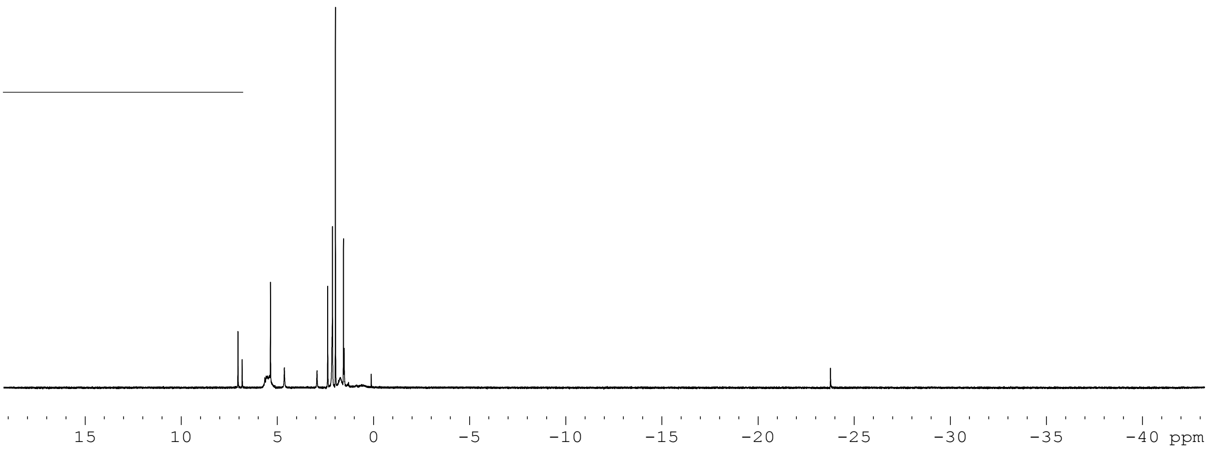

fig. S25a. Thermally polarized ^{1}H NMR reference spectrum for acetamide.

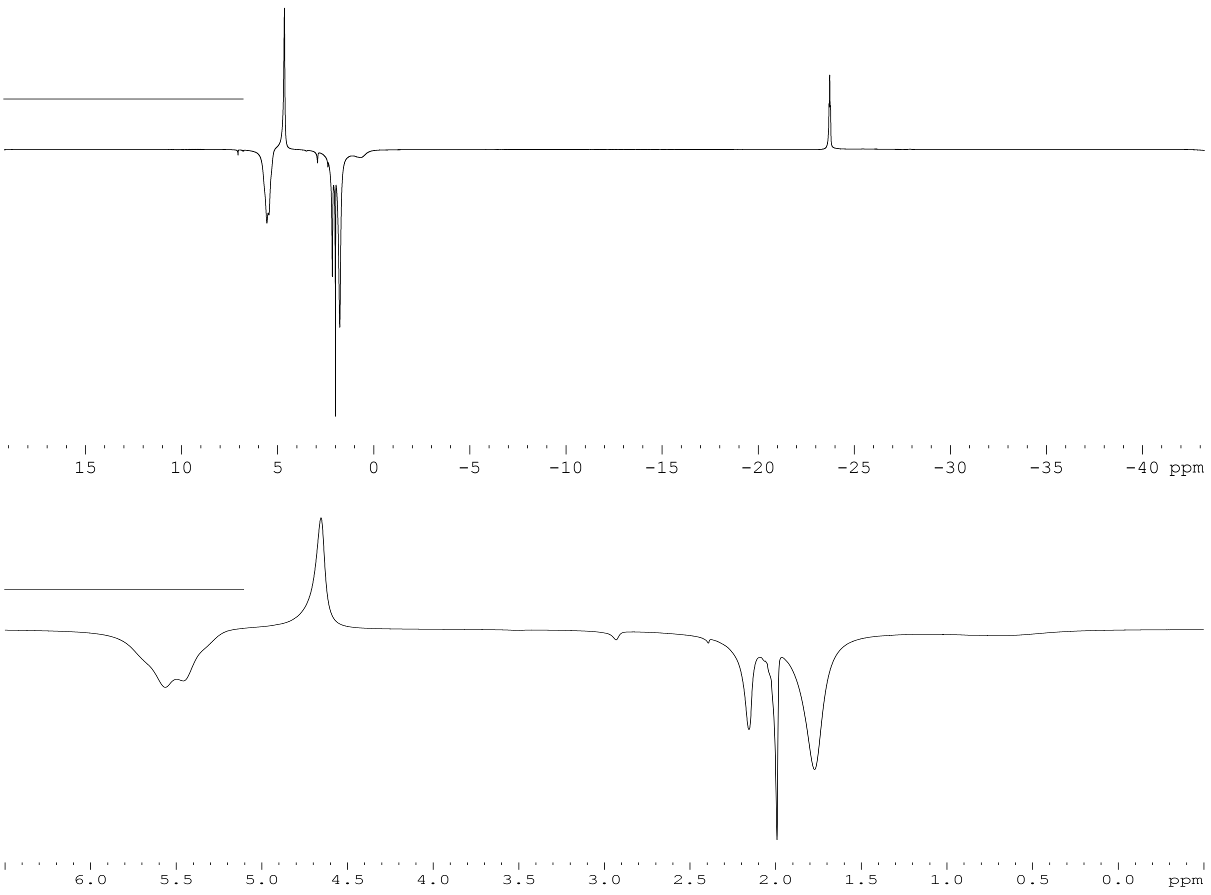

fig. S25a. ^{1}H SABRE-RELAY NMR spectrum for acetamide (top) and expansion (bottom).

Methacrylamide

fig. S26. SABRE-RELAY NMR spectra methacrylamide. (**a** to **b**) reflect a series of single scan NMR spectra: (a) ^{1}H NMR spectrum Thermally polarized, (b) ^{1}H SABRE-RELAY NMR spectrum, for methacrylamide using conditions: [IrCl(COD)(IMes)] (5 mM), methacrylamide (10 eq.),CD_2Cl_2 (0.6 mL), NH_3; Substrate Concentration: 50 mM. ^{1}H enhancement (fold/per proton): 84 (NH), 58 (=CH_2), 26 (CH_3).

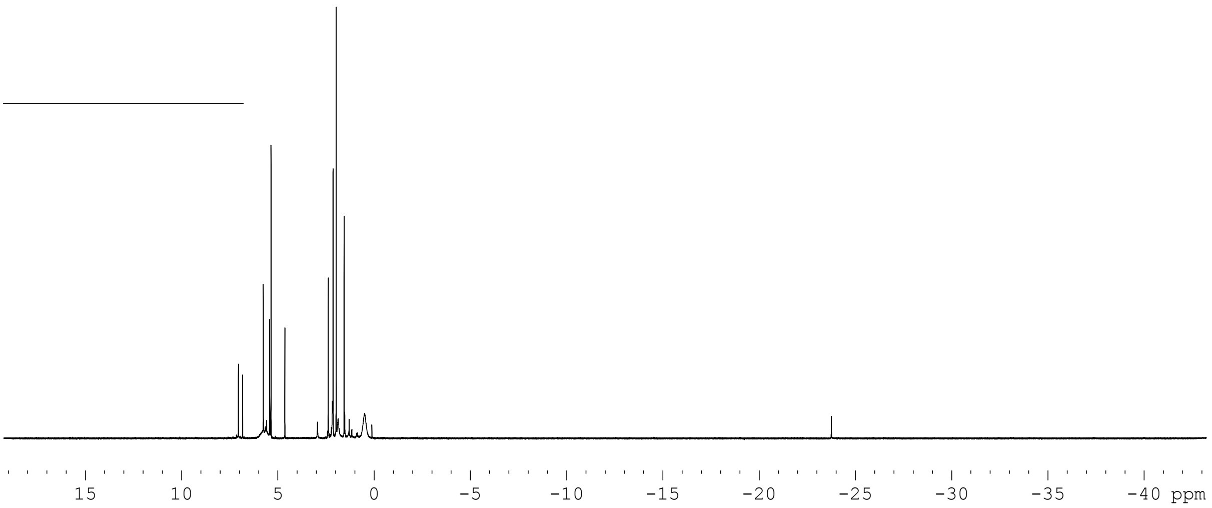

fig. S26a. Thermally polarized ^{1}H NMR reference spectrum for methacrylamide.

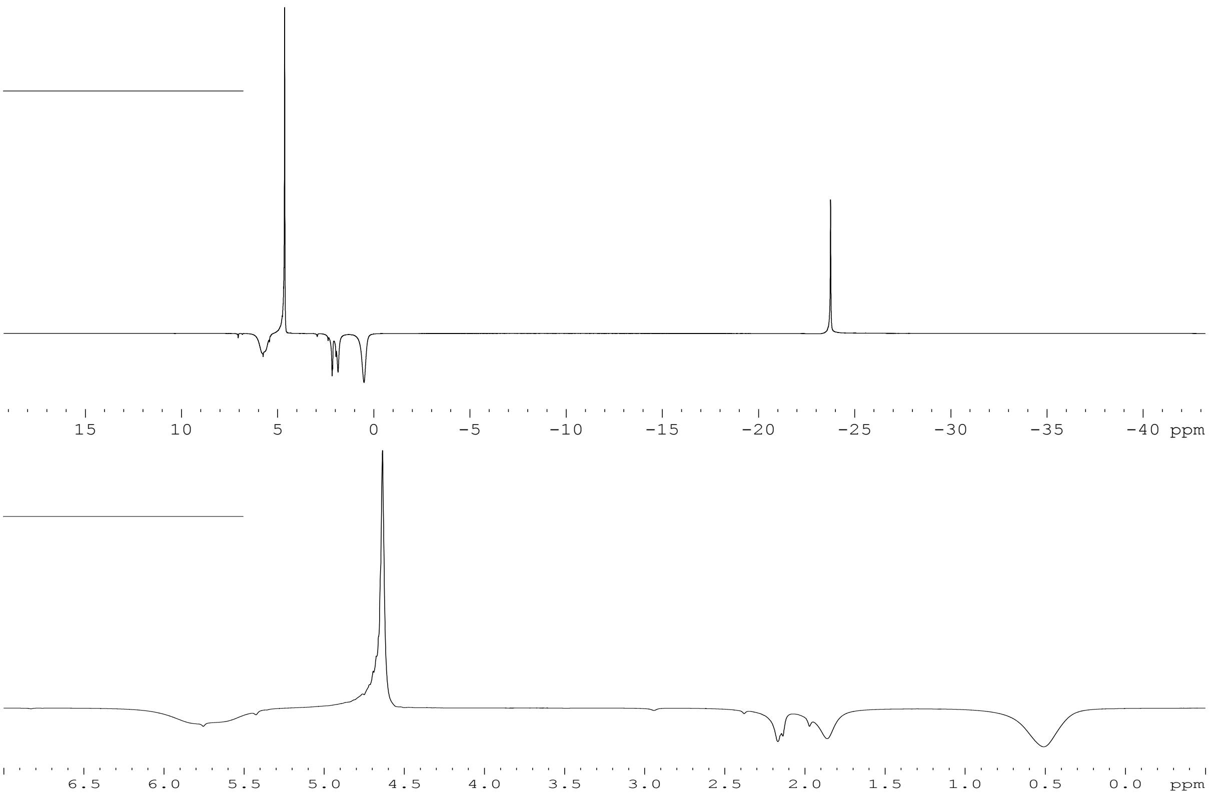

fig. S26b. ^{1}H SABRE-RELAY NMR spectrum for methacrylamide (top) and expansion (bottom).

Cyclohexyl methacrylamide

fig. S27. SABRE-RELAY NMR spectra cyclohexyl methacrylamide. (**a** to **b**) reflect a series of single scan NMR spectra: (a) ^{1}H NMR spectrum Thermally polarized, (b) ^{1}H SABRE-RELAY NMR spectrum, for cyclohexyl methacrylamide using conditions:

[IrCl(COD)(IMes)] (5 mM), cyclohexyl methacrylamide (10 eq.),CD_2Cl_2 (0.6 mL), NH_3, D_2O (0.7 µL); Substrate Concentration: 50 mM. ^{1}H enhancement (fold/per proton): 131 (NH).

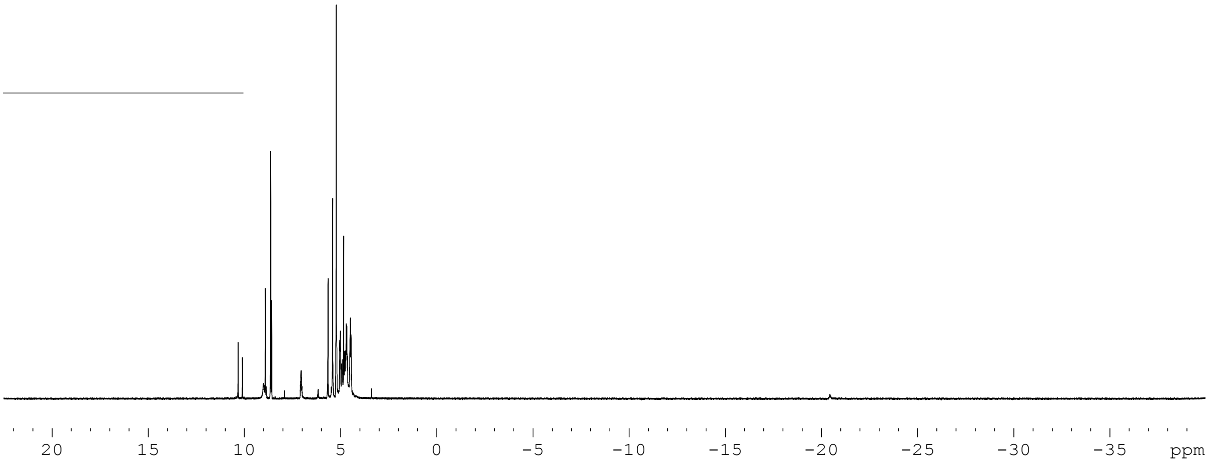

fig. S27a. Thermally polarized ^{1}H NMR reference spectrum for cyclohexyl methacrylamide.

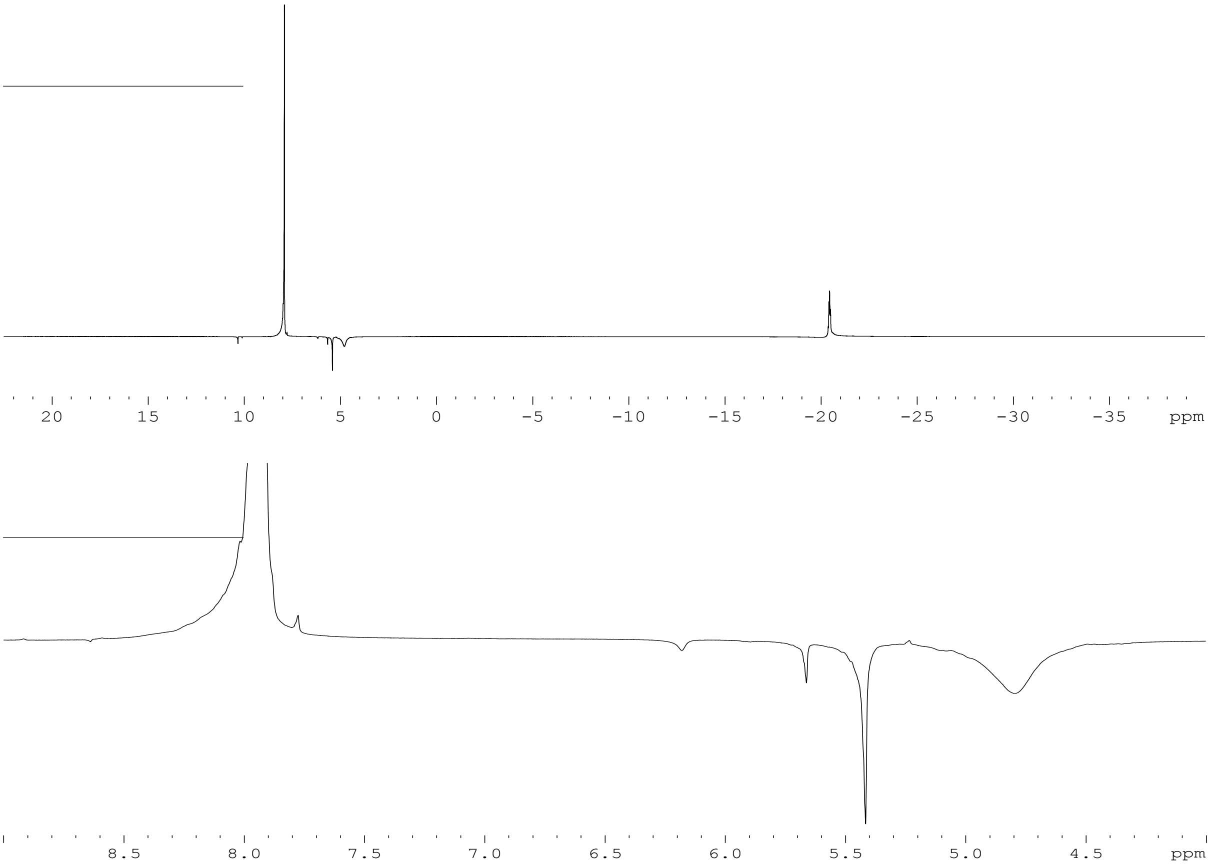

fig. S27b. ^{1}H SABRE-RELAY NMR spectrum for cyclohexyl methacrylamide (top) and expansion (bottom).

Phosphates

Mono sodium dihydrogen orthphosphate

fig. S28. SABRE-RELAY NMR spectra mono sodium dihydrogen orthophosphate. (**a** to **e**) reflect a series of single scan NMR spectra: (a) ^{1}H NMR spectrum thermally polarized, (b) ^{1}H SABRE-RELAY NMR spectrum, (c) ^{31}P NMR spectrum thermally polarized , (d) ^{31}P NMR spectrum SABRE-RELAY and (e) ^{31}P SABRE-RELAY refocused INEPT NMR spectrum for mono sodium dihydrogen orthophosphate using conditions: [IrCl(COD)(IMes)] (5 mM), mono sodium dihydrogen orthophosphate (10 eq.),CD_2Cl_2 (0.6 mL), NH_3, H_2O (10 µL); Substrate Concentration: 50 mM. ^{31}P enhancement (fold/per ^{31}P nuclei): 226.

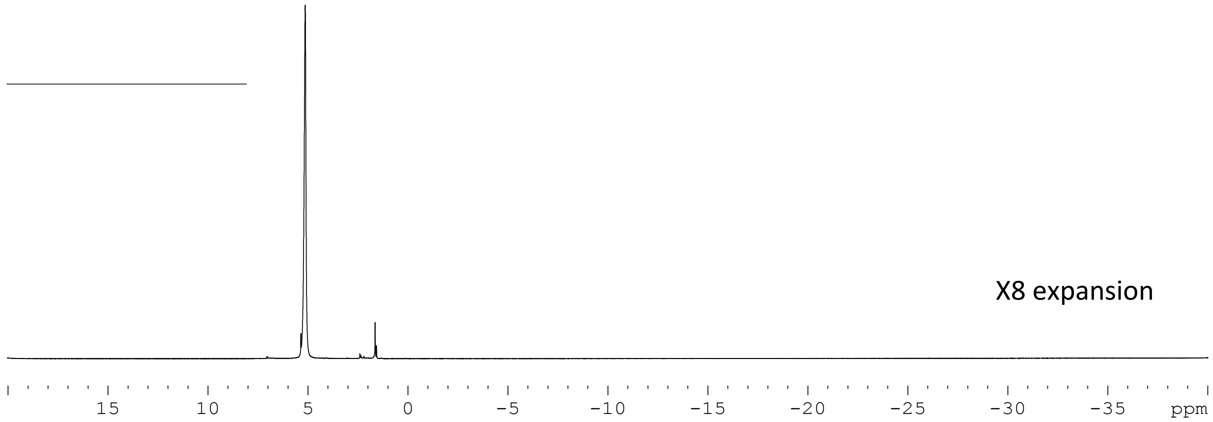

fig. S28a. Thermally polarized ^{1}H NMR reference spectrum for mono sodium dihydrogen orthophosphate.

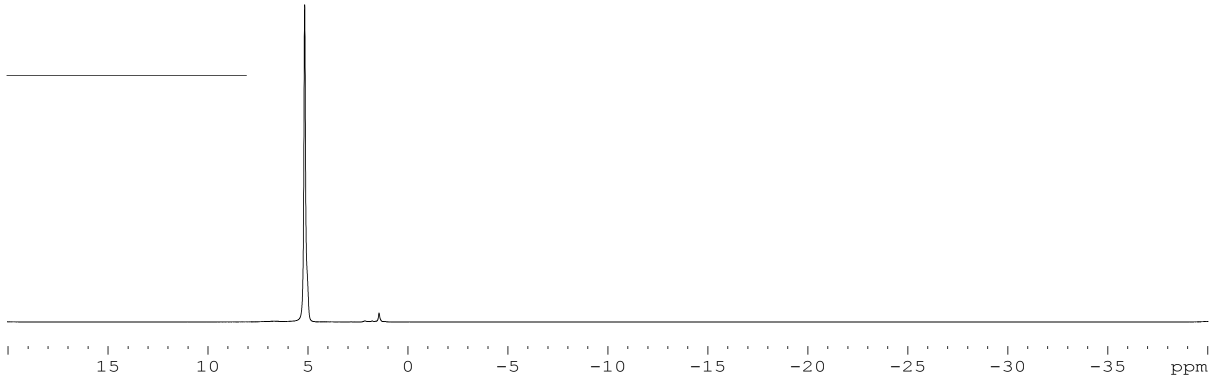

fig. S28b. ^{1}H SABRE-RELAY NMR spectrum for mono sodium dihydrogen orthophosphate.

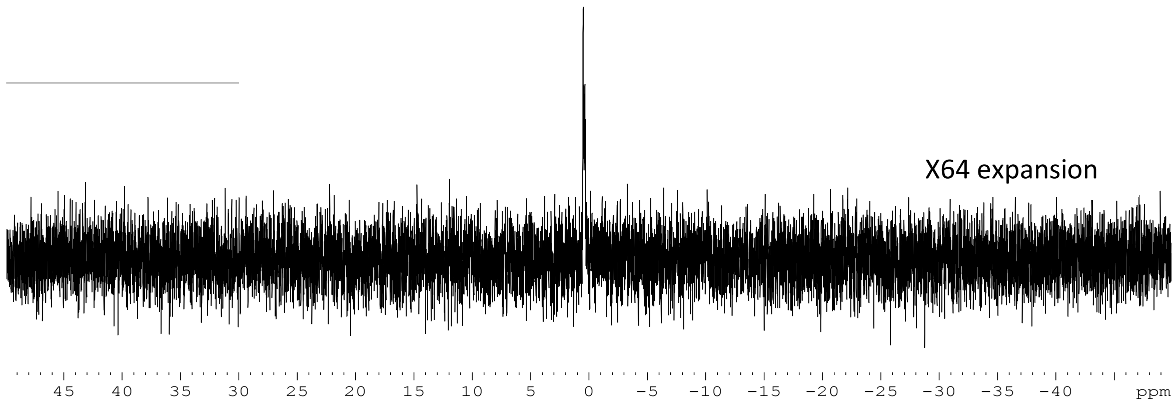

fig. S28c. Thermally polarized ^{31}P NMR reference spectrum for mono sodium dihydrogen orthophosphate.

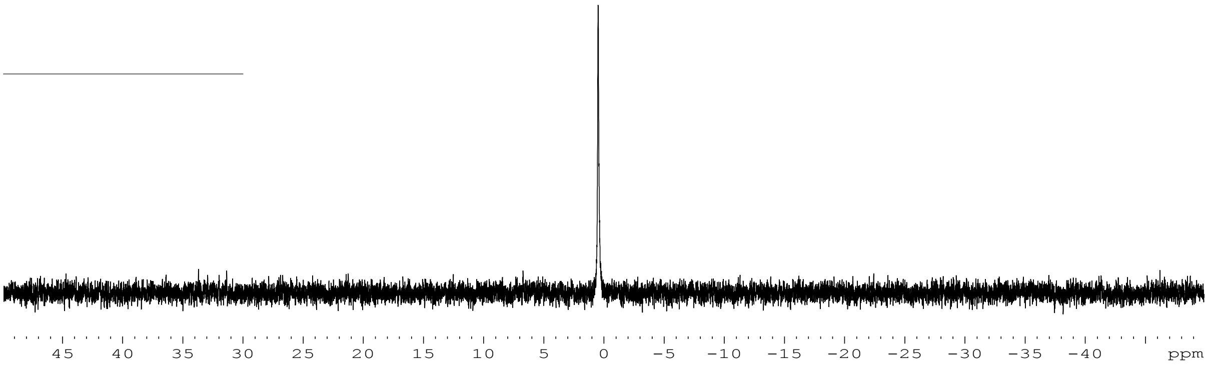

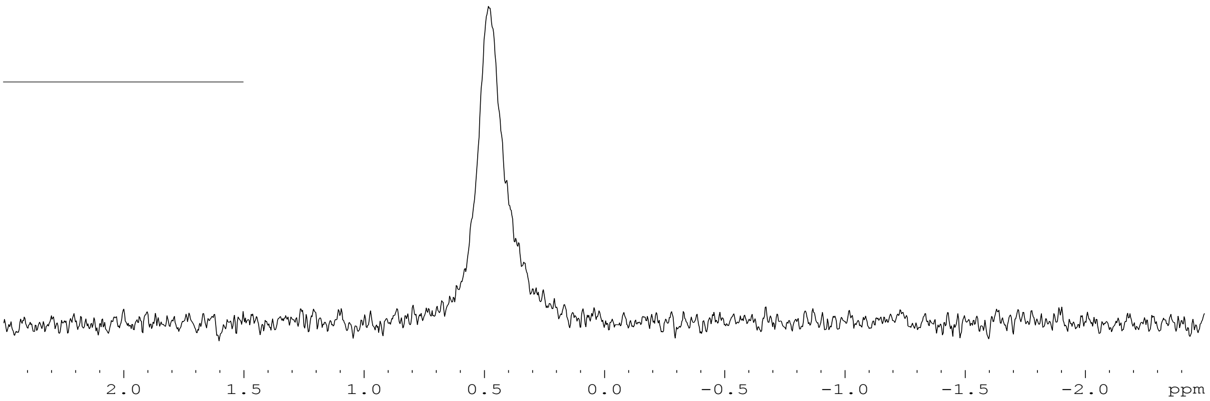

fig. S28d. ^{31}P SABRE-RELAY NMR spectrum for mono sodium dihydrogen orthophosphate (top) and expansion (bottom).

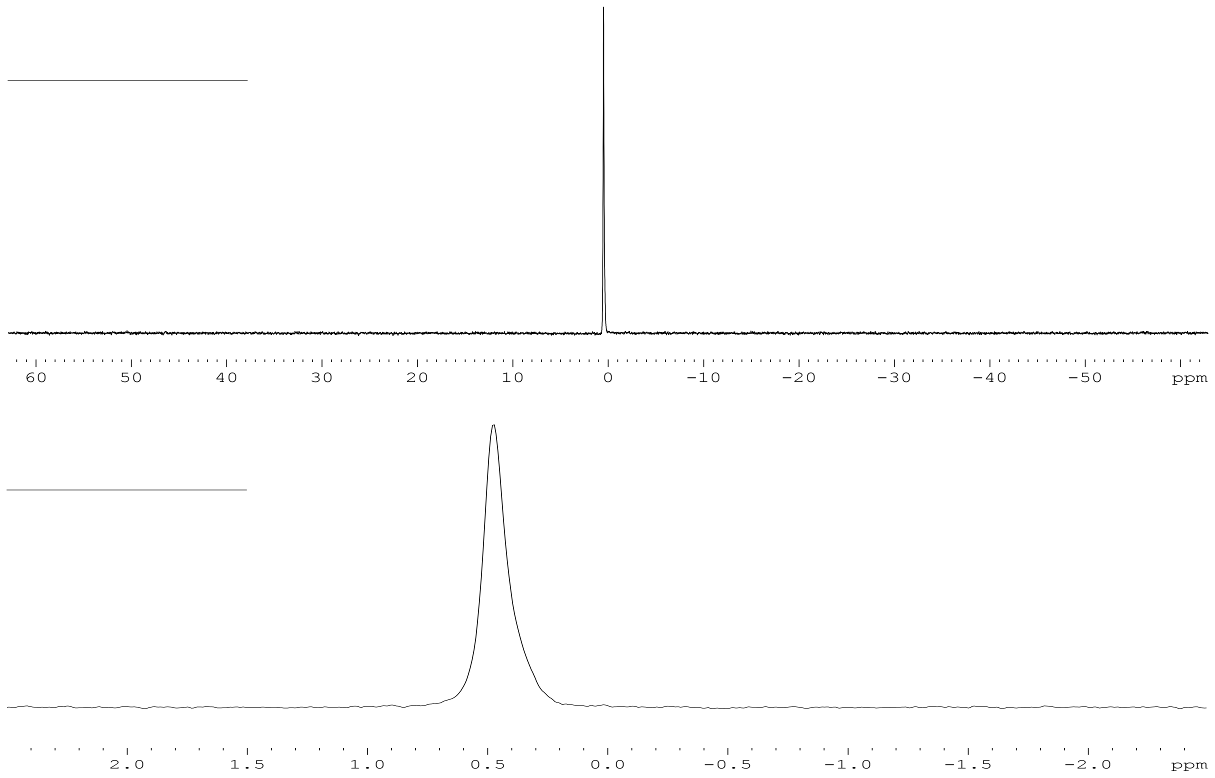

fig. S28e. ^{1}H-^{31}P SABRE-RELAY refocused INEPT NMR spectrum for mono sodium dihydrogen orthophosphate (top) and expansion (bottom). J_{XH} value used was 35.

Adenosine 5'triphosphate disodium salt

fig. S29. SABRE-RELAY NMR spectra adenosine 5′-triphosphate disodium salt. (**a** to **b**) reflect a series of single scan NMR spectra: (a) ^{31}P NMR spectrum thermally polarized and (b) ^{31}P SABRE-RELAY refocused INEPT NMR spectrum for Adenosine 5'triphosphate disodium salt using conditions: [IrCl(COD)(IMes)] (5 mM), Adenosine 5'triphosphate disodium salt (10 eq.),CD_2Cl_2 (0.6 mL), NH_3, H_2O (10 µL); Substrate Concentration: 50 mM.^{31}P enhancement (fold/per ^{31}P nuclei): 48, 32, 20.

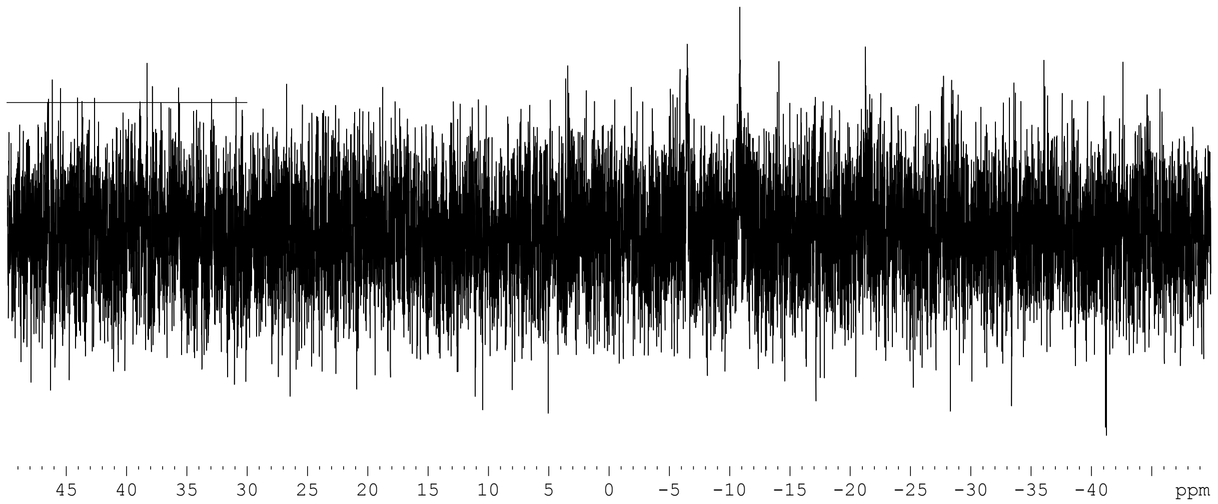

fig. S29a. Thermally polarized ^{31}P NMR reference spectrum for Adenosine 5’triphosphate disodium salt.

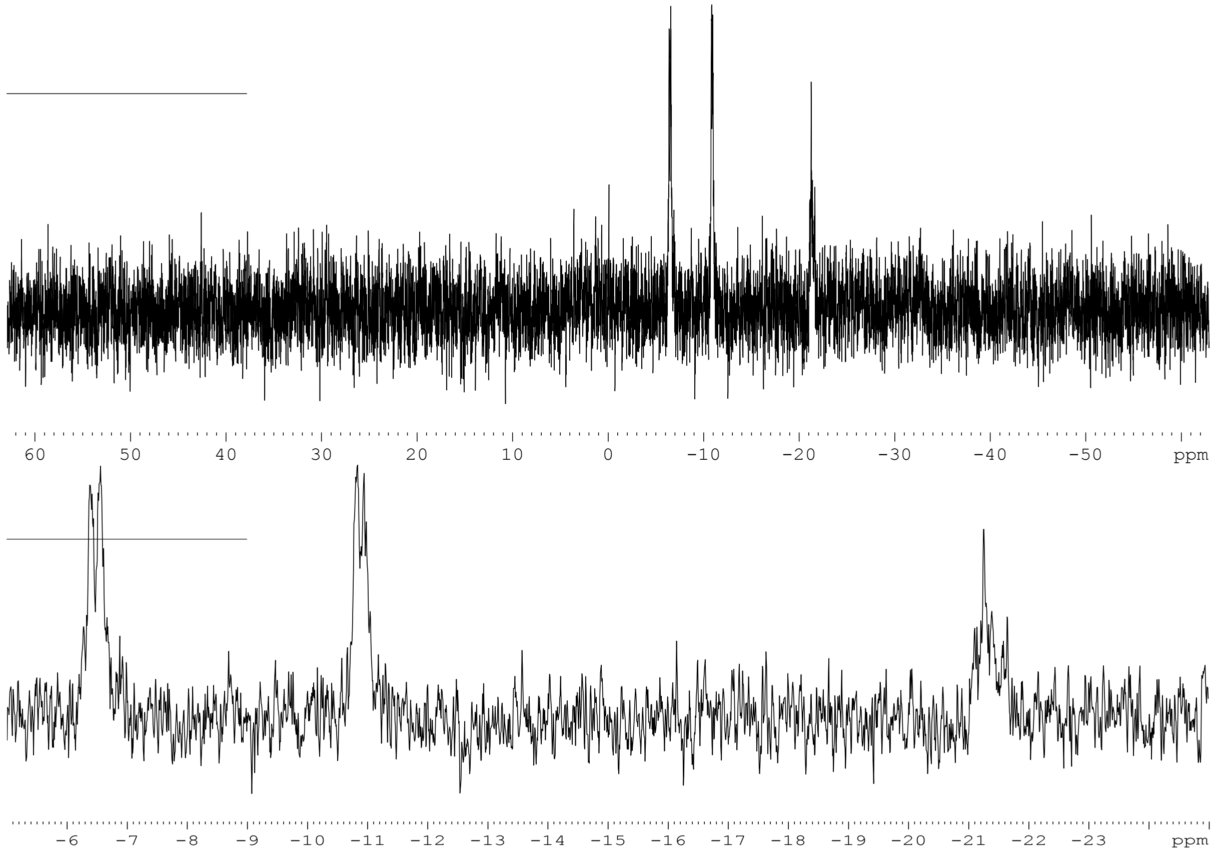

fig. S29b. ^{1}H-^{31}P SABRE-RELAY refocused INEPT NMR spectrum for Adenosine 5’triphosphate disodium salt (top) and expansion (bottom). J_{XH} value used was 35.

Amines

Ammonia in CD_3OD

fig. S30. SABRE-RELAY NMR spectra ammonia in methanol. Reflect a series of single scan NMR spectra of ammonia in CD_3OD: (**a**) ^1H NMR spectrum thermally polarized, (**b**) after 1 min of the activation, the CD_3OH hyperpolarizes through H/D exchange; using conditions: [IrCl(COD)(IMes)] (5 mM),CD_3OD (0.6 mL), NH_3.

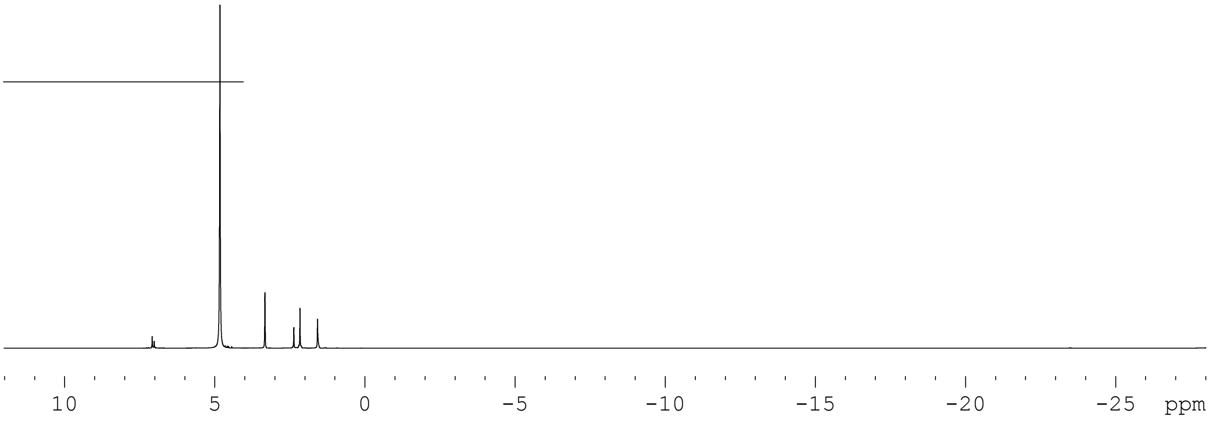

fig. S30a. Thermally polarized ^1H NMR reference spectrum for ammonia in CD_3OD.

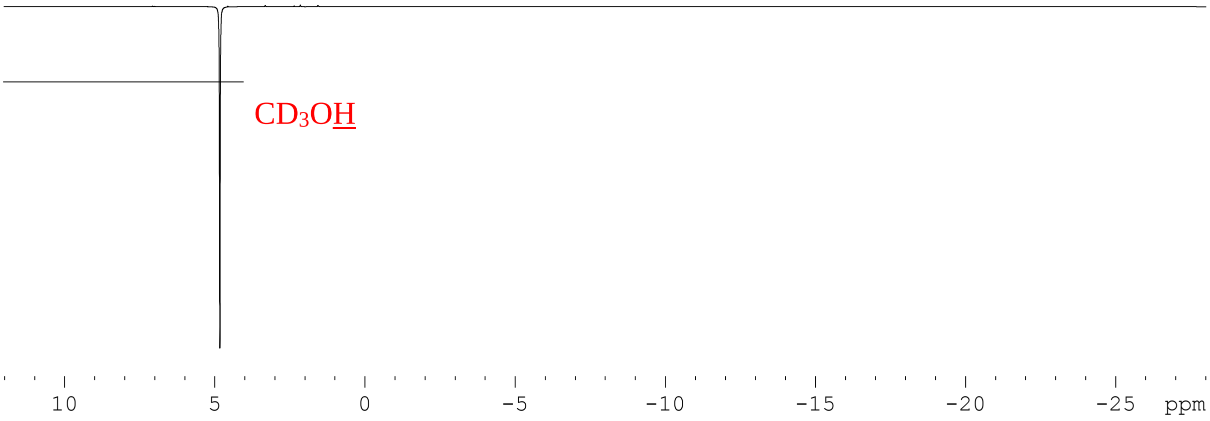

fig. S30b. ^1H SABRE-RELAY NMR spectrum for ammonia in CD_3OD show the hyperpolarization of $CD_3O\underline{H}$.

Fig. S30 - Thermally polarized ^1H reference NMR spectrum for ammonia in CD_3OD (a) alongside corresponding SABRE-RELAY spectrum (b).

Ammonia in CD_2Cl_2

fig. S31. SABRE-RELAY NMR spectra ammonia in dichloromethane. Reflect a series of single scan NMR spectra for ammonia in CD_2Cl_2: (**a**) ^{1}H NMR spectrum thermally polarized x16, (**b**) ^{1}H SABRE NMR spectrum hyperpolarization (max enhancement (fold/per proton) 94 fold) ; using conditions: [IrCl(COD)(IMes)] (5 mM),CD_2Cl_2 (0.6 mL), NH_3;

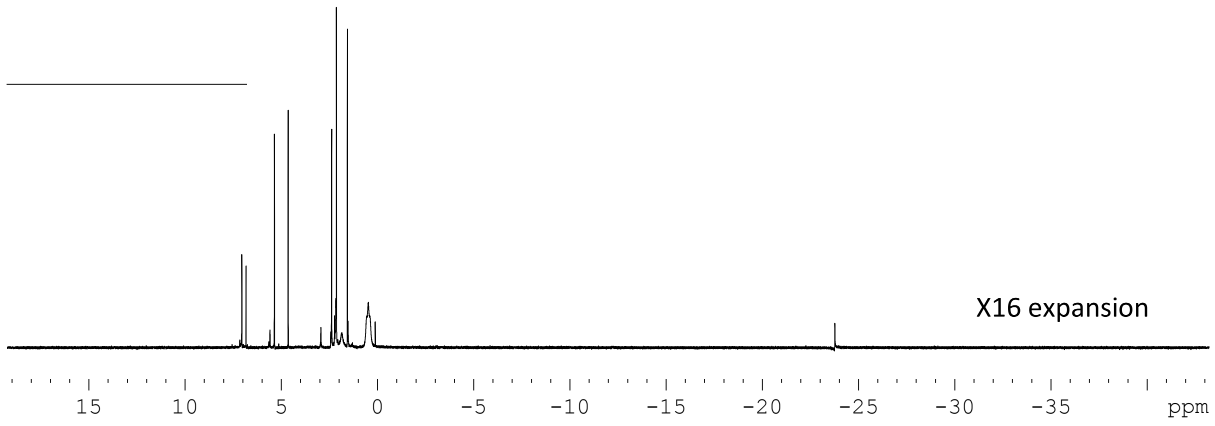

fig. S31a. Thermally polarized ^{1}H NMR reference spectrum for ammonia in CD_2Cl_2.

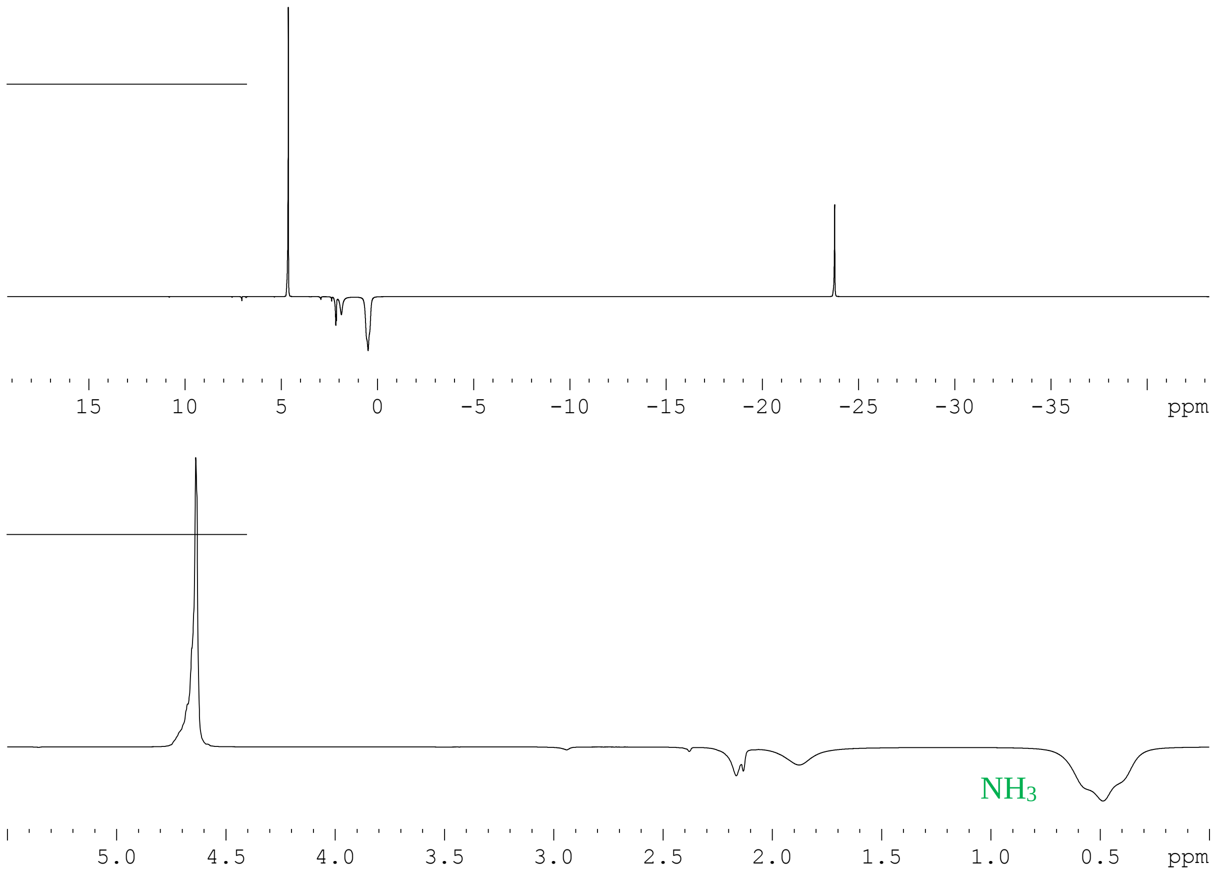

fig. S31b. ^{1}H SABRE-RELAY NMR spectrum for ammonia in CD_2Cl_2 (top) and expansion (bottom).

Benzylamine

fig. S32. SABRE-RELAY NMR spectra benzylamine. Reflect a series of single scan NMR spectra for benzylamine: (**a**) ^{1}H NMR spectrum thermally polarized x8, (**b**) ^{1}H SABRE NMR spectrum and (**c**) ^{1}H NMR spectrum after adding 1 ul of D_2O (max enhancement (fold/per proton) of H_2O 215 fold); using conditions: [IrCl(COD)(IMes)] (5 mM), benzylamine (10 eq.), CD_2Cl_2 (0.6 mL); Substrate Concentration: 50 mM. ^{1}H enhancement (fold/per proton): 61 (NH_2), 70 (-CH_2-), 36 (Ph).

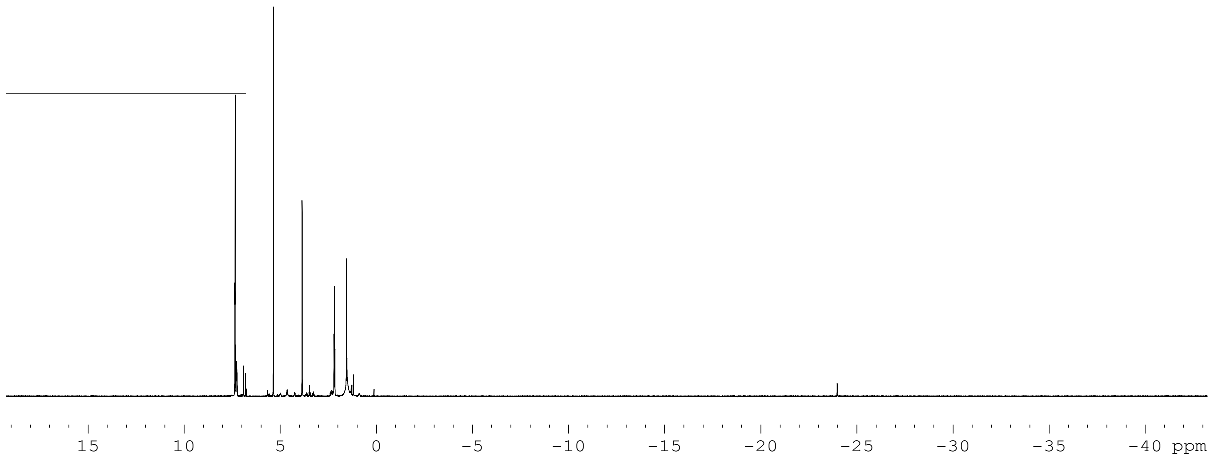

fig. S32a. Thermally polarized ^{1}H NMR reference spectrum for benzylamine.

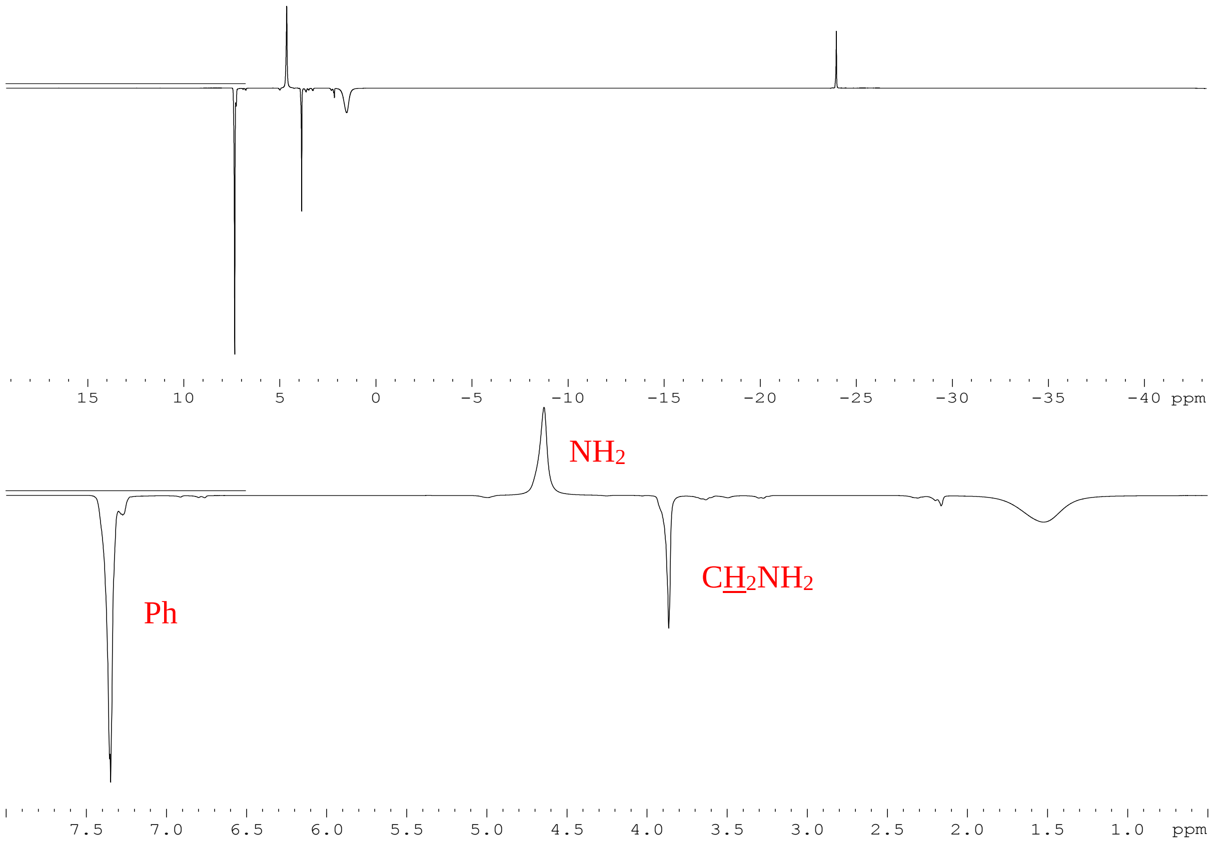

fig. S32b. ^{1}H SABRE NMR spectrum for benzylamine (top) and expansion (bottom).

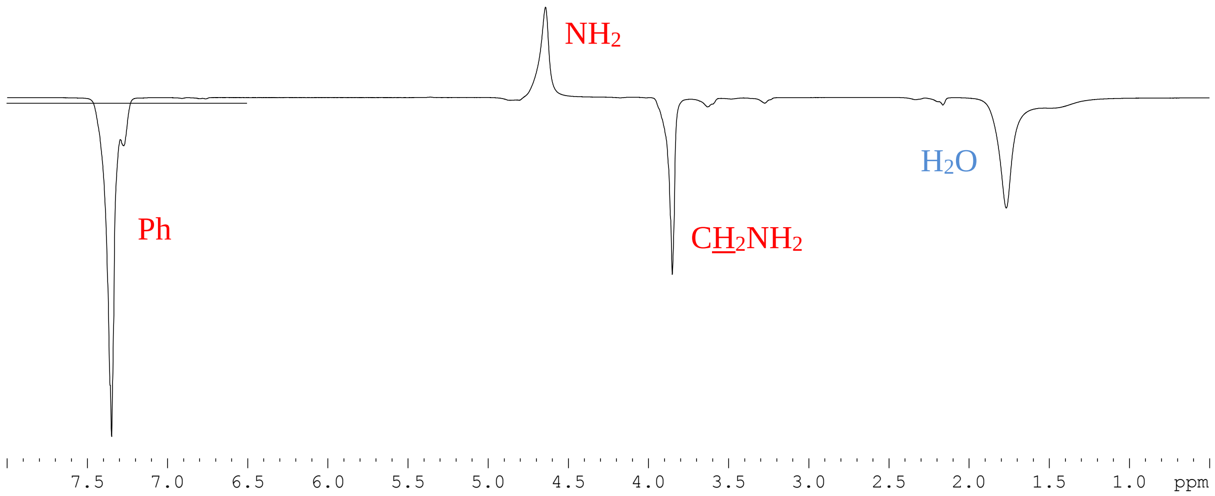

fig. S32c. ^{1}H SABRE NMR spectrum for benzylamine after adding 1 ul of D_2O.

Benzylamine-^{15}N

fig. S33. SABRE-RELAY NMR spectra benzylamine-^{15}N. Reflect a series of single scan NMR spectra for benzylamine-^{15}N: (**a**) ^{1}H NMR spectrum thermally polarized x4, (**b**) ^{1}H SABRE NMR spectrum and (**c**) ^{15}N SABRE NMR spectrum using conditions: [IrCl(COD)(IMes)] (5 mM), benzylamine-^{15}N(10 eq.),CD_2Cl_2 (0.6 mL); Substrate Concentration: 50 mM. ^{1}H enhancement (fold/per proton): 357 (NH_2), 248 ($-CH_2-$), 166 (Ph). ^{1}H enhancement (fold/per proton): 13 (NH_2), 10 ($-CH_2-$), 15 (Ph).

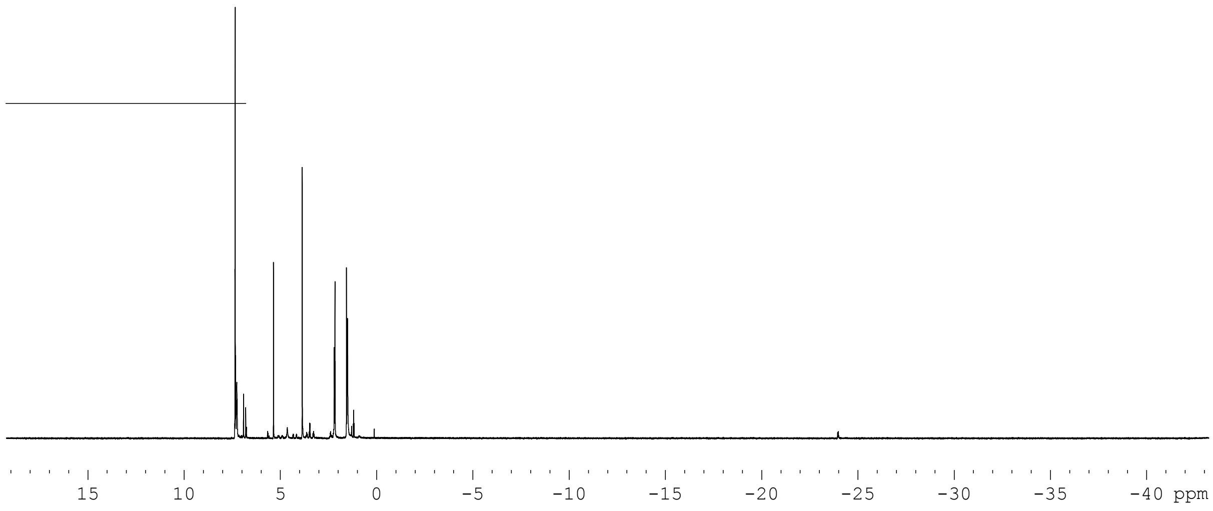

fig. S33a. Thermally polarized ^{1}H NMR reference spectrum for benzylamine-^{15}N.

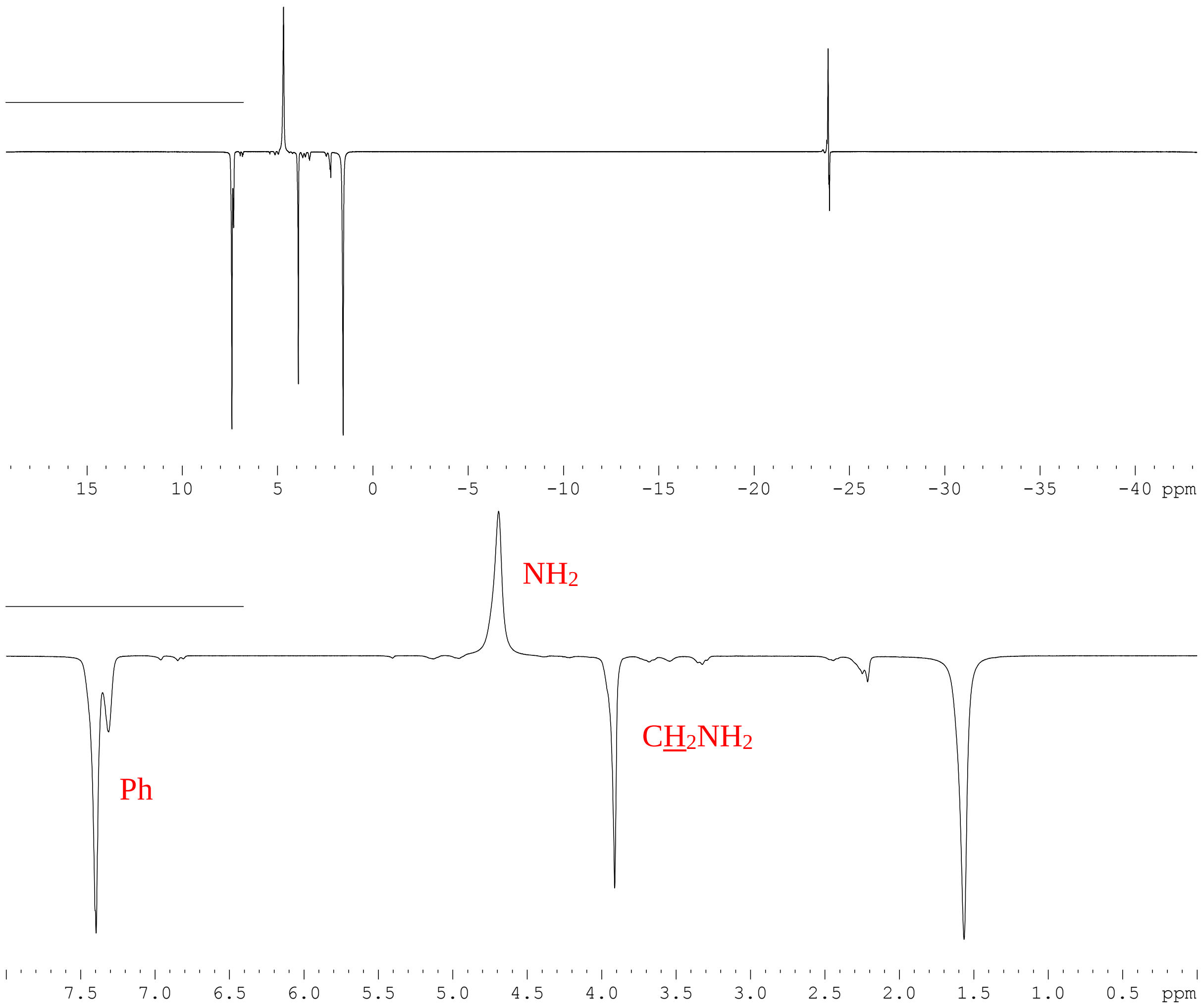

fig. S33b. ^{1}H SABRE NMR spectrum for benzylamine-^{15}N (top) and expansion (bottom).

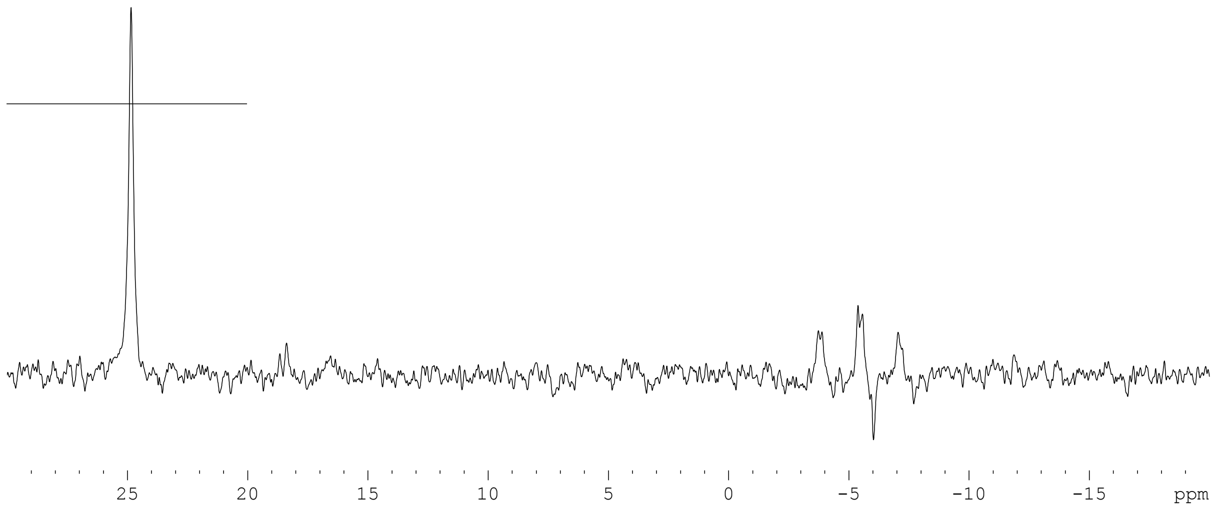

fig. S33c. ^{15}N SABRE NMR spectrum for benzylamine-^{15}N.

Substrate Mixtures

We have exemplified that SABRE-RELAY can be conducted on mixtures of substrates by combining the hyperpolarization of urea-^{13}C, 1-propanol and PEA. The recorded data shows polarization across all substrates.

fig. S34. SABRE-RELAY NMR spectra, mixture of urea, propanol, and PEA. (**a** to **d**) are the NMR spectra for (a) ^{1}H NMR spectrum thermally polarized, (b) ^{1}H SABRE-RELAY NMR spectrum, (c) ^{13}C NMR spectrum thermally polarized and (d) ^{13}C SABRE-RELAY NMR spectrum of a mixture of urea-^{13}C, 1-propanol and PEA using conditions: [IrCl(COD)(IMes)] (5 mM), Urea-1-^{13}C (10 eq.), 1-propanol (1 µL), PEA (1 µL CD_2Cl_2 (0.6 mL), H_2O (5 µL), NH_3;

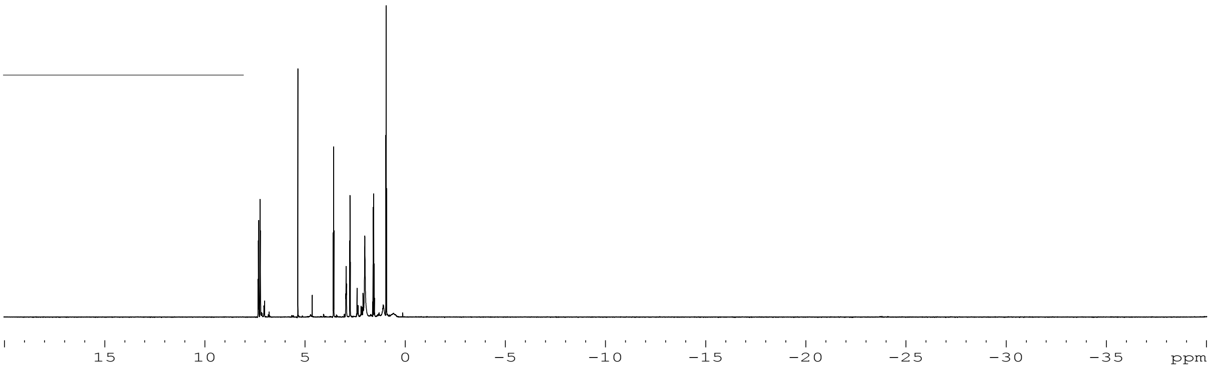

fig. S34a. Thermally polarized ^{1}H NMR reference spectrum for a mixture of urea, 1-propanol and PEA.

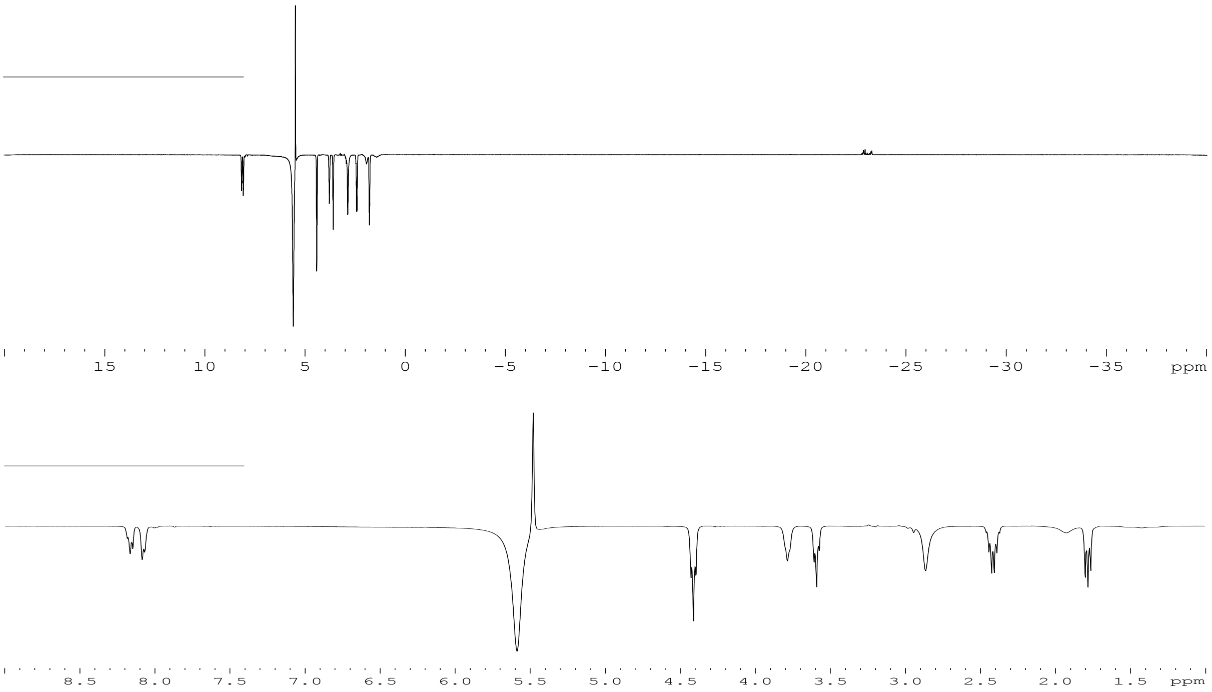

fig. S34b. ^{1}H hyperpolarized NMR spectrum for urea, 1-propanol and PEA (top) and expansion (bottom).

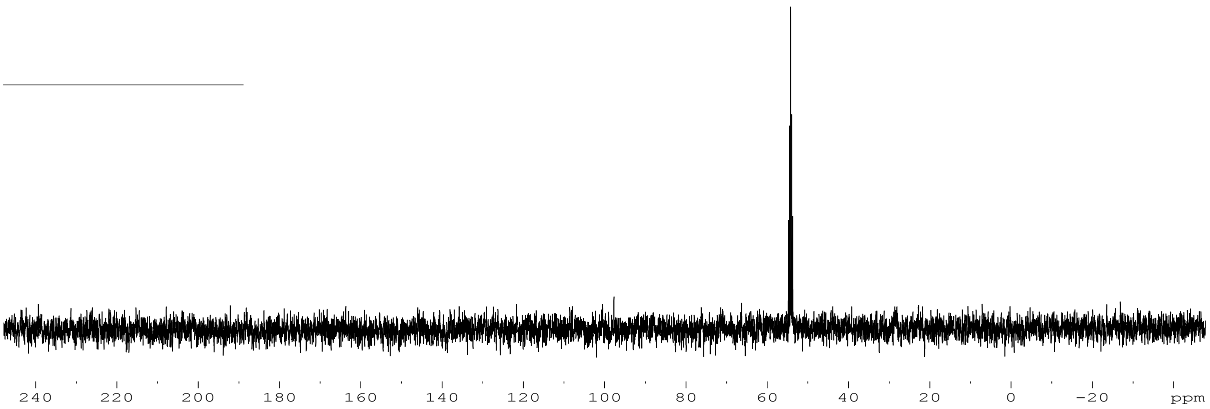

fig. S34c. Thermally polarized ^{13}C NMR reference spectrum for urea, 1-propanol and PEA.

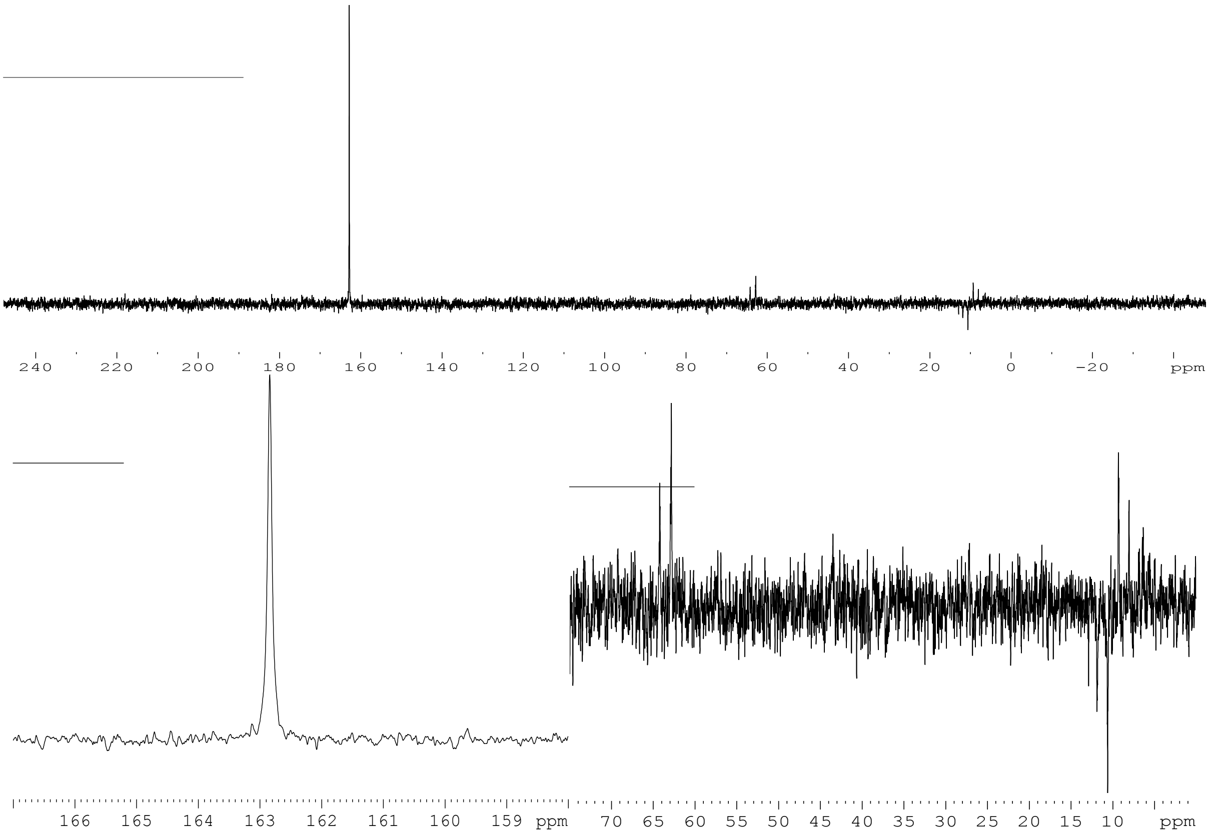

fig. S34d. ^{13}C hyperpolarized NMR spectrum for urea, 1-propanol and PEA (top) and expansion (bottom).

Characterization active SABRE-RELAY catalysts

Characterization of 2-NH_3

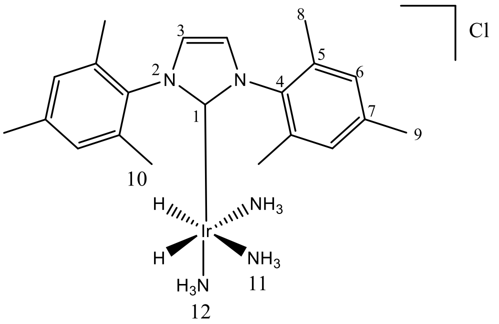

table S3. NMR data for 2-NH_3.

Resonance number	^{1}H (ppm)	^{13}C (ppm)	^{15}N (ppm)
**1**		153.8	
**2**			190.83
**3**	6.80	121.5	
**4**		138.11	
**5**		135.25	
**6**	7.00	129.00	
**7**		138.64	
**8**	2.10	18.30	
**9**	2.35	20.78	
**10**	-23.61		
**11**	2.21 (br, s, J_{NH} = 67 Hz)		-34.9
**12**	2.84 (br, s, J_{NH} = 69 Hz)		-46.5

Characterization of 2-$BnNH_2$

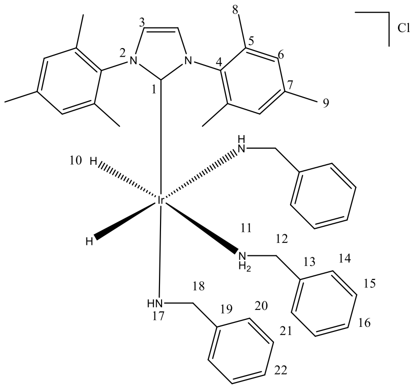

table S4. NMR data for 2-$BnNH_2$.

Resonance number	^{1}H (ppm)	^{13}C (ppm)	^{15}N (ppm)
**1**		153.8	
**2**			192.83
**3**	6.80	121.73	
**4**		138.11	
**5**		135.25	
**6**	6.90	129.29	
**7**		138.64	
**8**	2.17	18.30	
**9**	2.21	20.78	
**10**	-23.95		
**11**	5.00 (br, dt, J_{HH} = 5 and 11 Hz, 2H, J_{15NH} = 68 Hz)<br>2.30 (br, t, J_{HH} = 11 Hz, 2H, J_{15NH} = 68 Hz)		-6.4
**12**	3.65 (dt, J_{HH} = 5 and 15 Hz, 2 H), 3.30 (ddd, J_{HH} = 3, 12 and 15 Hz, 2H)	53.14	
**13**		141.8	
**14**	7.35 (d, J_{HH} = 5 Hz)	128.33	
**15**	7.25 (t, J_{HH} = 5 Hz)	126.88	
**16**	~7.28 - overlap	-	
**17**	4.25 (t, J_{HH} = 7 Hz, 2H, J_{15NH} = 69 Hz)		-13.4
**18**	3.85 (t, J_{HH} = 7 Hz, 2H)	57.26	
**19**		-	
**20**	7.30	128.35	
**21**	~7.28 - overlap	-	
**22**	~7.28 - overlap	-	
